# Advanced Microwave Processing for Next-Generation Materials

**DOI:** 10.1021/acsami.6c01811

**Published:** 2026-05-22

**Authors:** Prithvi Ravi, Amin Nozariasbmarz

**Affiliations:** Department of Mechanical Engineering, 3536 Rowan University, Glassboro, New Jersey 08028, United States

**Keywords:** microwave processing, electromagnetic field interaction, non-thermal effects, materials synthesis, non-equilibrium processing, decrystallization, thermoelectric materials, high-temperature ceramics

## Abstract

The next generation of technological innovations demands viable synthesis, processing, and manufacturing techniques. Conventional approaches, relying on traditional heating methods, are often energy-intensive, time-consuming, and costly. Furthermore, they face persistent challenges such as nonuniform heating, processing inefficiency, and limitations in achieving desired structures and properties. The interaction of electromagnetic (EM) fields, such as microwaves, with materials, offers an alternative technique to address these limitations, enabling numerous discoveries in the field of materials science. The term “microwave” refers to alternating EM signals within the frequency range of 300 MHz to 300 GHz, with corresponding wavelengths of 1 m to 1 mm. Unlike conventional heating, which relies on slow surface-to-core heat transfer via convection, radiation, and conduction, microwave energy interacts with materials at the atomic level, heating the entire volume simultaneously through volumetric EM energy absorption. This process is typically rapid and energy-efficient and is dictated by the material’s inherent transport properties. In addition, the unique, nonthermal effects of microwaves, including field-induced alloy decomposition, decrystallization, enhanced solid-state reactions, and defect generation, are particularly compelling. These phenomena are thermodynamically nonequilibrium and are essential for developing materials and structures with extraordinary properties. The combination of external microwave electric and magnetic fields can, in fact, result in unique material structures, such as amorphous, amorphous–crystalline, textured, and defective states. This paper reviews the basic concepts of microwave interaction with solid-state materials and recent advancements in emerging materials science fields, including advanced ceramics, batteries, renewable energies, carbonaceous materials, high-entropy alloys, and advanced processes like joining, 3D printing, and recycling. Ultimately, this work introduces advanced microwave processing as a powerful, cleaner, faster, and more effective strategy for the discovery, synthesis, and processing of next-generation materials.

## Graphical abstract

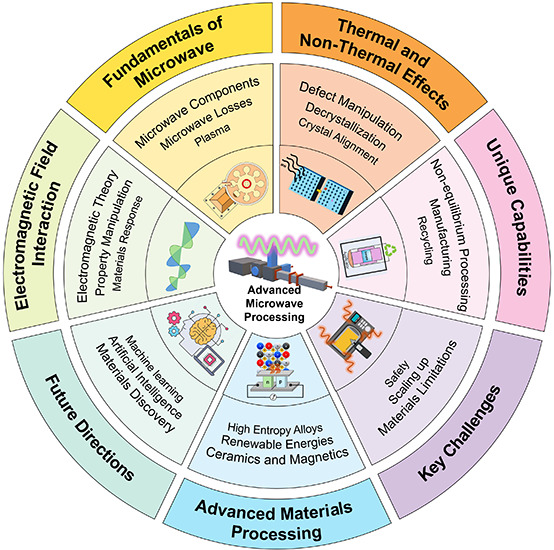

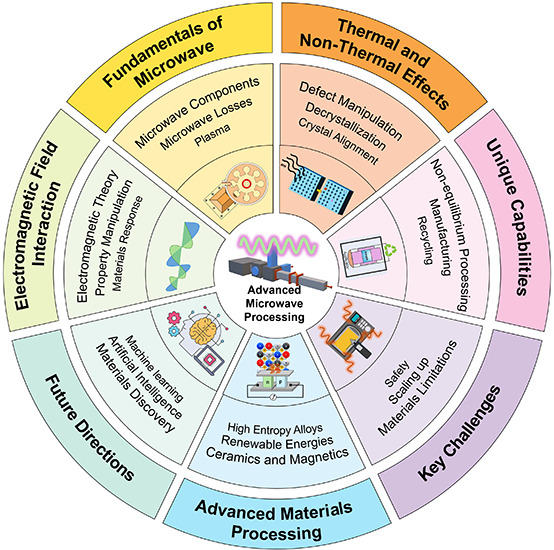

## 1 Introduction

Development of advanced materials has revolutionized key industries, from aerospace and energy to biomedical engineering and quantum technology. Translation of these materials into high-performance, application-ready components critically depends on processing methods that are both efficient and sustainable.^1^ Conventional heating routes, which rely on the slow and often uneven transfer of heat from an external source, are limited by issues like high temperatures, prolonged processing times, and energy waste. These limitations can lead to undesirable material outcomes, such as grain coarsening and phase degradation.^2–4^ These challenges highlight the urgent need for innovative processing strategies to unlock the full potential of next-generation materials.

Microwave processing has emerged as a promising alternative, offering unique advantages by offering pathways to fabricate high-performance materials with unique structure and properties, typically impossible to achieve by conventional techniques. Unlike traditional methods, microwaves excel at volumetric heating, uniformly raising the temperature throughout the entire material. This direct conversion of microwave energy into heat within the material dramatically reduces processing times and significantly decreases energy consumption.^5^

Initially invented in 1940 at Birmingham University in the UK, microwaves, a form of electromagnetic radiation, were first used by the military to detect enemy aircraft by emitting radar during World War II.^6^ By 1946, the technology was adapted for the food industry, where microwave energy was converted to heat due to the vibration of polar water molecules, causing rapid heating and cooking.^6,7^ However, due to their high cost initially, they did not become domestically available until the late 1970s after the prices decreased. For decades, microwaves have been utilized in the food industry by converting microwave energy into heat. This process works because polar water molecules vibrate due to electromagnetic interactions, causing rapid heating and cooking. Beyond conventional thermal processing, microwave irradiation enables selective energy coupling with microorganisms embedded in food, resulting in preferential heating relative to the surrounding medium and facilitating effective sterilization. Microwaves are also used for drying food. High-frequency electromagnetic waves facilitate evaporation of liquid moisture, transporting it to the surface of the food, which results in drying of the water molecules. This process is sometimes combined with vacuum or far-infrared techniques.^7^

Microwave irradiation was first pioneered for chemical synthesis in the mid-1980s independently by Gedye et al.^8^ and Giguere et al.^9^ Compared to traditional reflux methods, they reported an increase in reaction rates and improved yields. Their contribution was later expanded into inorganic synthesis, polymer science, nanotechnology, and catalytic materials design, where microwave irradiation is utilized to control reaction kinetics and tailor material structures.^10,11^ As the field matured, researchers transitioned from merely observing accelerated kinetics to investigating the underlying mechanisms. Notably, significant differences in reaction kinetics under microwave fields were observed compared to conventional synthesis, even when identical bulk temperatures were maintained.^12^ Such phenomena, which cannot be explained by bulk temperature alone, were described as “specific microwave effects”, now commonly referred to as nonthermal effects.^13^ In solid-state materials, these principles were later extended to the interfacial polarization mechanism (Maxwell–Wagner-Sillars effect). Various energy dissipation pathways, including dipolar, conduction (ohmic), ionic, interfacial polarization, and magnetic (hysteresis) losses, serve as the governing mechanisms in microwave-matter interactions.^14^

Today, microwave processing has found applications across various domains beyond its initial use in communication and food industry, as demonstrated in Figure 1a. They have been employed in processing and manufacturing of materials,^15^ satellite communication and mobile networks,^16^ cancer treatments,^17^ microwave ablation procedures,^18^ microwave imaging, biochemical sensing, signal transmission for implants and wearable devices.^19,20^

**Figure 1:**
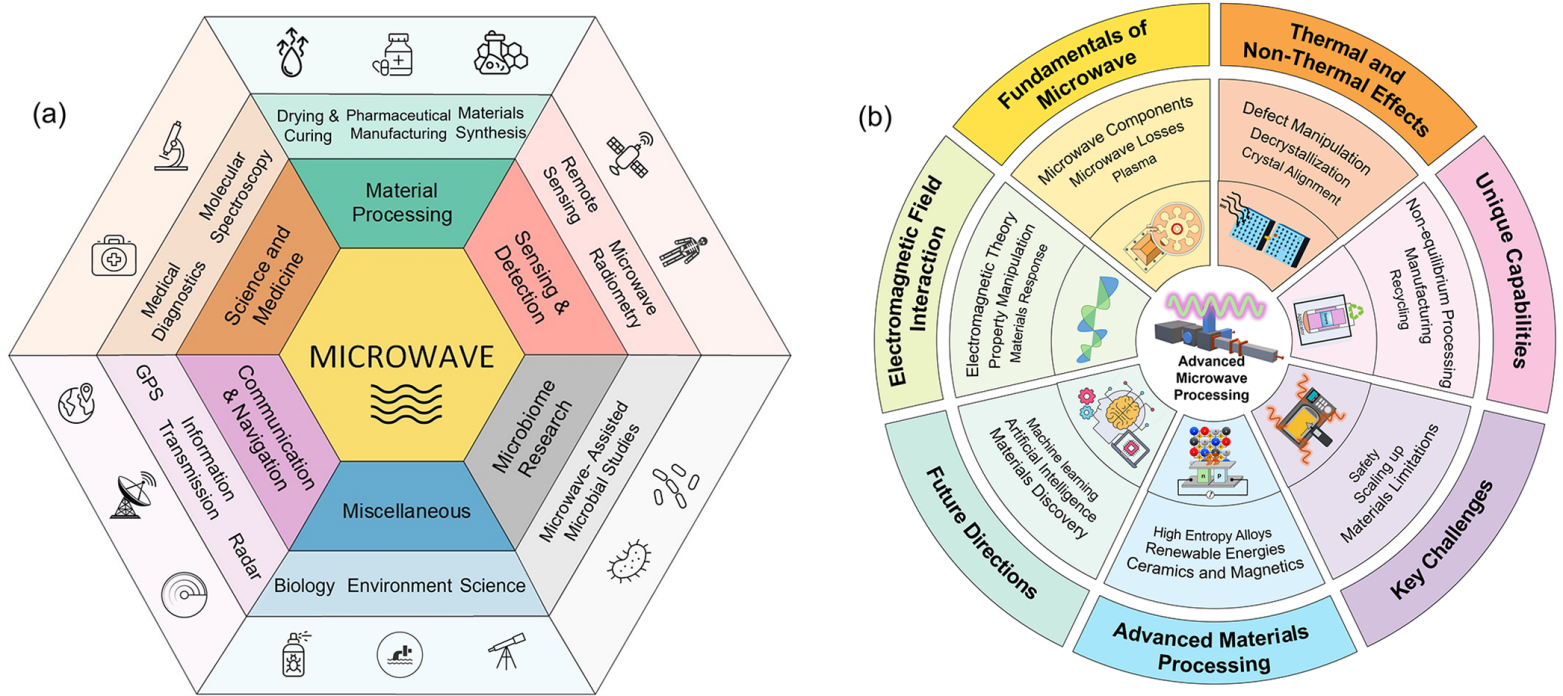
(a) Summary of the applications of microwaves in various fields. (b) Roadmap for advanced microwave processing and predictive materials design.

Early research into the microwave processing of engineering materials focused solely on microwaves as heating tools, recognizing their capacity for achieving much faster rates and higher energy efficiency compared to conventional methods, rather than as a tool for developing advanced materials. Subsequent research continued to emphasize the thermal aspects, focusing primarily on improving heating efficiency to reduce production costs and shorten processing cycles.^5^ Experimental studies on ceramics further demonstrated improved densification rates and reduced grain growth under microwave exposure. These findings strengthened the belief that microwaves function as unconventional furnaces, offering uniform and volumetric heating through direct energy deposition within the material.^21^

Recently, several observations challenged the interpretation of microwaves as merely faster heating tools. Emerging research in solid-state synthesis and materials processing reported effects such as rapid reaction kinetics, selective heating of specific phases, and the formation of nonequilibrium structures, phenomena that could not be explained by thermal models alone.^15,22^ These anomalies prompted a broader discussion on how electromagnetic fields interact directly with matter, extending beyond conventional heat transfer. This shift in understanding laid the foundation for studying nonthermal, field-driven, and far-from-equilibrium effects in solid-state manufacturing.^23,24^

Several review articles have previously discussed microwave-assisted heating, microwave synthesis, and applications in specific areas such as chemistry, ceramics processing, and catalysis. However, a comprehensive perspective that connects the fundamental physics of microwave-matter interactions with emerging solid-state materials systems and field-driven nonthermal effects remains limited. The research on the application of microwave processing of advanced solid-state inorganic materials has been limited, even though it possesses the potential to significantly improve their properties. Critical knowledge gaps persist regarding the distinct roles of the electric and magnetic fields, the influence of microstructure and composition on absorption behavior, and the complex nature of nonthermal effects during microwave interactions with materials. This paper addresses these gaps by connecting the fundamental physics of microwave interaction across various material systems. By transitioning from the conventional view of microwaves being a faster heat source, this review establishes microwave processing as an indispensable tool for predictive materials design. This capability directly leads to accelerated materials discovery, efficient synthesis, and support for next-generation manufacturing. This paper provides a comprehensive review of advanced solid-state microwave processing, specifically focusing on the capabilities of nonthermal effects, the unique resulting properties, and the processing of trending advanced energy, functional, bio, electronic, 3D printing, reduction of oxide, and recycling materials. By synthesizing this information, we aim to unlock the potential of solid-state microwave processing and shape future research directions for discovering materials with unique structures and properties. Figure 1b illustrates a strategic roadmap for transitioning microwave processing from a simple heat source to a tool for predictive materials design.

## 2 Fundamentals of Microwave and Its Interactions

Microwaves occupy the frequency range between 300 MHz and 300 GHz within the electromagnetic spectrum and are classified as a subset of radio waves (Figure 2a).^25^ Microwaves, like any other electromagnetic waves are composed of perpendicularly propagating, interdependent electric (E−) and magnetic (H−) field components, forming a transverse wave structure (Figure 2b). E-field originates from stationary charges, whereas H-field is generated by moving or oscillating charges. These coupled fields directly interact with matter through mechanisms such as polarization, dielectric relaxation, and ionic conduction, which will be discussed in subsequent sections.

**Figure 2:**
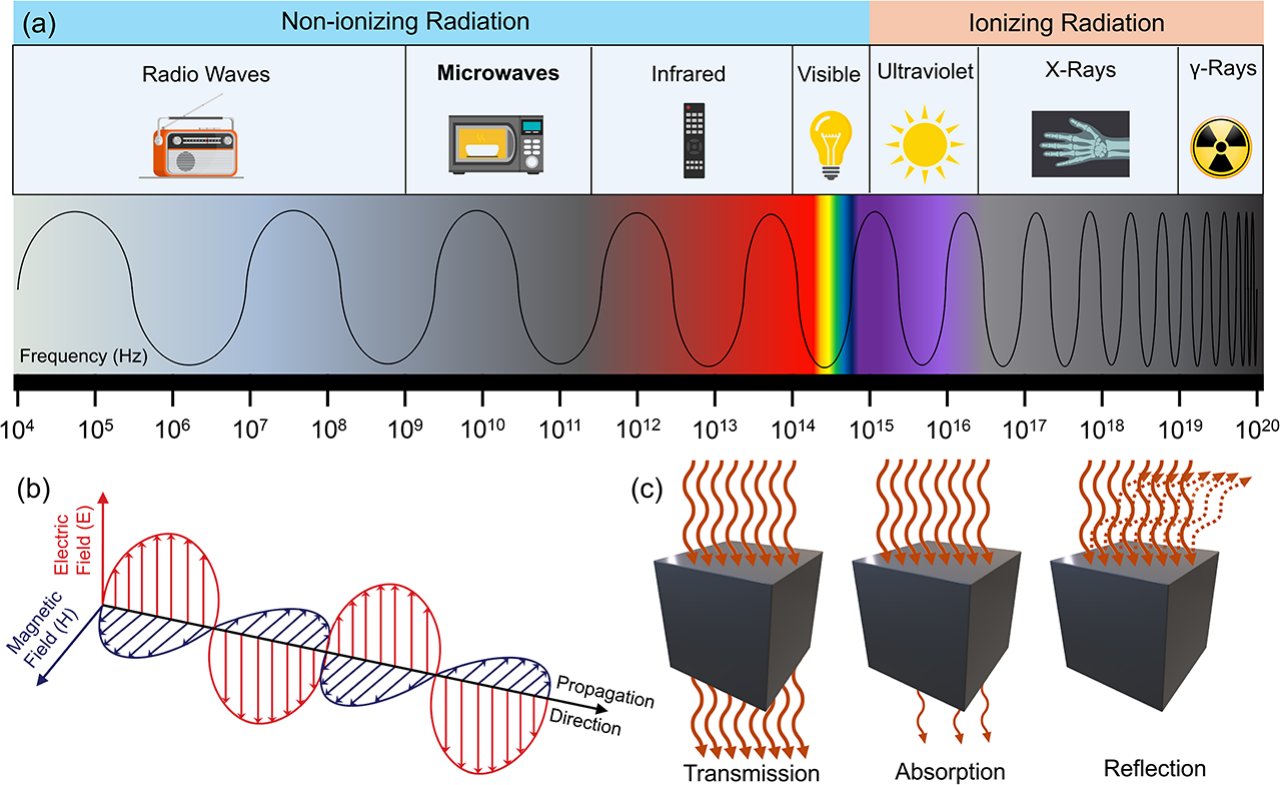
(a) Electromagnetic spectrum highlighting the microwave region. (b) Illustration of electromagnetic field propagation in space. (c) Schematic drawing of the interaction of electromagnetic fields with materials, including transmission, absorption, and reflection.

Electromagnetic fields are fundamentally governed by Maxwell’s equations, which describe how electric charges and currents give rise to E- and H-field and how they interact with each other dynamically. These equations established the intrinsic coupling between time-varying E- and H-field, allowing the waves to propagate through space. The solution to Maxwell’s equations inherently predicts the speed of light in vacuum. The equations are given below:

1 ∇·D=ε∇·E=ρ

2 $$\nabla \times E=-\frac{\partial B}{\partial t}=-\mu \frac{\partial H}{\partial t}$$

3 $$\nabla \times H=\frac{\partial D}{\partial t}+J=\varepsilon \frac{\partial E}{\partial t}+\sigma E$$

where *D* (=*εE*) is the electric flux density (in C·m^–2^), *ε* is the dielectric constant (F·m^–1^), *E* is the electric field strength (V·m^–1^), and ρ is the electric charge density (C·m^–3^). *B* is the magnetic flux density (T), *t* is time (s), μ is the magnetic permeability, (H·m^–1^), *H* is the magnetic field intensity (A·m^–1^), related to the magnetic flux density by B = *μH*, J (= *σE*) is the current density (A·m^–2^), and σ is the electrical conductivity (S·m^–1^).^26^

Electromagnetic waves (Figure 2b) consist of oscillating E and H fields which propagate through space at the speed of light. These waves can travel through various media such as solids, liquids, and gases, where their interaction with matter strongly depends on electromagnetic properties of the medium. These waves have a characteristic wavelength, determined by its frequency. The wavelength and frequency are inversely related to each other as c = λf, where c is the speed of light, λ is the wavelength and f is the frequency of the waves.

When the frequency of the incident electromagnetic waves coincides with the natural resonant frequency of the materials, the absorption of microwaves greatly increases. This promotes efficient energy transfer into the materials, promoting rapid heating.^26^

The material properties influencing the field interactions and the behavior of materials under microwave radiation is discussed in the following sections.

### 2.1 Material Properties Influencing Electromagnetic Field Interactions

Behavior of materials in an EMF varies depending on their specific properties. In an alternating E-field, dipolar atomic species and molecules with electric dipole moments will resonate and vibrate along with the field.^27^

The way a material heats up in a microwave depends on its inherent nature and the conditions of the microwave irradiation. Key factors that influence microwave absorption include dipole moment, dielectric permittivity (ε), dielectric constant (ε′), dielectric loss factor (ε″), electrical conductivity (σ), magnetic permeability (μ), polarizability, mass density, heat capacity, and thermal conductivity. These properties collectively determine how materials couple with the oscillating electric and magnetic fields of the incident microwaves, influencing whether the material transmits, absorbs, or reflects the incident energy.^15^

At low frequencies or when DC (direct current) is applied, dielectric performance of a material is described by a unitless number known as dielectric constant or relative permittivity (ε_r_). This is defined as the ratio of the material’s permittivity (ε) to the permittivity of free space (ε_0_). In these conditions, dielectric constant and relative permittivity can be used interchangeably. However, in high-frequency alternating fields, such as microwave electromagnetic field, the relative permittivity (ε_r_) is replaced by a complex relative permittivity, represented as $\varepsilon_{r}^{*}=\varepsilon_{r}^{\prime }-j\varepsilon_{r}^{\prime \prime }$ . Here, $\varepsilon_{r}^{\prime }$ is the real part, which is equivalent to the material’s dielectric constant or relative permittivity (ε_r_). It represents the material’s ability to store electric field energy (or polarization) and mathematically corresponds to the out-of-phase component of the current. $\varepsilon_{r}^{\prime \prime }$ is the imaginary part, known as the dielectric loss or loss factor. It represents the energy dissipated as heat and mathematically determines the in-phase (or loss) component of the current. As a result, $\varepsilon_{r}^{*}$ provides a comprehensive description of a material’s electromagnetic behavior, including both its energy storage and energy loss capabilities.^5,28,29^

Dielectric loss tangent (tan δ), as the relative magnitude of $\varepsilon_{r}^{\prime \prime }$ with respect to, i.e., $\varepsilon_{r}^{\prime },\;i.e.,\;\tan\delta =\frac{\left(\varepsilon_{r}^{\prime \prime }\right)}{\left(\varepsilon_{r}^{\prime }\right)}$ , is another key parameter in microwave processing of materials. It measures the phase lag between an applied electromagnetic field and material’s polarization response. Essentially it quantifies how efficiently a material absorbs microwave energy and converts it into heat at a given frequency. It is defined as the ratio of energy lost (as heat) to energy stored. tan δ is mainly used for materials that absorb or transmit microwave, and not for reflectors. When tan δ is zero, there is no energy loss. If tan δ is greater than zero, some microwave energy is lost as heat. Materials with a low loss factor (around 0.0001–0.01) are transparent to microwave, while those with high loss factor (above 0.1) absorb significant microwave energy and convert it to heat. Materials with a medium loss factor (from 0.01 to 0.1) are ideal for controlled microwave processing.

### 2.2 Materials Behavior under Microwave Radiation

Based on the interactions with microwave radiation, materials are generally divided into three types: transparent, absorptive, or reflective (Figure 2c).

Microwave Transparent Materials: In general, materials with a low relative dielectric constant (ε_r_), low loss tangent (tan δ), and low electrical conductivity (σ) are microwave transparent. This includes insulating materials with wide bandgaps and negligible dielectric loss. In these materials, microwave energy propagates through the material with minimal interaction or energy dissipation. Such materials do not experience significant heating. Examples of such materials include fused silica (SiO_2_), quartz, boron nitride, PTFE, and polyethylene.

Microwave Absorbing Materials: Materials with moderate to high *ε*_*r*_ and high tan δ act as microwave absorbers. These materials have finite dielectric or magnetic loss, and microwave energy is dissipated through mechanisms such as dipolar polarization, ionic conduction, interfacial polarization, and magnetic relaxation (these mechanisms will be discussed in Section 4.1). Examples of such materials include water and various carbon composites.

Microwave Reflective Materials: Materials having a high or infinity *ε*_*r*_ along with high electrical conductivity are microwave reflectors. In these materials, free electrons effectively shield the interior from the incident EMFs. Examples include copper and aluminum.^26^ Table 1 compares the dielectric properties of various types of materials within a microwave frequency and alternating field. It is important to note that both the dielectric constant and the loss tangent of materials can change with frequency.

**Table 1:** Relative Permittivity (ε_r_), Loss Tangent (tan δ), and Microwave Behavior of Materials (1–10 GHz)^29–32^

Material	Relative Permittivity (ε_r_)^a^	Loss Tangent (tan δ)^a^	Microwave Behavior
Air	1.0005	∼0	Fully transparent
PTFE (Teflon)	2.1	0.0002	Extremely low loss, excellent transparency
Polyethylene (PE)	2.3	0.0004	Very low loss, transparent
Polystyrene	2.5	0.001	Very low loss, transparent
Paper	3	0.008	Very low loss, transparent
Kaptan	3.4	0.002	Very low loss, transparent
Fused Silica (SiO_2_)	3.8	0.0001	Very low loss, transparent
Quartz (α-SiO_2_, Crystalline)	3.8–4.5	0.0001	Low loss, transparent, stable at high temperature
Boron Nitride (h-BN)	4.0	0.0002	Low loss, transparent, stable at high temperature
Diamond	5.68	<0.0001	Low loss, transparent, stable at high temperature
Aluminum Nitride (AlN)	8.9	0.0005–0.002	Low loss, limited absorption in pure AlN
Magnesium Oxide (MgO)	9.7	0.0001	Limited absorption, low loss
Alumina (Al_2_O_3_)	9.8	0.0001	Limited absorption
Pure Silicon	11.7	<0.001	Limited absorption, transparent when undoped
GaAs	12.85	0.0006	Low absorption
Ni Ferrite	13	<0.001	Low absorption
Hexagonal Ferrite	15	<0.01	Low absorption
Water (Distilled)	78	0.15	Very high absorption
Barium Titanate	85	3	Very high absorption
Lead Zirconate Titanate (PZT)	120–3400	0.001–0.038	Very high absorption
Aluminum	∞	-	Reflects almost all microwaves
Copper	∞	-	Reflects almost all microwaves

a The numbers may vary in different references.

#### 2.2.1 Skin Effect

When an EMF interacts with bulk conductive materials like Cu or Al, the induced current concentrates near the surface of the conductor. This phenomenon is known as the skin effect. As a result, microwave energy is largely reflected at the surface, with minimal field penetration defined by the skin depth (ξ). Only limited absorption occurs through surface through Joule heating.^33,34^ Skin depth is given by the eq 4:

4 $$\xi =\sqrt{\frac{\rho }{\pi \times f\times \mu_{r}\times \mu_{0}}}$$

where ρ is the electrical resistivity of the bulk sample, *f* is the frequency of the microwave, *μ*_*r*_ is the relative magnetic permeability, and μ_0_ is the magnetic permeability constant.^26^

#### 2.2.2 Penetration Depth

In solids, energy transfer occurs through resonance and relaxation processes, with the overall effect being the conversion of microwave energy into heat.^35,36^

During processing, materials absorb microwave energy, while any unabsorbed energy is either reflected or propagated. The Penetration Depth (PD) is the distance a microwave travels into a material before its power significantly decreases (to approximately 37% of its initial value).^37^ Microwave power decays exponentially within a material. The point at which the power drops to 37% corresponds to the Penetration Depth (PD) as *x =* PD. The PD is given by eq 5:

5 $$PD=\frac{\lambda_{0}}{2\pi \sqrt{\varepsilon^{\prime }\left[\sqrt{1+{\left(\frac{\varepsilon^{\prime \prime }}{\varepsilon^{\prime }}\right)}^{2}}-1\right]}}$$

where λ_0_ is the free-space wavelength of microwave radiation, *ε′* and *ε*″ are the real and imaginary parts of the complex relative permittivity of the material. The ratio *ε*″/*ε*′ is known as the loss tangent and quantifies the material’s ability to convert electromagnetic energy into heat. Materials with higher dielectric loss exhibit shallower penetration depths, indicating a more rapid microwave absorption and localized heating. According to eq 5, the PD in a material depends on several factors, including wavelength of radiation, dielectric properties and the magnetic properties of the material.^21^

## 3 Basics of Microwaves—Microwave Components

Microwave processing relies on three primary compartments: microwave generator, applicator, and waveguide. These components are essential for generating, transmitting, and effectively applying microwave energy on the materials.^26^ Manufacturers can customize microwave components for specific applications based on key factors such as the state of the materials (liquid or solid), processing size (small, medium, or large), desired temperature range, auxiliary components, and reactor configurations. enable manufacturers to customize microwave components for their desired applications. Figure 3a shows schematic image of a single-mode cavity microwave, which includes the generator, circulator, tuners, waveguide, microwave cavity, and the sliding short (or plunger). The fundamental principles and operation of these essential components will be discussed in detail.

**Figure 3:**
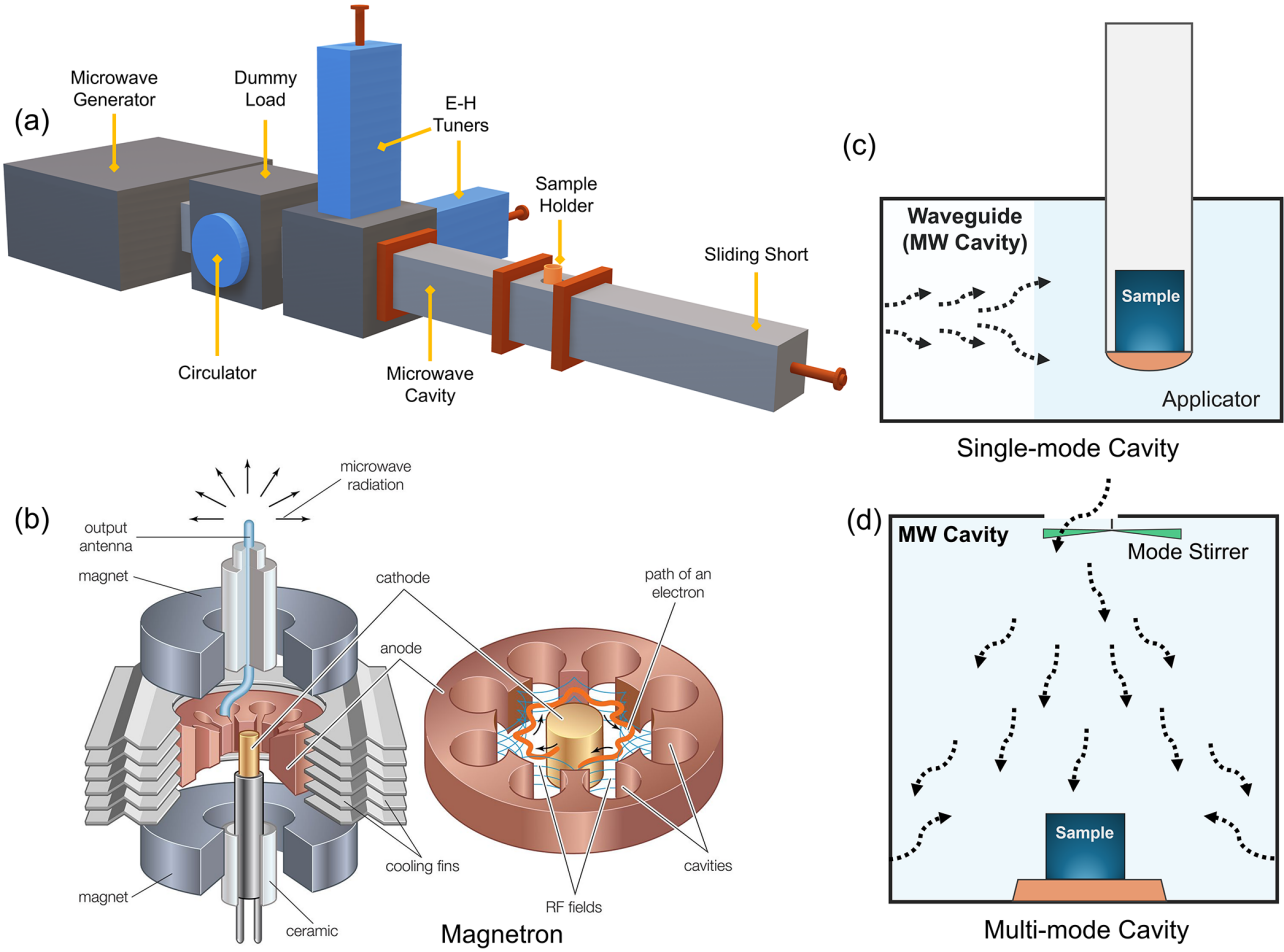
Schematic picture of microwave components: (a) Microwave processing setup, and (b) Magnetron and its units^48^ (By courtesy of Encyclopedia Britannica, Inc., copyright 2014; used with permission). Modes of microwave cavities: (c) Single-mode, and (d) Multimode.

### 3.1 Microwave Generators

The microwave generator produces electromagnetic radiation in the microwave region. The source of microwave generation is magnetron. A magnetron is a high-power oscillator that directly converts electrical energy into microwave radiation, making it the “heart” of a microwave system. Figure 3b shows the picture of a typical microwave magnetron.^38^

The main components of the magnetron include two magnets, an output antenna, an anode, a cathode, and cooling fins. The anode is a cylindrical copper block that controls the output frequency of the magnetron. It contains an even number of resonant cavities, which are connected to the center by a narrow slot and function as parallel resonant circuits. The cathode, located at the center of the anode, consists of a heater (or filament) surrounded by a hollow cylinder made of a high-emissive material. When heated, the cathode emits electrons through thermionic emission. Power supply provides a DC bias to accelerate these electrons from the cathode toward the anode.^39,40^

To create a magnetic field (H-field) in the space between the anode and cathode, two permanent magnets or electromagnets are positioned at the back and front of the anode. This H-field is perpendicular to the path of the emitted electrons and exerts a force on them, causing a cloud of electrons to move in a circular, spiraling path toward the positively charged anode. This spiral motion of electrons makes them resonate with the anode cavities at a specific frequency. Essentially, the magnetic force sweeps the electrons in a circle, pumping the natural frequency of the cavity. The currents created within these resonating cavities then radiate microwave energy at the resonant frequency. In order to prevent overheating and possible failure, the magnetron is cooled with fins or water circulation. The generated microwave energy (or radio frequency) is then collected by a short antenna connected to a waveguide which guides the energy to its load. The wall of the waveguide reflects the electromagnetic field and directs the wave into the cavity.

The other components of magnetron include a gasket for waveguide connection, a voltage input terminal, a filter as conductive noise suppressor, a yoke for steady magnetic field and magnetic circuit, mounting plate for waveguide installment, a stem for insulating and supporting the cathode, and filter box for microwave shielding.^40,41^

Microwave chemical reactions often operate at frequencies of 0.915, 2.45, and 5.80 GHz, which fall within the designated industrial, scientific, and medical (ISM) frequency bands,^42^ each offering distinct heating characteristics.^43^ As the frequency of the microwave increases, the wavelength shortens, leading to a decrease in the penetration depth into the materials. However, the heating efficiency tends to improve, and the heating zones become more localized.

The most common systems for both laboratory and industrial use operate at 2.45 GHz frequency due to a more balanced penetration depth and moderate heating efficiency. The 0.915 GHz microwaves with the longest wavelength and the lowest frequency offer deep penetration. However, they produce only about 19% power density compared to the 2.45 GHz systems. In contrast, the 5.80 GHz has the shortest wavelength, producing a power density roughly 7.46 times that of the 2.45 GHz frequency offering enhanced microwave heating.^26^ Table 2 compares different microwave frequencies and their applications.

**Table 2:** Comparison of Different Microwave Frequencies (0.915 GHz,^26^ 2.45 GHz,^26^ 5.8 GHz,^44^ 24 GHz,^45,46^ 300 GHz^47^) and Application Domains

Frequency	Penetration/Field Behavior	Processing Implication
0.915 GHz	Deeper penetration due to longer wavelengths, enabling energy deposition throughout large sample volumes	Suitable for large-scale microwave processing
2.45 GHz	Balanced penetration depth and energy absorption, allowing for effective volumetric heating across wide range of materials	Most widely used microwave processing frequency
5.8 GHz	Reduced penetration depth, leading to stronger near-surface energy deposition and higher field gradients compared to lower frequencies	Favorable for localized heating and thin materials
24 GHz	Reduced penetration due to shorter wavelength, leading to enhanced localized field effects	High power-density processing, used for surface engineering
300 GHz	Highly localized electromagnetic fields across smaller regions through beam focusing while also allowing for controlled field distribution	Used for precision and localized heating and surface modification

### 3.2 Waveguides

Waveguides are described as hollow conducting tube capable of guiding an electromagnetic wave.^49^ In free space, the electric and magnetic fields propagate toward the same direction, while their longitudinal components are perpendicular to each other. However, within the waveguide, when using magnetrons, microwaves do not travel linearly. We must force the electric field to move parallel to the short axis of the waveguide so that microwaves can be propagated inside the waveguide.

For microwaves with frequency of 2.45 GHz, the wavelength is 12.24 cm. However, the length of the microwaves changes to 14.78 cm inside the waveguide. This is known as guide wavelength (λg), which can be calculated as

6 $$\lambda_{g}=\frac{\lambda }{\sqrt{1+{\left(\frac{\lambda }{2a}\right)}^{2}}}$$

where λ is the free space wavelength and a is the length of the long axis of the waveguide. The electric field has zero intensity at the walls along the short axis but reaches maximum strength at the center along the long axis.^26^

Magnetic fields within the waveguides work in conjunction with the electric field within the waveguides. They propagate along with the electric field but are oriented perpendicular to the direction of the electric field. The magnetic field intensity is maximum when the electric field intensity is zero—near the waveguide walls. Like the electric field, magnetic field exhibits sinusoidal propagation pattern.

#### 3.2.1 Standing Wave

Within a waveguide, microwaves exist as sinusoidal waves. A standing wave is a stationary pattern of electric and magnetic fields that forms inside the waveguide when a propagated microwave signal and its reflection (for instance, from the sliding short) interfere. Their superposition creates alternation points of constructive interface, known as antinodes, where the field is at its maximum, and destructive interface, known as node, where field strength is zero.^50^

#### 3.2.2 Microwave Modes

As microwaves propagate through a waveguide, they form discrete electromagnetic field patterns known as modes, which arise due to the boundary conditions imposed by the geometry of the waveguide. Unlike in free space, where electric and magnetic fields propagate together freely, waveguides constrain the fields to specific spatial configurations. These modes fall into two main categories—Transverse Electric (TE) modes and Transverse Magnetic (TM) modes.

In waveguides, the direction of wave propagation is considered the z direction. In transverse electric modes, the electric field has no component in the direction of wave propagation (Ez = 0). But the magnetic field component (Hz) is nonzero. These modes are denoted as TE_mn_ modes, where m and n are the half-wave variations in the electric field in the transverse (x and y) directions.^51^ TE_10_ mode is the dominant mode in rectangular waveguides. It is commonly used at 2.45 GHz in microwave processing due to having the lowest cutoff frequencies.^52^ The cutoff frequency is the lowest frequency at which a waveguide mode can propagate. In contrast to the TE modes, TM modes have no magnetic field component in the propagation direction. So, H_z_ = 0. They are labeled as TM_mn_. They are less commonly used in industrial microwave processing because they generally have higher cutoff frequencies and are more sensitive to the specific boundary conditions of the waveguide.^26^

A key feature of these standing modes is the creation of spatially separated field distributions.^53^ This separation allows single-mode microwaves to create distinct regions where materials can be selectively exposed to either the electric or the magnetic fields. This capability is crucial, as it enables the isolation and understanding of the unique effects resulting from the interaction of the individual electric and magnetic fields with the material.

### 3.3 Isolator/Circulator

Isolators, also known as circulators, protect the microwave equipment from reflected waves by allowing the microwaves to only pass in only one direction—from source to applicator, but not vice versa. The device prevents them from traveling back toward the source. Some circulators are made with 3—ports, one connected to source, one to the applicator, and third to a dummy load such as water or ferrite. The circulator diverts the returned wave to the dummy load which absorbs the reflected power, preventing it from reaching the source.^40^

### 3.4 E-H Tuners and Plunger

E-H tuners are used in microwave equipment to match the impedance between the microwave generator and the material. They consist of metallic stubs or plungers that can be manually or automatically adjusted to modify the electric and magnetic field distributions. These tuners help by adjusting the system to handle samples with poor microwave absorption by changing the impedance. The plunger, also known as sliding short, is connected to the other end of the waveguide to shift the location of the end plate. This action adjusts the standing wave within the waveguide, which in turn moves the maxima of the field to the sample’s position, achieving a more uniform synthesis. Both E-H tuners and the plunger work continuously to adjust the field strength and position on the sample.^26^

### 3.5 Iris

In a microwave, iris is a thin metallic plate with an opening (aperture) at its center. Iris is installed in the waveguide close to the applicator and is used to control the propagation of microwave within the waveguide. The iris has various functions, including impedance matching, coupling, filtering, and power control.^26^

### 3.6 Microwave Cavity/Applicators

Microwave cavities are closed structures that confine the electromagnetic fields in the microwave region. They are designed to confine and distribute electromagnetic energy efficiently to the materials that are synthesized. An important parameter for evaluating the performance of a microwave cavity is the quality factor (Q), which quantifies how effectively the cavity stores energy in relation to its losses. It is the ratio of stored energy to the energy lost per oscillation cycle and is given by eq 7 ^54,55^

7 $$Q=2\pi \frac{\mathrm{energy}\;\mathrm{stored}\;\mathrm{in}\;\mathrm{the}\;\mathrm{cavity}}{\mathrm{energy}\;\mathrm{lost}\;\mathrm{per}\;\mathrm{cycle}}$$

Applicator is a specific section within a microwave cavity or waveguide where the sample is placed. This is the critical zone where microwaves directly interact with the material. The environment inside the applicator can be air, an inert gas, or a vacuum.^21^

There are two primary types of microwave cavities—single mode and multimode. These differ in their primary design, operating principles, and applications.

#### 3.6.1 Single Mode Cavity

The simplest single-mode cavities (applicators) usually consist of a waveguide that operates near the cutoff frequency. Figure 3c depicts a single mode applicator, which has a single microwave source and a rectangular or cylindrical cavity that can support one resonant mode. The cavity size is a factor of the wavelength of the microwave. Single-mode cavities usually have just one hot region where the microwave field strength is the highest. They also feature designated slots to facilitate the transfer of materials. These applicators tend to be more flexible to design and are more efficient than multimode cavities.^21,26^ Single-mode cavities are well suited for various applications such as sterilization, pasteurization, extraction, material synthesis and processing.^56^

#### 3.6.2 Multimode Cavity

In their simplest form, multimode applicators are a metal box much larger than the microwave wavelength. They are excited at a frequency well beyond their fundamental cutoff frequency. Unlike single-mode applicators, which are designed based on mathematical solutions to electromagnetic field equations, the design for multimode applicators is often based on intuition, experience, and trial and error. They are commonly employed in domestic microwave ovens and are also used for microwave chemistry. As the size of the applicator increases, the number of possible resonant modes also increases. To achieve a relatively uniform electric field inside the cavity, it is crucial to excite as many of these modes as possible. This helps to minimize nonuniform heating within the microwave oven.

Figure 3d shows a multimode cavity. The microwaves are distributed throughout the cavity using mode stirrers which are strategically placed to create a complex electromagnetic field distribution with multiple hot spots, unlike single-mode cavities. In domestic microwave ovens, however, the food (sample) is rotated on a turntable instead of using mode stirrers to achieve more uniform heating.

However, multimode ovens generally struggle to achieve uniform heating easily. This arises from the lack of predictability in how the parameters affecting uniformity vary with time. Therefore, various techniques are necessary to maintain consistent heating. These methods include metallic mode stirrers excite all possible modes, surface scanning to direct energy to all regions of interest, continuous movement of the material being heated, and hybrid methods that combine conventional and microwave heating. Multimode cavities are better suited for heating food and power applications such as material drying and plasma activation.^56,57^

To ensure high energy-transfer efficiency between the microwave source, cavity, and sample, proper impedance matching is essential. A mismatch leads to the reflection of microwave power, resulting in heating inefficiencies and unstable processing conditions.^26^ Because the dielectric properties of materials often evolve during a reaction, dynamic load tuning may be necessary to maintain optimal coupling throughout the process. Furthermore, the use of modeling and simulation facilitates the prediction of standing wave patterns and field distributions, allowing for the identification of local maxima and the optimization of sample placement. These factors are critical, as heating uniformity depends strongly on the complex interplay between cavity geometry, load size, permittivity, and frequency, variables that ultimately govern the material outcomes.

Modeling studies of a single-mode (TE10) cavity have confirmed that sample placement and load volume significantly influence the spatial distribution of the electric field and the resulting temperature profiles. When samples are placed centrally, the electric field and temperature gradients remain uniform. However, off-center placement causes the electric field distribution to become asymmetric, leading to a nonuniform temperature profile, a phenomenon attributed to localized energy deposition. Furthermore, the sample dimensions relative to the microwave penetration depth govern the standing wave interactions.^58^ Improper matching or placement can result in undesirable conditions, such as localized hotspots, nonuniform reaction progression, and microstructural heterogeneity; conversely, a well-designed applicator ensures improved reproducibility and thermal uniformity.^59^

Unlike single-mode cavities, multimode cavities exhibit complex and time-varying electromagnetic field distributions due to the superposition of multiple resonant modes and the operation of mode stirrers. Numerical studies have demonstrated that altering the mode configuration produces significantly different electric field distributions, indicating that the field is heavily dependent on instantaneous boundary conditions. These variations influence energy absorption and heating behavior, often resulting in localized regions of elevated temperature, with preferential heating frequently observed at sample edges. Although mode stirrers are employed to improve field uniformity, research indicates that spatial nonuniformities in energy deposition can still persist, leading to variability in heating profiles and affecting reaction progression within the material.^60^

These observations demonstrate that mode patterns, standing wave distributions, and sample placement directly govern the spatial distribution of electromagnetic fields within the cavity, thereby controlling local energy absorption and the resulting thermal gradients during microwave processing. This relationship is fundamentally described through electromagnetic field theory, where the volumetric power absorption is proportional to the square of the electric field magnitude (|E|^2^), and modulated by the dielectric properties of the material.^61^ Common drawbacks in microwave processing, such as improper impedance matching, lack of optimal sample placement, and variations in mode stirrer configurations, can lead to nonuniform heating and poor reproducibility.

#### 3.6.3 Multiphysics Simulations

COMSOL-based multiphysics simulations have demonstrated that cavity geometry, waveguide configuration, and material volume strongly influence electric field distribution and the resulting temperature profiles. This highlights the critical role of field localization in determining heating uniformity.^62^ Based on the eigenmodes of the cavity, the spatial distributions of the electric and magnetic fields are governed by boundary conditions and mode structures. This provides a framework for understanding the separation of transverse electric and transverse magnetic field contributions within the cavity.^63^

Experimental and numerical studies further confirm that electric and magnetic fields exhibit distinct spatial distributions within resonant cavities. In single-mode systems operating in TE102 and TE103 modes, these field maxima can be spatially separated, allowing the selective positioning of the samples in either electric- or magnetic-field-dominated regions. This allows for a direct comparison of isolated field-specific interactions. Here, dielectric materials predominantly couple with the electric field, while conductive or magnetic materials exhibit stronger interaction with the magnetic field, leading to distinct heating behavior (Figure 4a–c).^64^ Simulated studies have also reported the formation and evolution of localized hotspots due to nonuniform electromagnetic field distribution. As the temperature increases, the resulting rise in dielectric loss enhances local energy absorption, leading to amplified thermal gradients.

**Figure 4:**
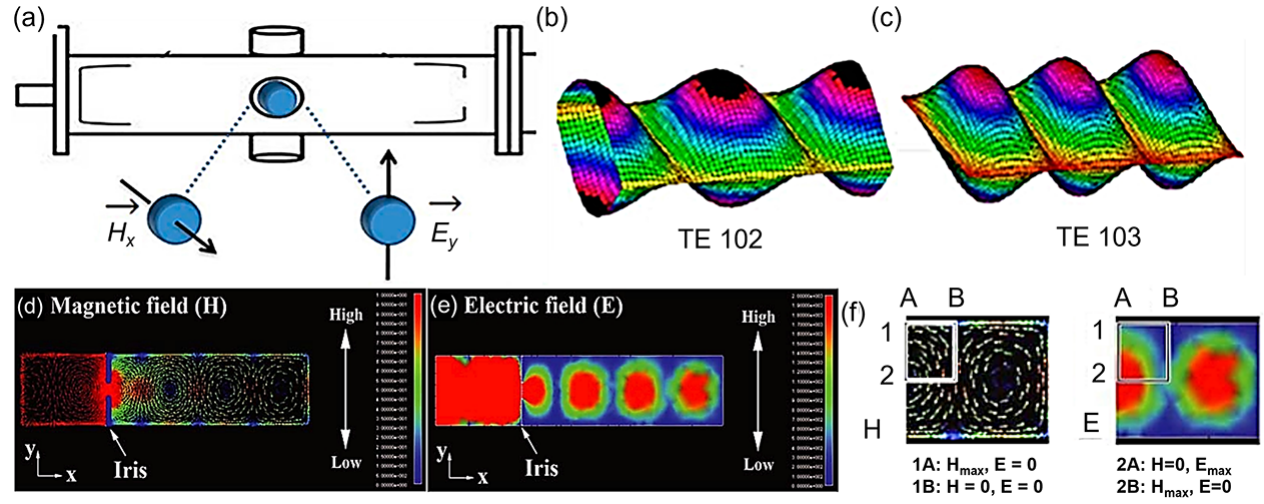
(a) Schematic diagram showing the orientation of electric (E) and magnetic (H) fields in a single-mode microwave cavity. (b) Amplitude distribution of the H-field component in TE102 mode (H-field maximum). (c) Amplitude distribution of the E-field component in TE103 mode (E-field maximum), illustrating the spatial separation of field maxima in a single-mode cavity. Adapted with permission^64^ under CC BY license. Simulated (d) H- and (e) E-field distributions in a single-mode microwave applicator, with (f) indicating positions corresponding to E- and H-field maxima, demonstrating spatial separation of field regions, reproduced courtesy of The Electromagnetics Academy.^65^

This distinction can be quantitatively described through volumetric power absorption, which depends on both the electric (E) and magnetic (H) field contributions. In single-mode cavities, electromagnetic wave simulations demonstrated that distinct spatial distributions of E and H fields can be achieved, with well-defined regions of electric and magnetic field maxima and minima.^65,66^ These regions allow for selective placement of samples in either the E-dominant or H-dominant regions, providing a controlled framework for probing field-specific interactions. The magnetic field distribution in Figure 4d shows localized regions of H-field maxima along the waveguide, while Figure 4e illustrates the corresponding E-field distribution with distinct maxima at different spatial locations. Figure 4f highlights the specific positions within the cavity corresponding to these field maxima, indicating potential sample placement zones for isolating E- and H-field effects.

In most material systems, microwave heating is predominantly driven by dielectric losses associated with the E-field. This is primarily due to the weak coupling of nonmagnetic materials with the H-field. However, in specific systems, such as magnetic oxides, placing the sample at a H-field maximum mediates energy absorption and triggers unique field-material interactions, highlighting a distinct nondielectric mechanism.^67^

### 3.7 Wave and Temperature Monitoring

Accurate monitoring systems are crucial during microwave processing to ensure uniform energy distribution, protect equipment from damage, and allow for precise control over processing conditions. Real-time feedback on wave behavior and temperature is essential for optimizing both the process and safety. Typically, microwave systems are equipped with both wave and temperature monitoring systems. Both transmitted and reflected microwaves are tracked to evaluate energy absorption efficiency. Radiofrequency (RF) detectors are used to convert microwave signals into direct current (DC) outputs which are monitored via oscilloscopes.

Accurate temperature determination in microwave systems remains a challenge due to nonuniform electromagnetic fields and localized heating effects. For temperature monitoring, noncontact pyrometers are commonly used. They are placed behind a window near the sample to provide accurate, real-time measurements. However, different measurement techniques can yield varying results depending on emissivity, spatial resolution, and probe-field interactions. For instance, thermocouples can perturb the electromagnetic field and introduce localized heating due to self-heating effects or by acting as antennas that emit or absorb microwave energy,^26^ while infrared methods may not represent the true material temperatures due to variations in emissivity.^68,69^ There are also reports of discrepancies between measurement techniques, which lead to differences in the observed temperature profiles and interpretation of microwave processing behavior.^70^ In addition, inaccurate temperature measurements can lead to a misinterpretation of the apparent nonthermal effects.^71^ Though useful for estimating the overall heat generation, calorimetric approaches provide bulk heating measurements and fail to resolve the localized temperature variations that are inherent to microwave heating.^72,73^ Some of the commonly used temperature measurement techniques are provided in Table 3, summarizing their operating principles, advantages, and limitations in microwave environments.

**Table 3:** Comparison of Temperature Measurement Techniques Used in Microwave Processing Systems

Technique	Operating Principle	Strengths	Failure Modes	Ref
IR Pyrometry or Thermography	Temperature from emitted infrared radiation from blackbody	Noncontact measurement; no direct probe–sample interaction; applicable in microwave environments	Strong dependence on emissivity; errors due to emissivity variation and nonuniform heating; may not reflect true material temperature	^68–70^
Fiber-Optic Probes	Optical temperature sensing based on band gap/luminescence change	Immune to electromagnetic interference; direct internal measurement	Single-point measurement; may miss gradients; fragile/slower within protective sheath	^71^
Indirect Calorimetry	Heat estimated from temperature rise of surrounding medium via energy balance	Can isolate and estimate overall heat generation	Indirect; heat-transfer losses unavoidable; inconvenient setup; cannot resolve local hot spots or true local temperature	^72,73^
Shielded Thermocouples	Voltage generation from thermal gradients	Simple, low-cost; used widely in conventional systems	Perturbs electromagnetic field, self-heating, errors due to thermal conduction, arcing can cause measurement error, behaves as antenna for MW	^68^

### 3.8 Plasma

Plasma, as the fourth fundamental state of matter, is composed of positively and negatively charged ionized gases. It is created by exposing a gas to a strong electromagnetic field or by heating it. Due to the presence of numerous charged particles, plasma is electrically conductive.

The density of ions, i.e., the number of ions per unit volume, quantifies plasma. Depending on the plasma density, microwaves can be absorbed, transmitted, or reflected by plasma.^40,51^ Microwave-generated plasma can be produced in both single-mode and multimodal cavities. Compared to the radiofrequency (RF) plasma, used in microelectronic processing, microwave plasma does not require electrodes to be generated. A smaller DC bias and a higher degree of ionization are observed in microwave plasma compared to RF plasma.^40,74^ Microwave plasma can be used for surface modification, sintering, processing, and film deposition. It can be produced in a closed chamber at low or high gas pressure, or in an open chamber under a flowing gas. Various gases can generate plasma, but the most common ones are argon, helium, nitrogen, oxygen, and hydrogen.^40^

## 4 Interaction of Microwave with Materials and Their Mechanisms

The interaction of microwaves is highly dependent on materials’ intrinsic properties, particularly electrical conductivity and dielectric characteristics. A comprehensive understanding of interaction mechanisms is essential for the advancement of microwave processing aimed at developing innovative materials and structures. These mechanisms dictate how microwave energy is absorbed, dissipated, or reflected depending on the materials’ properties.

### 4.1 Microwave Losses

Microwave losses refer to mechanisms that convert microwave electromagnetic energy into heat or electric discharge, thereby reducing the overall transmitted power. The specific mechanism of loss depends on how different materials interact with alternating electromagnetic fields (EMFs) at microwave frequencies, which range from 300 MHz to 300 GHz.^34^ These interactions can be broadly divided into the following six distinct categories.

#### 4.1.1 Dipolar Loss

In this mechanism, polar molecules, such as water, attempt to align with the alternating electric field. However, at microwave frequency, these molecules cannot keep pace with the rapidly alternating field, which causes a phase lag in their dipoles. These lagging dipoles dissipate energy as heat, known as dipolar loss. This is a very common mechanism and is a widely known mechanism in microwave heating. For example, it is the primary mechanism for heating most food products and any other material containing molecular dipoles.^26,75^

#### 4.1.2 Ohmic Loss or Conduction Loss

In this mechanism, free electrons within the material move under the influence of electric field, generating an electric current (I). Depending on the ohmic resistance (R) of the material, this current produces Joule heating (=RI^2^). Ohmic loss primarily occurs in conductive materials such as metals, alloys, and highly doped (degenerate) semiconductors, resulting in both reflection and dissipation of microwave energy.^76^

#### 4.1.3 Ionic Loss

In this mechanism, mobile ions (either dissolved in a material or within an ionic crystal) oscillate in an alternating electric field. Similar to the dipolar loss, these ions cannot keep pace with the high-frequency alternating field. This causes ionic displacement and frictional resistance, which dissipates energy as heat within the ionic material.

Ionic compounds are made of cations and anions held together by ionic bonds, typically having high melting points and brittle structures. At room temperature, many ionic compounds, such as sodium chloride (NaCl), barium titanate (BaTiO_3_), and lead zirconate titanate (PZT), have low dielectric loss and are poor absorbers of microwave. While pure solid ionic compounds usually exhibit low microwave absorption at room temperature, factors like defects, doped conductive phases, polarizable structures, and being in a molten or ionically conductive state significantly increase their microwave coupling. At higher temperatures, ions become more mobile and generate ionic conductivity, leading to increased microwave heating and potentially thermal runaway. Therefore, materials exhibiting ionic behavior can be preheated to a specific temperature, either conventionally or by doping them with microwave absorbers, before being processed with microwave.^26^

Consequently, ionic compounds may not be directly affected by microwaves initially. Instead, defective structures, microwave absorber particles, or even the container itself can absorb microwave energy and raise the overall temperature. Then, at specific temperature, the ionic compound rapidly absorbs the microwave, leading to a significant temperature increase.

Thermal runaway is a self-accelerating process where the temperature of a material rapidly and uncontrollably escalates under microwave radiation. This phenomenon occurs when suddenly the materials (such as ionic compounds) capability to absorb microwave energy suddenly increases. This positive feedback loop must be carefully controlled to prevent hot spots, melting, material breakdown, or equipment damage. However, thermal runaway can be intentionally managed to enable the sintering of high-temperature materials, the joining of dissimilar high-temperature materials, and the enhancement of certain reactions. For example, alumina (Al_2_O_3_) does not absorb microwaves at room temperature, but preheating it to around 300–500 °C can alter its dielectric loss, leading to rapid heating and the possibility of thermal runaway.^34,77^

#### 4.1.4 Interfacial Polarization Loss

This mechanism occurs in heterogeneous materials, like composites. It happens when charged carriers (such as electrons, holes, ions) become trapped and accumulate at the phase boundaries or interfaces between materials with different dielectric properties. These charged carriers move until they hit a boundary and cannot quickly respond to the alternating EMF, which results in heat generation. Porous materials, as well as single- and multiphase composites (like polymer matrix ceramic composites) are particularly susceptible to interfacial polarization loss. This mechanism is ideal for the microwave heating of composites and the sintering of multiphase and ceramic composites.^78^

The total dielectric loss (*ε*″) is a combination of dipolar loss $\left(\varepsilon_{dipolar}^{\prime \prime }\right)$ coming from dipole reorientations, electronic loss $\left(\varepsilon_{electronic}^{\prime \prime }\right)$ coming from electron movement, ionic loss $\left(\varepsilon_{ionic}^{\prime \prime }\right)$ coming from ionic displacement, and interfacial polarization loss $\left(\varepsilon_{interfacial}^{\prime \prime }\right)$ coming from trapped charge carriers. It can be written as

8 $$\varepsilon^{\prime \prime }=\varepsilon_{dipolar}^{\prime \prime }+\varepsilon_{electronic}^{\prime \prime }+\varepsilon_{ionic}^{\prime \prime }+\varepsilon_{interfacial}^{\prime \prime }$$

Any other sources of dielectric loss can be added into eq 8.

#### 4.1.5 Magnetic Loss or Hysteresis Loss

In this mechanism, magnetic domains within a material, such as ferrites, try to align with the microwave alternating magnetic field. The internal motion and friction of these domain walls cause a response lag, leading to microwave energy dissipating as heat. This magnetic response lag creates a hysteresis loop in the magnetic flux density (B) versus magnetizing force (H) curve. The larger this loop, the greater the energy loss. Magnetic loss is an ideal mechanism for microwave sintering and selective heating of magnetic materials.^27^ The magnetic loss per unit time and volume (*Pμ*) is given by

9 $$P_{\mu }=\frac{1}{2}\omega \mu_{0}\mu_{r}^{\prime \prime }H_{0}^{2}$$

where ω is the angular frequency, μ_0_ is the permeability of free space, μ_*r*_ is the relative permeability of the material and H_0_ is the magnetic field strength.^26^

#### 4.1.6 Resonance Loss

In this mechanism, internal components of a material (like atoms, molecules, ions, electrons, or magnetic domains) oscillate with maximum amplitude under a high frequency alternating EMF. This occurs when the microwave frequency matches the natural resonance frequency of the material. At this specific frequency, microwave energy is absorbed efficiently, generating heat all around the material. Of all the mechanisms, resonance loss is the least significant in the solid-state microwave processing of materials.^27^

### 4.2 Microwave Heating Effects

Microwave heating is fundamentally different from conventional heating methods in both the mechanism of heating and the resulting outcomes. The different characteristics of conventional and microwave-based heating are listed in Table 4. Conventional thermal processes rely mainly on conduction and convection. This leads to slow, surface-dominated, and nonselective heating. In contrast, microwave radiation directly propagates energy into the material through EMFs, enabling rapid and volumetric heating that is highly dependent on the dielectric and magnetic properties of the materials. This energy deposition occurs at the molecular level, mainly through dipolar polarization and ionic conduction mechanisms, resulting in selective and localized heating. Figure 5a–c represents a comparison between conventional heating and volumetric heating in microwaves. Volumetric heating heats the sample homogeneously, enabling an efficient heating of large samples with a more “uniform thermal history” throughout the sample.^79^ This ensures that the reaction proceeds uniformly throughout the samples, reaching the state of completion at the same time.^80^

**Table 4:** Comparison of Microwave Heating and Conventional Heating characteristics^81^

Microwave Heating	Conventional Heating
Energetic coupling	Conduction/convection
Coupling at the molecular level	Superficial heating
Rapid	Slow
Volumetric	Superficial
Selective	Nonselective
Dependent on the properties of the material	Less dependent

**Figure 5:**
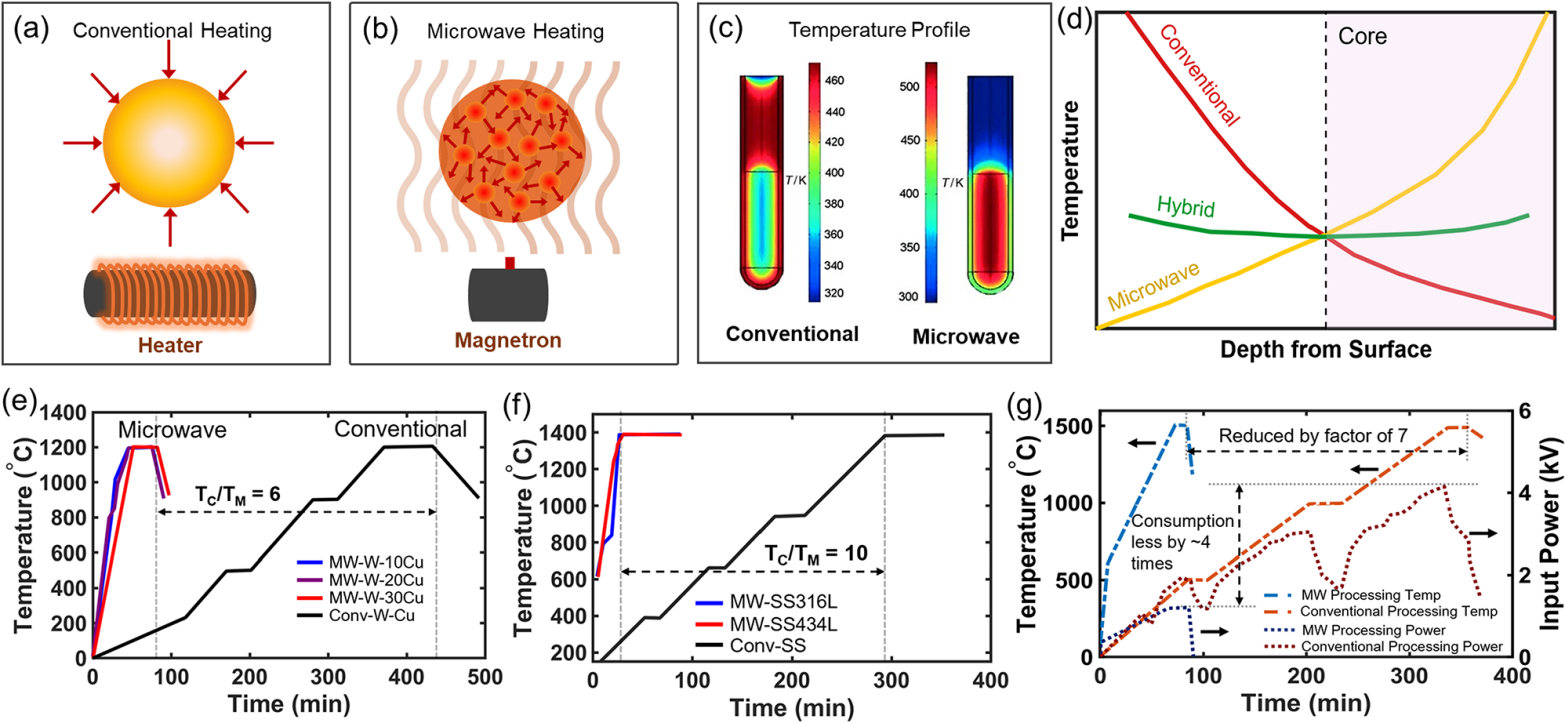
Comparison of the heating mechanisms of (a) conventional and (b) microwave heating. In conventional heating, the outer surface of the sample is heated first, and then the heat diffuse inward through conduction. In contrast, microwave heats the sample volumetrically. (c) Temperature profiles of conventional and microwave heating. Reprinted with permission.^80^ (d) Comparison of depth of heating between conventional, microwave, and hybrid heating. Temperature versus time profiles of conventional versus microwave heating of (e) W–Cu composites, and (f) stainless steel. (g) Power consumption and heating rates of conventional and microwave sintering. Adapted with permission.^82^ Copyright 2014 Taylor & Francis; adapted with permission^83^ under CC BY license; adapted with permission.^84^ Copyright 2006 Elsevier; and adapted with permission.^85^ Copyright 2007 Elsevier.

Figure 5d illustrates the temperature versus depth profiles of conventional, microwave, and hybrid heating. In conventional methods, the surface temperature is high, but the temperature rapidly decreases as the sample depth increases. Conversely, in microwave heating, the maximum temperature is achieved deep within the sample, with the temperature decreasing as it approaches the surface. Hybrid heating approach is capable of maintaining a uniform temperature from the sample surface to its depth. Figure 5e and f compare the temperature versus time consumption profiles for WC-Cu and Stainless-Steel samples. The use of microwave processing significantly reduces the processing time by 6 times for the WC-Cu samples and by 10 times for the Stainless-Steel samples compared to conventional methods. Figure 5g shows the power consumption and heating times for conventional and microwave heating, revealing that using microwave processing resulted in substantial savings in both power and processing time, with reduction factors of 7 and 4, respectively, compared to conventional heating.

Table 5 compares various parameters of microwave processing with six other heating and sintering techniques, including conventional heating, self-propagating high-temperature synthesis (SHS), Joule heating, induction heating, spark plasma sintering (SPS), and laser processing. Microwave processing exhibits several unique characteristics, particularly in its mode of energy coupling and volumetric behavior. These distinct characteristics are often attributed to thermal and nonthermal effects. The following sections examine these two classes of effects in greater detail, highlighting how microwave-material interactions differ from those observed under conventional heating.

**Table 5:** Comparison of Different Parameters across Conventional Heating,^86^ SHS,^87–89^ Joule Heating,^90,91^ Induction Heating,^92^ Microwave Heating,^26,36^ Spark Plasma Sintering,^36,93^ and Laser Processing^a^ ^94,95^

Parameter	Conventional	SHS	Joule Heating	Induction Heating	Microwave Heating	SPS	Laser Processing
Heating Mechanism	Initiated by external furnace heating, reactions occur via solid-state diffusion	Exothermic reaction ignition followed by self-sustained combustion	Direct resistive heating through electric current flow	Eddy current heating induced by alternating EMF	EMF coupling (dipolar polarization, ionic conduction, magnetic losses)	Joule heating via pulsed DC current through the die and under pressure	Photon absorption and photothermal conversion
Heating Rate	Slow to moderate, limited by poor diffusion kinetics	Extremely rapid due to exothermic reaction kinetics	Rapid to extremely rapid	Rapid and controllable	Rapid to extremely rapid, controllable, material-dependent	Very rapid, controlled via pulsed DC current and applied pressure	Extremely rapid, localized, energy-density dependent
Penetration Depth	Not limited, depends on kinetics of materials,	Not limited	Not limited	Limited, dependent on frequency, σ, and permeability	Limited, dependent on temperature, frequency, and material properties	Not limited	Limited, dependent on energy density and material properties
Heating Profile	External heating with inward conduction and propagated through diffusion	Localized reaction with distinct reaction, and cooling zones; rapid temperature rise	Bulk but current-pathway dependent; localized at barriers and hotspots	Surface-dominated heating with inward conduction	Volumetric and selective; nonuniform due to heterogeneity and hotspots	Bulk heating with evolving current paths causing local temperature gradients	Highly localized, surface-dominated, asymmetric along scan direction
Densification mechanism	Thermally driven diffusion in grain boundary, surface and lattice transport	Self-driven synthesis often leads to porous or partially densified structures	Thermally driven diffusion via resistive heating, with rapid densification possible under fast heating	Thermally driven diffusion with localized heating at particle contacts enhancing transport	Diffusion-driven with potential field-assisted and nonequilibrium effects	Pressure-assisted densification via diffusion and plastic deformation under pulsed current	Melt pool flow, bubble escape/re-entrapment, and remelting densification
Interfacial phases/reactions	Equilibrium phases through interfacial reactions and diffusion-controlled product layer growth	Rapid nonequilibrium phase formation with limited diffusion; metastable phases and heterogeneity	Primarily temperature-driven phase evolution; localized overheating can influence reactions	Predominantly equilibrium phase formation; localized heating can enhance interfacial diffusion	Selective and localized reactions; potential nonequilibrium phase formation	Controlled interfacial evolution with possible current-enhanced diffusion	Rapid melting/solidification; strong metallurgical bonding with nonequilibrium phase formation
Geometrical constraints	Governed by particle contact, diffusion distance and packing homogeneity	Limited to compacted powders or simple geometries that can sustain combustion	Relies on conductive pathways; sensitive to current, geometry, and hotspots	Dependent on σ, part geometry, and coil design, complex shapes are challenging	Dependent on field distribution, cavity design, and materials coupling	Restricted by die geometry, applied pressure, and temperature gradients	Line-of-sight, layer-wise process governed by scan strategy and melt pool
Common Application Domains	Bulk ceramics, multicomponent oxides, alloys, inorganic materials	Powders, intermetallics, ceramics, composites	Metals, alloys, and conductive materials, energy materials, chemical processing	Metals, alloys, conductive materials, powder metallurgy components, sintering	Ceramics (oxides, carbides, nitrides), intermetallics, and chalcogenides	Ceramics, intermetallics, nanostructured materials	Metals, alloys, composites, additive manufacturing components

a SHS: Self-propagating high-temperature synthesis; SPS: Spark plasma sintering; EMF: Electromagnetic field; DC: Direct current; σ: Electrical conductivity.

#### 4.2.1 Thermal Effects

Microwave-induced thermal effects result from direct temperature elevation. They arise from the ability of the electromagnetic fields to couple with materials and generate heat, which leads to phenomena such as superheating, hot spot formations and selective heating. Understanding these phenomena is essential for optimizing microwave processing across different material systems.

##### 4.2.1.1 Superheating

One of the thermal effects of microwave irradiation is superheating, as illustrated in Figure 6 a. This phenomenon occurs mainly in liquids when they reach temperatures roughly 13–26 °C above their normal boiling temperature without actually boiling. This is due to an “inverted heat transfer” effect, where the interior of the liquid heats up faster than its surface, which is the opposite of conventional heating where thermal conduction occurs from the outside.^96^ The origin of this effect is the volumetric nature of microwave absorption. Superheating is particularly common in polar solvents like water, ethanol, or dimethylformamide (DMF), which have high dielectric losses and couple efficiently with oscillating electromagnetic fields.

**Figure 6:**
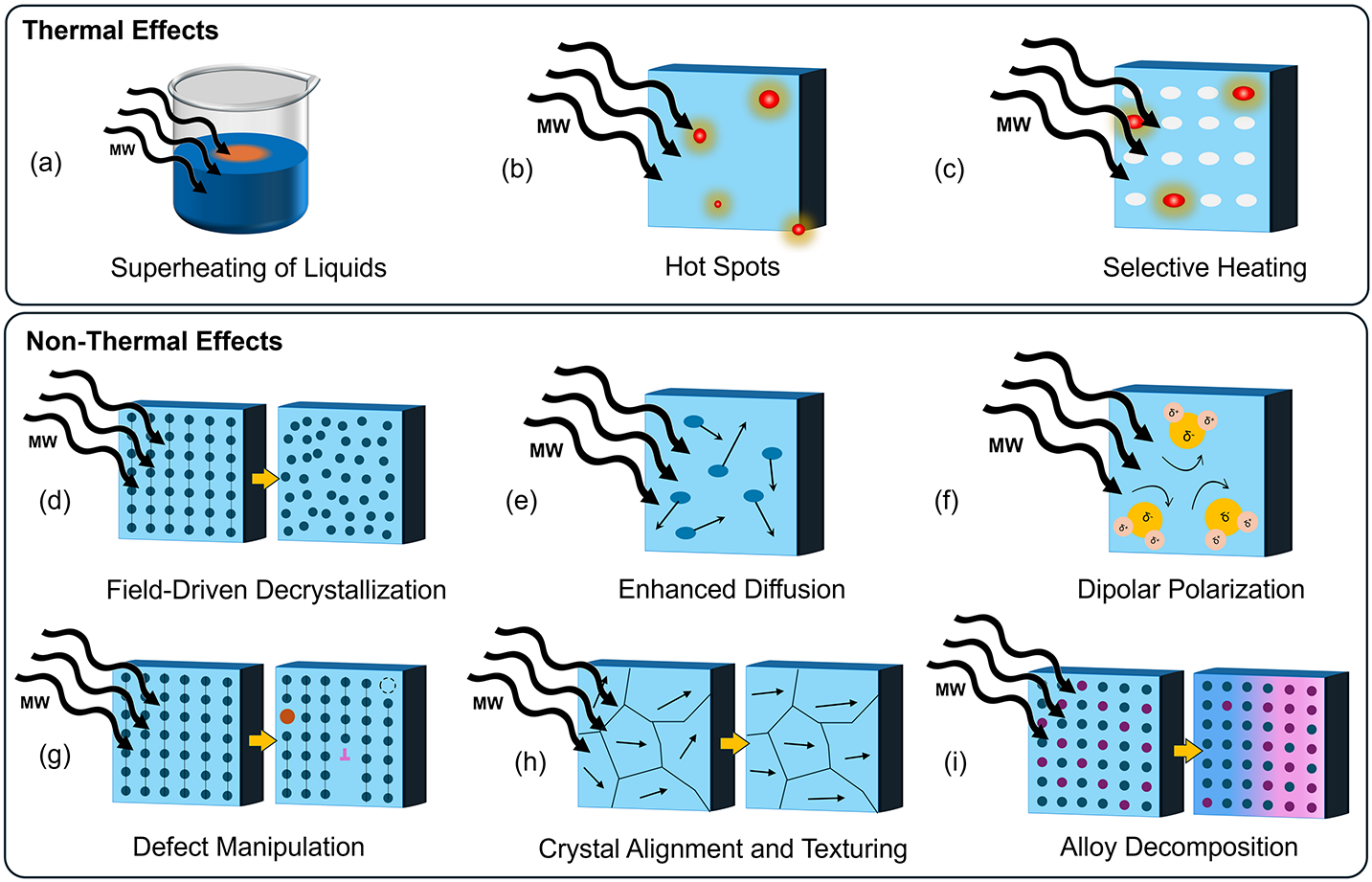
Microwave thermal and nonthermal effects. Thermal effects include (a) superheating (primarily in liquids), (b) formation of hot spots, and (c) selective heating. Nonthermal effects include (d) field-driven decrystallization, (e) enhanced diffusion, (f) dipolar polarization, (g) structural defect manipulation, (h) crystal alignment, and (i) alloy decomposition.

As mentioned previously, dipolar molecules realign with the EMF, and this rotational friction creates localized heating.^26^ In the absence of nucleation sites (such as rough vessel walls or dissolved gases), which can occur in microwave-transparent containers, the initiation of bubble formation is delayed. This allows the temperature to rise significantly beyond the normal boiling point. Additionally, the lack of a temperature gradient along the vessel walls and the suppression of convention under the EMF trap thermal energy in the core of the liquid, creating a metastable, superheated state. This behavior has been well-documented in microwave-assisted organic reactions. For instance, the study on the dehydration of xylose to furfural showed that microwave heating at 200 °C resulted in a reaction rate constant of 197 min^–1^, compared to just 13.9 min^–1^ with conventional heating. Kinetic modeling attributed this increase not to a global temperature rise, but to the presence of minor reaction zones experiencing higher localized temperatures.^97^

Microwave-induced solvent superheating has also been observed in unstirred alcohols. For instance, they reached bulk temperatures higher than their boiling point at 75 W. Similarly, methanol, ethanol, and isopropyl alcohol reached temperatures 14 °C, 21 °C, and 28 °C higher than their boiling points.^98^ Another example demonstrating superheating is observed in microwave-responsive carbon catalysts. High yields (80 mol %) of 5-hydroxymethylfurfural was achieved using pulsed microwave heating at 160 °C within 4 cycles, a significant improvement compared to continuous heating. This high efficiency was attributed to superheating of microwave-responsive catalysts (MRCs).^99^ Therefore, superheating can be used as a strategic tool in synthesis of materials with careful thermal and pressure management.

##### 4.2.1.2 Hot Spots

Another thermal effect of microwave irradiation is the formation of hot spots, which are localized regions where the temperature is significantly higher than the surrounding areas. These regions create a temperature gradient within the material during microwave exposure.^100^ Figure 6b shows representation of hot spots. This phenomenon is suggested to be a result of inhomogeneities in the applied electromagnetic field, causing certain zones of the sample to reach much higher temperatures than the average macroscopic temperature. Specifically, materials with a higher dielectric loss tend to absorb more microwave energy, heating more rapidly than their surroundings, and creating localized thermal gradients. It is estimated that a temperature difference of around 100–200 °C can exist between the hot spot and the surrounding material. The size of these hot spots varies with material properties. Larger particles can produce bigger hot spots, and more microwave-absorbing materials form more prominent ones. Hot spots can also form due to differences in the dielectric properties of a material, an irregular distribution of electromagnetic field strength, or as a consequence of volumetric dielectric heating.^81^

The formation of hot spots can also be explained by the interaction of electromagnetic waves with discontinuous or sharp interfaces within a material.^34^ At these points, localized enhancement of the electric field can occur due to abrupt changes in dielectric or magnetic permeability, which can exponentially increase local heating. This effect is further amplified by feedback mechanisms, as an increase in temperature results in higher dielectric loss; thus, in turn increases the microwave absorption, and further raises the local temperature.^101^ Therefore, hotspots are an intrinsic and often unavoidable aspect of microwave processing.

These localized hot spots can significantly influence microstructural evolution by generating temperature gradients within a material. This leads to localized phase transformations, defect formation, and crack development, resulting in microstructural heterogeneity. Such nonuniformity can affect reproducibility and complicate the interpretation of the observed property enhancements. Therefore, distinguishing controlled selective heating from uncontrolled heterogeneity remains challenging and requires spatially resolved characterization of both temperature and microstructure.^102^

While these hot spots present challenges in process control and stability, they can also be strategically utilized to accelerate reactions during synthesis or even used as a flash melting technique. Such benefits are not easily achievable with conventional processes.

##### 4.2.1.3 Selective Heating

Selective heating refers to the preferential heating of certain components in a mixture when exposed to microwave radiation. Since different materials absorb microwave energy at different rates, microwave frequencies can be selectively chosen to heat only the desired parts of the materials,^38^ as illustrated in Figure 6c. This phenomenon arises primarily from differences in dielectric properties of various components, driven mainly by dielectric constant (ε′) and dielectric loss (ε″).^103^

For example, microwave processing has been used to synthesize and crystallize thin-film titanium dioxide (TiO_2_) on indium tin oxide (ITO) substrates.^104,105^ The microwave selectively heated the conductive ITO layer, which then enabled the heterogeneous nucleation and crystallization of the TiO_2_ from a liquid precursor onto the substrate. This demonstrated that the anatase phase could be stabilized at low temperature (150–160 °C) by localized field absorption using a conducting layer. One theory suggests that this process enhances the local field strength, which distorts the atomic structure locally and provides some additional nucleation sites for the formation of the anatase phase.^24^ The precise and localized heating allows for spatial control over the synthesis process.

Similarly, researchers have reported the rapid formation of metal chalcogenides using microwave. In this process, metal powders such as Fe, Mn, Ta, Cr, and Sn coupled efficiently with the microwave field, reaching temperatures between 800 and 1000 °C in minutes, significantly faster than the surrounding chalcogenides.^106^ These examples highlight how the contrast in the dielectric properties of materials can induce selective heating, enabling unique reaction pathways, rapid kinetics, and efficient processing routes that are difficult to achieve with conventional methods.

#### 4.2.2 Nonthermal Effects

When synthesizing materials using microwaves, certain effects are observed that cannot be explained by conventional heating methods. This opens a new area of discussion regarding effects that are potentially field-driven and nonthermal in nature. Under the effects of an applied electromagnetic field, materials can undergo changes at both atomistic and microstructural levels.^24,81^ In this condition, in addition to thermal effects, the material can show one or a combination of nonthermal effects.

##### 4.2.2.1 Field-Induced Decrystallization

Field induced decrystallization is a nonthermal effect where a crystalline solid undergoes a loss of crystallinity under the exposure of strong and oscillating electromagnetic field. Unlike conventional thermal heating, which typically promote crystallization or grain growth, microwave radiation has been found to induce atomic disorder, defect generation, and even amorphization in crystalline materials under certain conditions.^107^ This effect is illustrated in Figure 6d.

One such example of field-induced decrystallization is recorded in the anisotropic expansion observed in ceramic crystals (Figure 7).^108^ This was observed in the in situ synchrotron studies during microwave (MW) exposure, where a sudden drop in the intensity in diffraction is observed, demonstrating a partial decrystallization (Figure 7a and b). XPS studies demonstrate that a new high-binding energy O 1s component demonstrates the presence of oxygen vacancies with no change in the Ti 2p component (Figure 7c and d). Raman spectra also shows blue-shifts indicating the existence of lattice strain in the structure of MW synthesized samples (Figure 7e). These studies effectively demonstrate a nonthermal, defect-mediated mechanism.

**Figure 7:**
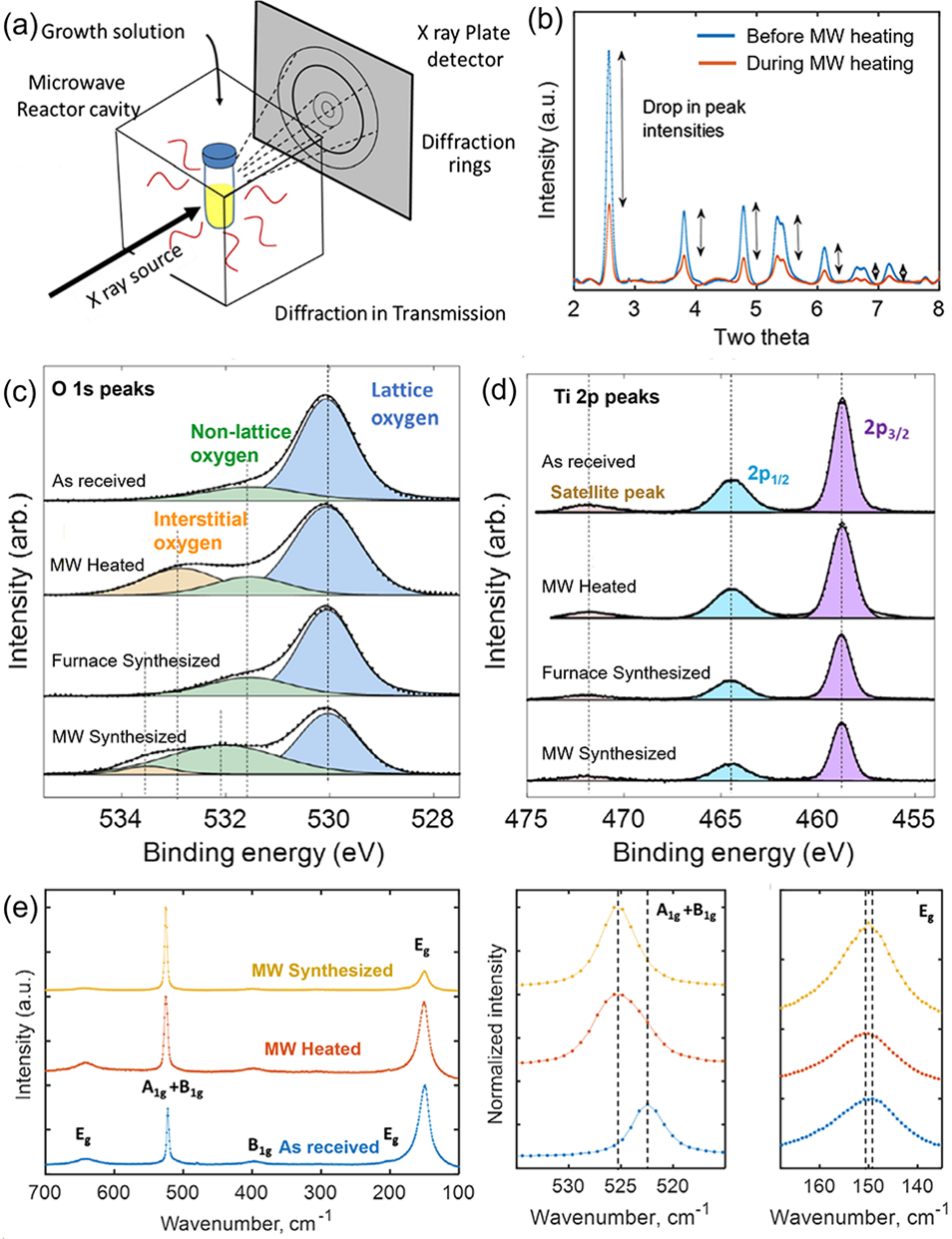
In situ evidence of field-driven changes in anatase TiO_2_ under microwave irradiation. (a) Schematic picture of in situ diffraction setup and processing. (b) The X-ray diffraction (XRD) patterns before microwave (MW) and during MW heating show a systematic decrease in peak intensities, indicative of partial decrystallization. (c) and (d) High-resolution X-ray photoelectron spectroscopy (XPS) of O 1s and Ti 2p for as received anatase, MW heated, furnace synthesized, and MW synthesized powders. (e) Raman spectra for as received, MW heated, and MW synthesized powders showing the six anatase modes (700–100 cm^–1^) and blue-shifts in A_1g_ + B_1g_ (∼525 cm^–1^, ∼+3 cm^–1^) and E_g_ (∼150 cm^–1^) consistent with defect-induced lattice strain. Reproduced with permission.^24^ Copyright 2021 Springer Nature; and with permission.^108^ Copyright 2019 John Wiley and Sons.

This effect was rigorously investigated by Nozariasbmarzet al., who demonstrated that single-crystal silicon wafers exposed to microwaves in a single-mode cavity exhibited significant decrystallization throughout the bulk of the material, not just at the surface.^109^ Transmission electron microscopy (TEM) and selected area electron diffraction (SAED) patterns confirmed this observation (Figure 8a and b). While pristine silicon showed sharp, well-defined diffraction spots characteristic of a single crystal, postmicrowave samples revealed concentric diffraction rings, which are typical of nanocrystalline or amorphous structures (Figure 8b). The micrographs showed nanoscale domains (∼5 nm) embedded within an amorphous matrix, clear evidence of microwave-induced structural degradation. The X-ray diffraction pattern (XRD) spectra of the postmicrowave samples showed a dramatic reduction in intensity and peak broadening of the (400) reflection (Figure 8c), indicating a substantial breakdown in crystal periodicity. The microwave induced decrystallization resulted in a dramatic drop in the thermal conductivity of the samples which confirms that the phenomena is real and not a characterization artifact (Figure 8d).

**Figure 8:**
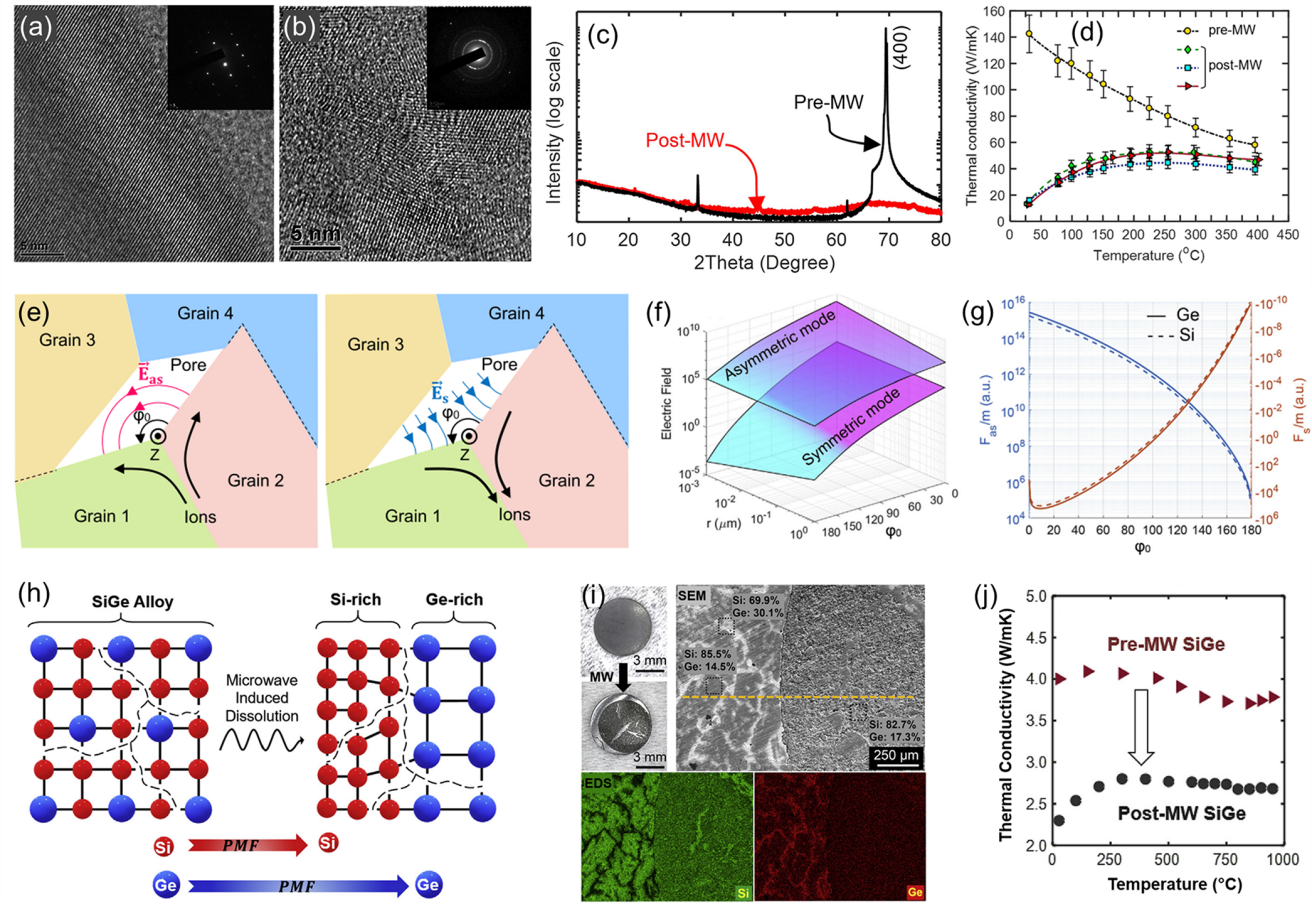
High resolution transmission electron microscopy (TEM) images and corresponding selected area diffraction (SAD) patterns of the crystal silicon wafer (a) before and (b) after microwave radiation. (c) X-ray diffraction (XRD) patterns of the silicon wafer before (pre-MW) and after (post-MW) microwave radiation. (d) Comparison of the thermal conductivity versus temperature of single crystal silicon wafers before (pre-MW) and after (post-MW) microwave radiation. Reproduced with permission.^109^ Copyright 2018 AIP Publishing. (e) Asymmetric and symmetric electric field amplitude modes near grain boundary interfaces. (f) Asymmetric and symmetric modes of the electric field amplitudes. Reproduced with permission.^110^ Copyright 2019 Elsevier. Decomposition of the SiGe alloy by microwave processing. (g) Ponderomotive forces (PMFs) per unit mass underwent by ions of Si and Gedue to the inhomogeneity of the field near the interfaces. (h) Schematic drawing of decomposition of Si and Geatoms under microwave radiation. (i) Macroscopic image, scanning electron microscopy (SEM) micrograph and energy dispersive spectroscopy (EDS) analysis of SiGe alloy before and after microwave processing. (j) Thermal conductivity versus temperature of SiGe alloy before and after microwave processing. Reproduced with permission.^110^ Copyright 2019 Elsevier.

Importantly, this transformation could not be attributed to temperature effects alone. In controlled experiments, half of the wafer was masked with an ∼10 μm metallic nickel layer, only the exposed portion underwent decrystallization, even though both regions experienced similar thermal conditions through conduction. This strongly suggested that the electromagnetic field itself played a central role, likely through field-driven atomic displacement, local strain accumulation, and defect generation mechanisms.^109^

In similar research, when Anatase (TiO_2_) was exposed to microwave, decrystallization was observed. The XRD patterns of microwave processed samples exhibited a bigger hump than conventional nano powders. This indicates a higher degree of amorphous phase in the microwave processed samples, demonstrating that the material became amorphous upon heating, rather than becoming more crystalline or undergoing a typical phase transformation.^108^

The underlying driving force for nonthermal effects such decrystallization is thought to be the ponderomotive force (PMF), as proposed by J. H. Booske et al.^111^ Ponderomotive forces are time-averaged forces experienced by charged species (e.g., electrons, ions) in spatially inhomogeneous, high-frequency electromagnetic fields. These forces induce local lattice instabilities and disrupt atomic ordering, even at moderate temperatures. The PMF per unit volume under electric field can be represented by eq 10:

10 $$f_{PMF}=-\frac{\omega_{p}^{2}}{\omega^{2}+\vartheta^{2}}\nabla \left(\frac{\varepsilon E^{2}}{2}\right)$$

where ω is the microwave frequency, ϑ is the collision frequency, *ε* is the dielectric constant, E is the amplitude of electric field of the microwave, and $\omega_{p}^{2}=\frac{nq^{2}}{m\varepsilon }$ is the plasma frequency.^110^ Here n is the concentration of charge species, q is the species charge, m is the mass of the particles, and *ε* is the dielectric constant of the medium.

Understanding the PMF requires numerical studies that consider the effects of both electric and magnetic field components. Birnboim et al. numerically showed that for a neck region formed between particles, the electric field is significantly elevated in the spherical contact domain between grains.^112^ This results in enhanced ion migration. As shown in Figure 8e and f, two distinct modes of interfacial electrical fields, asymmetric mode, which diverges as *r* → 0 and produces strong PMFs that push materials toward the interface, and the symmetric mode, that diminishes at the interface and generates weaker forces toward the bulk. The asymmetric mode dominates at small pore angles, which promotes densification and interfacial filling.^110^

##### 4.2.2.2 Enhanced Diffusion

One of the most intriguing effects observed during microwave processing is enhanced diffusion in solids under electromagnetic field exposure. Unlike conventional heating, where diffusion is driven solely by elevated temperatures, microwave heating enhances diffusion through a combination of volumetric heating, field-matter coupling, and dielectric polarization effects. As illustrated in Figure 6e, exposing certain materials to microwaves results in an increase in atomic or ionic mobility due to the direct interaction between the electromagnetic field and the material. This phenomenon has been observed in microwave synthesis, leading to enhanced sintering, accelerated grain growth, and an increased number of reaction sites.

A particularly illustrative example is the ion-exchange of potassium into sodium-aluminosilicate glass. During microwave processing, the diffusion of K^+^ ions increased by nearly three times compared to conventional heating. Since local temperature measurements could not fully account for these accelerated rates, it was concluded that the effect was not purely thermal. The enhancement was attributed to an increase in the correlation factor due to relaxation type loss mechanism, or a higher concentration of extrinsic defects.^113,114^

These results indicate that enhanced diffusion is a significant microwave phenomenon that enables faster ion exchange rates. This could reduce energy consumption and the overall heat required for a process to occur. It could also provide a valuable processing pathway for temperature-sensitive materials.

##### 4.2.2.3 Dipolar Polarization

Dipolar polarization is one of the fundamental processes by which microwave fields interact with materials, especially with dipole moments. When such materials are exposed to the continuously oscillating field, they attempt to realign with the field. This is demonstrated in Figure 6f. However, due to the inherent rotational inertia and interaction with surrounding lattice, they experience a phase lag. This lag results in frictional losses at the molecular level, and this energy is dissipated as heat, contributing to temperature rise in the material.^34,115^

Dipolar polarization can be observed in various materials systems that have dipole moments. Dielectric polarization of liquid and solid formic acid is an example. The liquid formic acid has a high dielectric constant (*ε*′ ≈ 56 at 25 °C). Even in the solid state, dielectric relaxation was observed at temperatures as low as −50 °C. In this system, the dipolar polarization of the molecular dipoles was thought to be the dominant mechanism explaining the microwave energy absorption through dipolar relaxation.^116^

Dielectric heating emerges as a fundamental mechanism whose contribution spans to both thermal and nonthermal effects, hence playing a pivotal role in understanding and optimizing the synthesis of materials using microwaves.

##### 4.2.2.4 Improved Chemical Reactivity

Microwave irradiation has been observed to enhance the chemical reactivity of reactions beyond what is predicted by conventional thermal routes. This often leads to faster kinetics, improved yields and modified selectivity of the reactions. These enhancements have been attributed to various nonthermal effects such as dipolar polarization, ionic conduction and selective heating, which promote the localization of energy and altered kinetic pathways.^117^

Among these, ionic conduction plays an important role in localized superheating. Dissolved ions oscillate under applied microwave field, colliding with surrounding species, generating localized heat more efficiently than dipolar rotation. Free ions in the reaction mixture orient in the direction of the applied electric field and cause instantaneous superheating.^117^

For example, metallophthalocyanines are commercially used as colorants in photodynamic therapy, organic semiconductors, and ink jet printing. Conventional synthesis methods typically need processing at 200 °C for several hours, while their purity is limited. Microwave synthesis enabled enhanced synthesis rates and efficient heating, leading to purer metallophthalocyanines in minutes.^118^

Another example of improved chemical reactivity can be observed in the Bernthsen reaction for synthesizing acridines. Acridines are biological compounds with a wide range of properties and medicinal applications, such as DNA intercalating agents, bactericides, anticarcinogenics, and antimalarials. The Bernthsen reaction typically requires heating a diarylamine with a carboxylic acid and a catalyst such as zinc chloride, to form 9-substituted acridines. Microwave irradiation of diphenylamine, zinc chloride and benzoic acid lead to the formation of 9-phenylactidine in a few minutes. Compared to classical methods, yields were higher, the reaction was faster, and the quantity of zinc chloride used could be reduced, which helps in protecting the environment.^119^

The kinetics in many studies were evaluated using the Arrhenius equation, in which the rate constant is expressed as *k* = *A* exp­(−*E*_*a*_/*RT*), where, *k* is the rate constant, *A* is the pre-exponential factor (or frequency factor), *E*_*a*_ is activation energy, *R* is gas constant, and *T* is absolute temperature. The activation energies are typically extracted as linear fits of ln*k* vs 1/*T*. Omran et al.^120^ reported a decrease in the apparent activation energy under microwave irradiation, while Binner et al.^121^ observed no change in activation energy, but reported an increase in the pre-exponential factor. These contrasting observations suggest that possibly different kinetic parameters may be affected based on the system and the experimental conditions. One critical issue in interpreting these results lies in the accuracy and comparability of temperature measurements. Most methods use bulk measuring techniques such as IR probes, where measurement of localized temperature gradients is challenging. Binner et al. argued that unrealistically large temperature deviations could account for the observed rate enhancements.^121^ Nevertheless, localized hot spots and transient thermal gradients could not be ruled out. Also, ensuring ideal thermal profiles between microwave and conventionally heated systems remains challenging, increasing the uncertainty of extracting these activation parameters. These challenges make it difficult to distinguish between nonthermal effects from heat or mass transfer contributions with a high degree of certainty.

##### 4.2.2.5 Structural Defect Manipulation

Structural Defect Manipulation refers to the process of inducing imperfections in the materials crystal lattices using externally applied effects such as microwave radiation. Figure 6g represents the intentional generation, migration, or amplification of lattice imperfections such as vacancies, interstitials, and dislocations driven by the interaction between high-frequency electromagnetic fields and the atomic structure of solids. These are used to tailor the physical, chemical, mechanical, optical, or electrical properties of the materials.

UiO-66 is a well-known zirconium-based metal–organic framework (MOF) for gas adsorption and separation, water purification, and catalysis applications. Irradiation of UiO-66 by microwaves resulted in significant alteration of its structural characteristics. Irradiation at lower microwave power induced enhancement of linker and cluster defects, while higher power produced a more crystalline structure with reduced defects. Compared to the conventionally prepared samples, this increase in defectivity produced unusually high CO_2_/N_2_ selectivity.^122^

Microwaves irradiation changed the physical characteristics of NiO nanostructured samples^123^ by inducing an enhancement of dislocation density, distortion, and defects. This increase has been attributed to the microwave nonthermal effect, which tends to increase the number of dislocations in the lattice. This can be useful in narrowing the band gap in the molecules, modifying optical properties, and decreasing in crystal size.

In another study, microwave irradiation of carbon nanotubes in acidic media generated a high density of defects. It also shortened the tube length and opened the tube walls and tips. The defects have been attributed to microwave heating effects which caused rapid superheating and oxidative functionalization. The defective CNTs showed enhanced double-layer capacitance and pseudo capacitance, significantly boosting the performance of supercapacitor electrodes.^124^

##### 4.2.2.6 Crystal Alignment and Texturing

Microwave irradiation can influence crystal alignment or texturing during materials processing (Figure 6h). Microwaves enable accelerated nucleation through isothermal heating, which enhances nucleation and growth kinetics throughout the material. This enhancement results in smaller, more uniform crystallites with tailored morphologies and higher crystallinity. In parallel, the rapid heating rates influence supersaturation dynamics and solvent evaporation. This controls the pore structure, surface area, and aggregation of particles, thereby enabling the creation of materials with improved porosity and specific surface area, along with tunable textural properties.^125^

An example is demonstrated in the synthesis of layered double hydroxides (LDHs). Microwave-hydrothermal treatment significantly improved the crystallinity, structural ordering, and porosity of LDHs. Compared to conventional methods, microwave irradiation yielded smaller hexagonal platelets and increased the interlayer water content. This was confirmed by XRD and FTIR analysis, which showed lamellar and interlamellar organization.^125^

Another example that was observed is the microwave annealing of Au thin films. Microwave annealing induced the alignment of the crystals toward the Au (111) surface. MW irradiation resulted in rapid volumetric heating and generated residual stress and strain fields that supported the reorientation of grains at temperatures much lower than conventional annealing. The resultant thin films had effective out-of-plane alignment in Au thin films within a few minutes, without any concerns related to film dewetting. The resultant thin films can be used for sensors and as strain-relieved substrates that are used in heteroepitaxy.^126^

Texturing and preferential orientation have been also observed in microwave processed samples. For example, a typical hot-pressed silicon sample shows XRD diffraction peaks of (111), (220) and (311) planes. The microwave processed sample can eliminate the peaks of (220) and (311) planes and preferentially align the grains into (111) planes. This phenomenon is probably induced by applied electric field during microwave processing.^40^ Similar results have been observed in the microwave processing of a Bi_2_Te_3_ alloy, where microwave decrystallize the alloy up to 400 °C, first. Then, at temperatures above that, it generates grains with preferred orientations.^40^

##### 4.2.2.7 Alloy Decomposition

Under microwave radiation, alloy decomposition is a phenomenon where individual elements or phases within an alloy undergo solid-state decomposition. This is shown in Figure 6i. This is not observed in conventional processing, as most alloys are stable solid solutions at room temperature. This decomposition is attributed to ponderomotive forces (PMFs), which are nonlinear, time-averaged forces that arise in inhomogeneous electromagnetic fields. Under the polarization of the atomic structure due to electromagnetic fields, mass transport is enhanced, enabling the selective movement of atoms, even without a thermodynamic miscibility gap. This field-driven segregation drives the decomposition of thermally stable alloys into their elemental constituents, which would have otherwise remain homogeneous under thermal annealing. This unique mechanism provides a unique top-down approach to control composition and tune microstructures in microwave synthesized materials.^110^ Due to the differences in dielectric constant and plasma frequency between different components, PMFs act with different magnitude, leading to selective ion motion.

One example of this is the decomposition of silicon–germanium (Si_0.8_Ge_0.2_) alloy. Microwave irradiation induced a phase separation in this thermodynamically stable solid solution. In Si–Gealloys, Geions experience about 60% higher PMF per unit mass than Si ions (Figure 8g). This difference causes compositional segregation. Figure 8h schematically shows the decomposition process under microwave irradiation. SEM image and energy dispersive spectroscopy (EDS) phase analysis confirmed the segregation of the solid-solution into silicon-rich and germanium-rich domains due to asymmetric broadening and shifts (Figure 8i). Additional mechanisms, such as anisotropic field distribution, selective bond softening, and nonequilibrium phonon generation, may also contribute to this phenomenon. This phase-separated structure reduced the bulk thermal conductivity due to increased phonon scattering from the newly introduced interfaces (Figure 8j). These results demonstrated a top-down route to fabricate gradient composites from homogeneous alloys without a long and thermally tedious process.^110^

Another example is the rapid decomposition of manganese ore, which is a multiphase alloy-mixture of MnCO_3_, Mn_2_O_3_, and CaCO_3_. Microwaves enabled selective dealloying of the Mn-rich zones, improving the manganese content from 30% to 42%.^127^

Microwave was also used to completely decompose and recrystallize ZnO in the presence of graphite to yield high-purity single-crystalline wurtzite nanowires. Compared to conventional routes that require long reaction times and growth precursors, microwave method yielded over 95% nanowire, while preserving a clean surface, thereby improving the photocatalytic activity.^128^

### 4.3 Far-From-Equilibrium Phase Formation Paradigm

Through the evolution of microwave heating and synthesis processes, specific processes focused on developing nonequilibrium material systems have emerged. These differ from conventional microwave processing by being ultrafast, reaction-driven, and, in some cases, utilizing plasma environments. They represent a subset of microwave processing that leads to far-from-equilibrium states in materials via distinct energy deposition pathways. These approaches include thermal shock synthesis, microwave combustion (SHS), and microwave-induced plasma-assisted synthesis. In the following sections, the governing physics of these methods, including energy deposition mechanisms, ignition conditions, and heating/quenching behavior, are discussed alongside their characteristic material outcomes.

Microwave Thermal Shock (MWTS) is characterized by the rapid heating of materials to high temperatures followed by instantaneous self-quenching, facilitating the formation of nonequilibrium phases.^129^ Microwaves are absorbed via dipolar polarization of terminal functional groups and conductive losses in partially conductive materials, enabling localized, ultrafast heating.^129,130^ Once sufficient energy is absorbed, the material temperature increases abruptly. Subsequently, the material undergoes rapid quenching as microwave absorption decreases, resulting in extremely high cooling rates.^129,130^ This combination of ultrafast heating and quenching suppresses long-range diffusion, promoting the formation of small, uniformly distributed nanoparticles with high defect densities or metastable phases.^129^ MWTS is typically performed on powders, thin films, or pellets to ensure uniform energy absorption. However, sample size and geometry remain constrained by microwave penetration depth and field distribution, which significantly influence heating uniformity and thermal gradients.^131^

Microwave-assisted self-propagating high-temperature synthesis (SHS) occurs when an exothermic reaction is initiated in a powder compact, triggering a self-sustained combustion wave. This process facilitates the formation of intermetallic, ceramic, or composite phases.^132^ Microwave energy couples with reactants via dielectric, conductive, and interfacial losses, enabling selective volumetric heating. In certain systems, microwave-absorbing media (susceptors) are utilized to enhance energy absorption and facilitate reaction initiation.^133^ Once the ignition temperature is reached, the exothermic reaction becomes self-sustaining and propagates rapidly through the material. The initiation and propagation of the combustion wave are governed by microwave absorption characteristics, reactant stoichiometry, the presence of susceptors, and preheating conditions. The rapid nature of the exothermic reaction limits long-range diffusion, typically resulting in refined microstructures, porous or heterogeneous features, and occasionally the stabilization of metastable phases.^132^ Microwave-assisted SHS is generally performed on compacted powder pellets or bulk samples, where the geometry is designed to support the initiation and stable propagation of the combustion front.

Microwaves can generate plasma by coupling the electromagnetic field with gaseous molecules, leading to their dissociation and the subsequent formation of reactive species.^134–136^ As a highly reactive environment, microwave-assisted plasma synthesis is governed primarily by plasma kinetics, gas-phase heating, and species transport, rather than the bulk heating of solids.^134,135^ Once sufficient microwave power is applied, plasma is generated and sustained, with the onset of reactions depending on factors such as power density, gas concentration, and flow conditions.^135,136^ This process provides a stable, highly reactive region for material transformation. Upon removal from the plasma environment, materials undergo cooling, enabling the rapid synthesis of high-purity products characterized by fine particle sizes, narrow distributions, and controlled morphologies. Furthermore, the high reactivity of the plasma promotes the formation of nonequilibrium, defect-rich regions with metastable compositions and unique core–shell or hybrid architectures.^136^ However, the technique remains limited by reactor design, electromagnetic field configurations, and gas flow dynamics, which collectively influence plasma stability and reaction uniformity.^135,136^

Through rapid synthesis, defect engineering, and advanced manufacturing routes, these approaches enable material processing pathways that are not easily accessible through conventional microwave processing. Governed by distinct energy deposition and reaction mechanisms, these paradigms are increasingly vital for accessing far-from-equilibrium material phases. Representative examples of these techniques are discussed in the following sections.

## 5 Microwave Processing of Advanced Materials

Microwave processing is a distinct method for synthesizing, processing, and engineering advanced materials. Its rapid, uniform, and volumetric heating capabilities lead to faster processing cycles, improved structural control, and energy savings. A summary of the applications across various material systems is provided in Table 6. The following sections explore how microwave processing enhances the formation of advanced materials.

**Table 6:** Summary of Applications of Microwave Processing across Various Material Systems

Application Domain	Examples	Notes	Reference
Ceramics	Al_2_O_3_	Lower sintering temperatures, with high densities (∼98% theoretical)	^137^
	SiC	Higher densification (83.6%) compared to conventional (50%)	^138^
	Yttria Alumina Garnet (YAG)	Rapid single-phase nanocrystal formation (<30 min) with reduced temperature and particle size	^139^
Functional Electronic Materials	ZnO Varistors	Rapid densification (>90%) with improved electrical performance	^140^
	LaAlO_3_/LaMnO_3_ for SOFCs	Phase-pure complex oxides synthesized in ∼30 min	^64^
	boron-doped Cz Si solar cells	Ultra fast defect passivation (2–4 s) of B–O defects, improving device efficiency	^141^
Energy Storage Materials (Batteries)	LiV_3_O_8_ cathode	Enhanced Li^+^ ion transport, capacity, and cycling stability by nanostructuring and defect formation	^142^
	Si anode	Rapid synthesis of uniform nanoparticles, reduced volumetric expansion and improving cycling stability	^136^
Energy Storage Materials (Supercapacitors)	Ni(OH)_2_	Rapid synthesis of phase-pure nanosheets with improved capacitance	^143^
Energy Storage Materials (Fuel Cells)	Mn_3_O_4_@RGO	Nanocomposites with enhanced catalytic activity and stability for ORR	^144^
Thermoelectric Materials	BiTe alloys	High Seebeck coefficients, low thermal conductivity, and improved zT	^145–148^
	TiNiSn/TiCoSb half-Heusler alloys	Improved zT and reduced thermal conductivity	^149^
	SiGe	Introduction of dislocations and reduced thermal conductivity, high zT	^40^
Magnetic Materials	Ni–Zn ferrite - Ni_1‑x_Zn_*x*_Fe_2_O_4_	Reduced synthesis temperatures, improved saturation magnetization, and uniform microstructure	^150^
	NiMgZn ferrites - Ni_0.2_Mg_0.8–x_Zn_*x*_Fe_2_O_4_	Low coercivity with a tunable saturation magnetization	^151^
	Mn-doped (V,Mn)_2_AlC	Reduced processing time and energy consumption	^152^
Carbonaceous Materials	Diamond/Co composites	Preservation of metastable diamond phase by reducing graphitization	^153^
	Reduced graphene oxide (RGO)	Uniform nanostructures with reduced agglomeration enabled by ultrafast synthesis	^129^
	Few-layer graphene	Rapid and catalyst-free synthesis, high purity and controlled thickness	^134^
Hydrogen storage and production materials	MgH_2_–TiO_2_	Hotspots formation favors hydrogen desorption at lower temperatures and promotes reabsorption at room temperature	^154^
	Liquid organic hydrogen carriers (LOHCs)	Volumetric heating improves hydrogen removal, catalytic activity, and energy efficiency	^155^
	Hydrogen-rich plastics	Enhanced hydrogen yield and reduced operating temperatures	^156^
Metals and Alloys	Cu-steel alloys	Enhanced mechanical properties	^157^
	Refractory metals (W, Mo, Re)	Rapid heating prevents excessive grain growth leading to enhanced density (>95%)	^158^
	Ti, Mg, Al alloys	Refined microstructure leads to improved hardness and tensile strength	^159–162^
Composites	Al_2_O_3_–ZrO_2_	Rapid heating enables high density with homogeneous microstructure and low grain coarsening	^163^
	WC-Co composites	Densification at lower temperatures, improved hardness and toughness	^164^
	Functionally Graded W/Cu	Varying porosity and composition through selective heating	^165^
Catalysts	Au-nanoparticles	Shape-controlled particles with narrow size distribution	^166,167^
	TiO_2_	Rapid anatase-to-rutile phase transformation	^168^
	Metal–organic frameworks (MOFs)	Accelerated nucleation and growth, reducing synthesis time and increasing yield	^169^
2D Materials	Hematane	Single-step scalable synthesis with high crystallinity and low defects	^170^
	LaMn_*x*_Ni_1–x_O_3_ perovskite	Formation of porous Ni-rich 2D structures with enhanced conductivity and catalytic activity	^171^
	MXene	Accelerated etching and delamination, enabling rapid, scalable, and HF-free synthesis	^172^
High Entropy Alloys (HEAs)	FeCoNiCrAl	Rapid formation of BCC solutions with reduced energy input	^173^
	HEA/GO nanocomposite	Co-reduction of metal species onto graphene enabling uniform elemental distribution and preventing phase segregation	^174^
	Mesoporous RhAgCuPdPt HEA nanoparticles	Homogeneous multimetal reduction and uniform mesoporosity	^175^
Organic Materials	Peppermint (*Mentha piperita L*.)	Improved solvent penetration, higher yield, and reduced processing time, solvent use, and energy consumption	^176^
Polymers	Poly(ε-caprolactone)	Rapid volumetric heating, tunable kinetics, minimal thermal degradation	^177^
	Polystyrene (NMRP)	Higher conversion rates with narrow molecular weight distribution	^178^
Biomaterials	Metallic biomaterials	Improved densification and mechanical properties, refined microstructure	^179^
	ZrO_2_	Rapid densification, fine grain size (∼0.5 μm) with high density (∼96%)	^180,181^
	Hydroxyapatite (HAP)	Higher density, uniform grains, reduced porosity, and improved mechanical strength and bioactivity	^182,183^

### 5.1 Microwave Processing of Ceramics

Dielectric and insulating materials are highly effective absorbers of microwave energy. They polarize by interacting with electromagnetic fields to form dipoles that oscillate with the electric field, making them ideal for microwave-assisted sintering and synthesis. They convert microwave energy into heat by dielectric loss mechanism, which is particularly suitable for microwave-assisted processing applications.^184^ In polar ceramics and oxides, molecules attempt to align with the alternating microwave field, leading to energy dissipation as heat. Ionic moments in partially conducting ceramics further contribute to microwave coupling at elevated temperatures. This volumetric heating ensures rapid and uniform energy dissipation, minimizing thermal gradients and enabling homogeneous microstructures.^185^

Within dielectric materials, various mechanisms contribute to energy loss. In polar ceramics, dipolar polarization is the dominant mechanism, where permanent or induced dipoles rotate in response to the microwave field.^27^ In systems with heterogeneous phases or inclusions, such as doped ceramics and oxide composites, interfacial polarization (also known as the Maxwell–Wagner–Sillars effect) becomes more prominent. This occurs when charge carriers accumulate at the interfaces between regions with different dielectric constants, generating additional heating and enhancing the localized field.^185^ In materials like partially conducting ceramics or those with mixed-valence ions, electron hopping conduction is observed. Here, electrons jump between adjacent charge centers, dissipating energy as they move.^186^ At elevated temperatures, ionic conduction contributes further to microwave interaction, as mobile ions migrate through the lattice under the influence of the electric field.^21^ The presence of these multiple and often overlapping mechanisms enables fine control over the heating profile and sintering dynamics.

Microwave heating significantly reduces processing times compared to traditional methods. For example, microwave sintering can fully densify ceramics like alumina and zirconia in under 30 min, whereas conventional processes take several hours.^187,188^ In addition to reduced processing time, microwave processing offers several other advantages over traditional methods for synthesizing and processing advanced ceramics, including enhanced sintering performance and material properties; flash sintering of high-performance ceramics; and expanded applications and improved efficiency of ceramics.

Microwave sintering of alumina (Al_2_O_3_), a key ceramic for structural and electronic applications, has achieved 98% density at temperatures approximately 200 °C lower than conventional methods, while maintaining similar densification.^137^ Figure 9a compares the densification kinetics at various temperatures for conventional and microwave synthesis routes, respectively. The microwave-sintered sample reached a near-full density at 1350 °C in under 50 min. In contrast, at similar temperatures and times, conventionally sintered samples only reached 60% density. Microwave sintered samples achieved high densifications even at temperatures as low as 1150 °C, with a sintering time of around 200 min.

**Figure 9:**
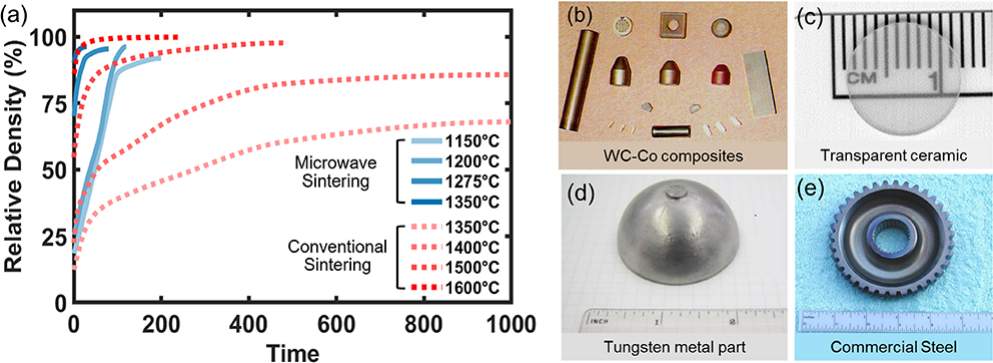
(a) Comparison of the densification kinetics for alumina sintering at various temperatures for conventional and microwave sintering. Reproduced with permission.^137^ Copyright 2004 John Wiley and Sons. Optical images of various microwave sintered materials: (b) WC-Co composites—reproduced with permission.^190^ Copyright 2013 Elsevier, (c) Transparent ceramics—reproduced with permission.^191^ Copyright 2002 Elsevier, (d) Tungsten metal part—reproduced with permission.^190^ Copyright 2013 Elsevier, and (e) Commercial Steel—reproduced with permission.^190^ Copyright 2013 Elsevier.

Microwave-assisted self-propagating high-temperature synthesis (SHS) has been employed to rapidly synthesize SiC from a powdered mixture of silicon and carbon. Microwave energy was used to trigger a combustion reaction that propagated throughout the sample. Unlike conventional ignition methods, microwave initiation led to internal ignition via volumetric heating, with the combustion wave propagating radially outward from the center. This enabled a high relative density of up to 83.6%, which was significantly higher than the ∼50% achieved conventionally.^138^

Researchers have also demonstrated ultrafast, or flash sintering, of high-performance ceramic materials, for structural, optical, and electronic applications, using a 24 GHz gyrotron microwave system. This method has achieved over 98% of theoretical density in just a few minutes for materials including Al_2_O_3_, ZrO_2_, Y_2_O_3_, MgAl_2_O_4_, and Yb:(LaO)_2_O_3_.^189^

Yttria Alumina Garnet (YAG) is an important oxide ceramic used in lasers, high-power white lighting, transparent optical windows, and as scintillators for photodetectors. Microwave-assisted solvothermal processing was used to rapidly synthesize optical-ceramic YAG powders. In 1,4-butanediol, single-phase nanocrystalline YAG was formed in under 30 min, avoiding intermediate stages. This resulted in particles sized between 60 and 140 nm at 280–290 °C. Compared to conventional solvothermal routes, the microwave method lowers the temperature, time, and pressure required to produce powders for transparent ceramics.^139^

Microwave processing also enables the sintering of advanced high temperature ceramics like borides and carbides. Titanium diboride (TiB_2_), a ceramic widely used in armor, corrosion and wear resistance applications, reached a 98% theoretical density in just 15 min at 2100 °C using microwave sintering.^192^ Additionally, refractory carbides like niobium carbide (NbC) and tantalum carbides (TaC), which are common in cutting tools and high-wear components, have been successfully fabricated with Ultrarapid Microwave Heating (URMW). This process requires significantly lower temperatures (as low as 1350 °C for NbC and 1200 °C for TaC) compared to conventional methods (1400–1500 °C), leading to substantial energy savings at larger scales. The process is also faster and does not require a vacuum or controlled gas environment.^193^

Microwave-assisted SHS has also been applied to produce Ti_2_AlC MAX phase.^133^ Microwave irradiation selectively heats the reactants, initiating combustion within seconds, leading to the rapid formation of Ti–Al intermetallics and TiC intermediates. This is followed by a transformation into Ti_2_AlC MAX phase at temperatures exceeding 1600 °C. The resulting material is porous and exhibits plate-like nanolaminated features, making it highly suitable for high-temperature structural and multifunctional applications.

Microwave technology is also being studied for sintering large ceramic parts, particularly for refractory materials used in channel induction furnaces. It offers more homogeneous heating and improved energy efficiency compared to traditional resistance heating methods, which suffer from poor thermal conductivity and uneven densification, leading to long processing times and premature failure.^194^

Furthermore, microwave heating has been applied to both the drying and sintering processes of ceramic production, including materials composed of kaolin, ball clay, feldspar, and quartz. Its internal volumetric heating efficiently removes bound water, promoting grain boundary diffusion and enabling enhanced shrinkage control. This results in superior or comparable properties to those achieved by conventional processing, while using less energy.^195^ This versatility is demonstrated by Figure 9b and c, which shows multiple microwave sintered ceramics such as WC-Co composites and transparent alumina.

These advancements demonstrate that microwave processing is not just a tool for property improvement but represents a radical shift in the fabrication of advanced ceramics. By applying a fundamentally different mechanism of energy transfer, it overcomes conventional limitations such as slow speeds, high energy consumption, and thermal gradient formation. The unique capabilities of this approach, including rapid densification at lower temperatures, refinement of grain size, and the ability to preserve complex geometries, open a new space for creating ceramics with superior mechanical, optical, and thermal properties. Microwave processing is thus a critical enabler of next-generation high-performance materials, driving innovation in the ceramics landscape.

### 5.2 Microwave Processing of Functional Electronic Materials

Electroceramics, such as ZnO varistors, BaTiO_3_/SrTiO_3_ and Gd:CeO_2_ solid electrolytes, are critical in various applications such as surge protection, capacitors, and solid oxide fuel cells (SOFCs). Rapid microwave sintering has achieved high theoretical densities (>90%) within minutes, eliminating the need for the prolonged thermal exposure required by conventional methods. The selective absorption and volumetric heating of microwave induce thermal instabilities that trigger early densification, finer microstructural control, and enhanced functional properties. These effects are demonstrated in the SEM images which reveal distinct microstructural characteristics based on different heating effects. Direct microwave heating caused grain surfaces to deform as if they were softened. They also had higher average grain sizes (Figure 10a). Susceptor-assisted microwave heating maintained shorter average grain sizes and produced finer microstructure (Figure 10b), and in contrast, conventional sintering produced coarser microstructures (Figure 10c). The microwave process not only reduces both processing time and energy consumption, but also enables grain-size control, which results in superior electrical performance in ZnO varistors.^140^

**Figure 10:**
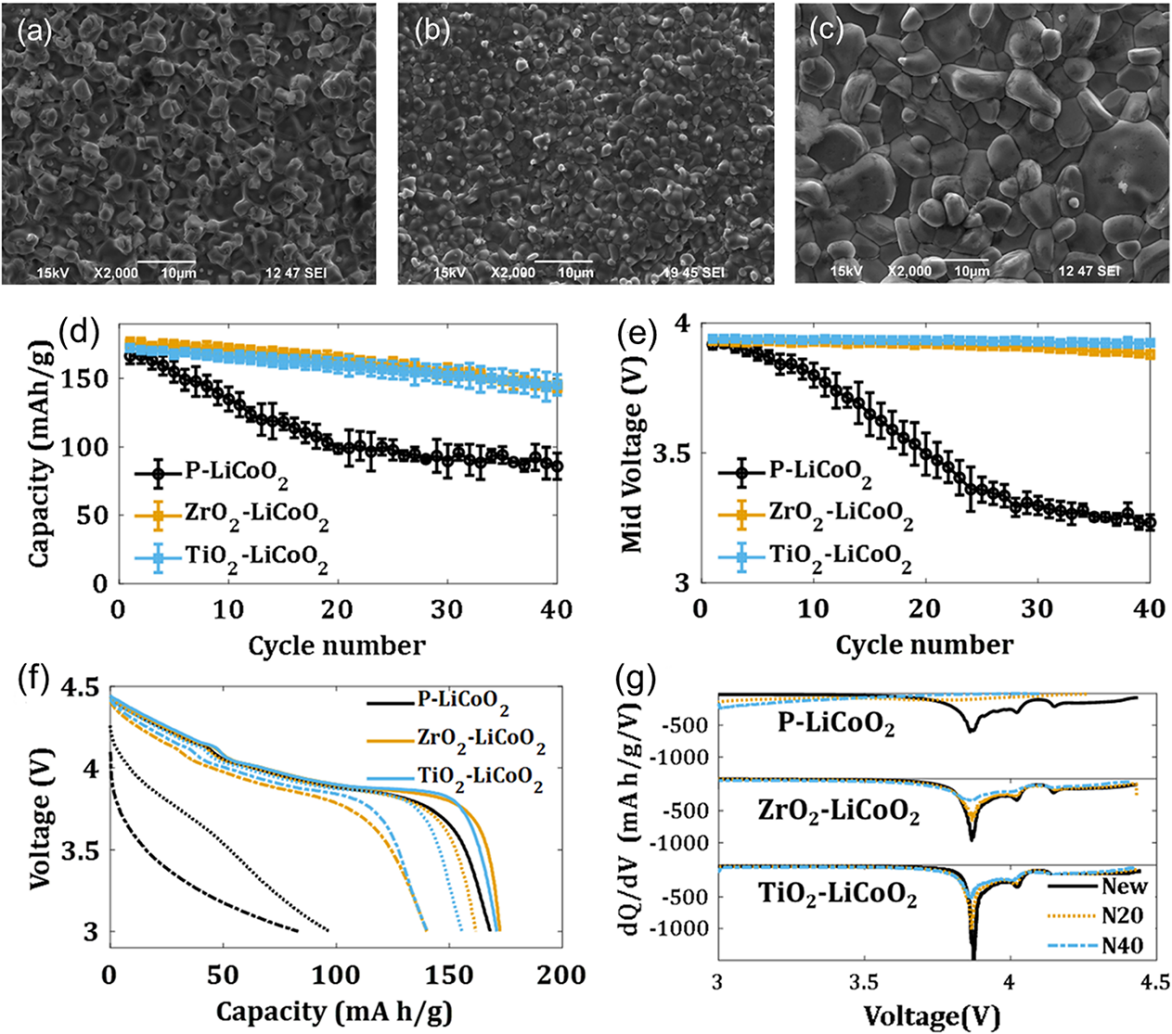
SEM micrographs of ZnO-based varistor ceramics processed by different heating routes: (a) direct microwave heating at 30 °C/min (no hold), (b) susceptor-assisted microwave heating at 30 °C/min (no hold), and (c) conventional heating with a 30 min isothermal hold. Reproduced with permission.^140^ Copyright 2023 Elsevier. High-voltage stability of lithium cobalt oxide (LCO) electrodes with and without microwave-assisted ZrO_2_ and TiO_2_ coatings: (d) Capacity retention over 40 cycles. (e) Evolution of midpoint discharge voltage during cycling. (f) Galvanostatic discharge curves (C/2) at the beginning of life, and after 20 and 40 cycles. (g) Incremental capacity analysis (ICA) corresponding to the discharge profiles in (f). Reproduced from permission.^196^ Copyright 2020 Springer Nature.

Microwave-assisted hydrothermal processing has also been used to influence the phase evolution and functional properties of BaTiO_3_. Compared to conventional synthesis, microwave enables rapid crystallization, with the cubic phase forming within minutes compared to hours under conventional processing. Under microwave processing, cubic to tetragonal phase transition is enhanced, leading to higher tetragonality (c/a ratio). This transition governs the ferroelectric and dielectric behavior. This phase transformation is attributed to the volumetric heating achieved by the microwave-material coupling, which accelerates reaction kinetics and promoted the efficient formation of the functional tetragonal phase.^197^

Microwave-assisted solid-state synthesis has been utilized to fabricate transparent conductive indium tin oxide (ITO). The reaction time was reduced from several hours to ∼10 min due to enhanced localized heating, which accelerated solid-state diffusion. However, nonuniform temperature distribution can limit phase conversion. To address this, the process was modified to incorporate intermittent mixing, resulting in improved phase formation and improved electrical conductivity compared to conventionally processed and commercial ITO materials.^198^

Microwave-assisted processing has been employed in the synthesis of single-phase Lead Zirconate Titanate (PZT) ferroelectric and piezoelectric materials at temperatures as low as 600 °C within minutes, compared to conventional methods that require ∼800 °C and take several hours. This acceleration in kinetics is attributed to the enhanced diffusion and microwave-material interactions, which alter the reaction pathway and significantly reduce the volatilization of PbO during processing.^199^

Microwave processing is also effective in fabricating various complex oxides, including LaAlO_3_, LaCrO_3_, LaMnO_3_, LaFeO_3_, and LaCoO_3_, which are used as components for SOFCs, catalysts, membranes. Unlike conventional methods that require temperatures over 1000 °C for more than 24 h, microwave processing can synthesize these materials in just 30 min. Early formation of metallic clusters acts as susceptors, enabling localized superheating and accelerated crystallization.^64^

Microwave-assisted synthesis was explored for the fabrication of high-temperature superconducting oxide of YBa_2_Cu_3_O_7_ (YBCO). Microwave processing reduced the processing time from several hours to minutes (∼15 min) compared to conventional sol–gel routes. The microwave processing also exhibited an improvement in the superconducting performance with a higher critical temperature (T_C_) of 94 K compared to 92 K using sol–gel methods. The grain sizes were finer and the structures were more homogeneous with enhanced grain boundary conditions.^200^

Microwave-assisted combustion synthesis has also been employed for rapid formation of complex superconducting oxides. In thallium-based cuprates, ultrafast SHS and microwave combustion have been used to reduce reaction times from hours to seconds, while minimizing the loss of volatile thallium, thereby enabling phase formation. Compared to SHS, microwave combustion provided more uniform heating, resulting in higher phase purity and improved reaction completeness. To achieve fully dense materials, thermal treatment is performed after synthesis.^132^

Furthermore, microwave-assisted synthesis has become popular for producing zinc oxide (ZnO) nanostructures. ZnO is a multifunctional material with applications in piezoelectrics, solar cells, supercapacitors, batteries, and sensors. Conventional methods often result in poor reproducibility, inconsistent morphology, and broad particle distribution. By contrast, microwave synthesis can produce precise morphologies with higher purity and precise doping characteristics, all within minutes.^201^

In commercial photovoltaic modules, Czochralski Silicon cells are commonly used. Microwave have been used to anneal boron-doped Czochralski silicon solar cells. This process passivates boron–oxygen (B–O) defects, which are known to cause light-induced degradation (LID) and reduce cell efficiency. Through conduction losses, microwave enable rapid, volumetric heating, reaching temperatures of approximately 500 °C in less than 2 s and completing defect passivation in just 2 to 4 s. Compared to conventional rapid thermal annealing (RTA), microwave annealing offers similar defect recovery with a significantly shorter processing time and lower energy consumption.^141^

These examples demonstrate that microwave processing is a powerful technique for engineering and improving the performance of functional electronic materials. Its advantages over conventional techniques are highlighted by its capabilities, including the precise control of grain size, accelerated synthesis of phase-pure complex oxides, and rapid passivation of performance-degrading effects. This targeted control over microstructure provides a direct pathway to create high-performance materials with superior and more reliable functional properties.

### 5.3 Microwave Processing of Batteries and Other Energy Storage Materials

In modern energy storage systems, batteries, supercapacitors, and fuel cells are essential for enabling portable electronics, electric vehicles, and the integration of renewable energy sources. Many of the materials used in batteries and supercapacitors are ceramics, such as oxides, sulfides, or layered hydroxides. Fuel cells, on the other hand, rely on catalytic materials with high activity and stability. The electrochemical performance of energy storage materials relies heavily on the ionic transport, electronic conductivity, electrode–electrolyte interfacial stability, and the ability of the structure to remain stable during repeated charge–discharge cycling.^202^ A key challenge in these systems is enhancing the ion transport and interfacial kinetics while maintaining the structural stability of the material during repeated cycling,^203^ which requires a precise control over the microstructure,^204^ interfaces,^205^ and defect through defect engineering.^206^ Microwave processing directly influences these parameters by promoting rapid volumetric heating, and the formation of defect-engineered and nanostructured materials, while offering a rapid, energy-efficient route to synthesize these materials with improved morphology, crystallinity, and electrochemical performance.

Microwave synthesis has been demonstrated as a rapid and energy-efficient route for the synthesis of P2- and O3-type Na_*x*_CoO_2_ phases, which are promising cathodes for sodium-ion batteries. While the phase transformation process remains similar, the use of microwave accelerates reaction kinetics, leading to faster processing and reduced energy input, while yielding materials with comparable structure, morphology, and electrochemical performance.^207^

Solid-state microwave synthesis was used to produce LiV_3_O_8_ cathode material. The reaction pathway is similar to conventional methods, however, the use of microwaves accelerated the kinetics and produced unique nanosheets and nanorod morphologies. These microstructural variations and increase in defect concentration influenced the Li^+^ pathways and electrochemical behavior leading to improved ionic conductivity, higher discharge capacity, and better cycling stability compared to conventional methods.^142^

Microwave-assisted processing has also been employed to synthesize Cu_7.2_S_4_ nanotubes with defect-rich structures as electrodes for magnesium batteries. The rapid and localized heating facilitated efficient etching and phase transformations within a short duration that enabled the formation of hollow tubes with defect-rich lattice features.^208^ The resulting Cu_7.2_S_4_ nanotubes exhibit a high surface area and interconnected one-dimensional morphology, which enhance ion transport and accommodate lattice strain during cycling. Kinetic reactions are further improved by reducing diffusion barriers and providing additional active sites. This demonstrates that microwave processing is an effective tool for synthesizing Cu_7.2_S_4_ nanotubes with high specific capacity, excellent rate capability, and ultralong cycling stability over extended charge–discharge cycles.

Microwave-assisted synthesis has been also employed to synthesize LiMn_1.5_Ni_0.5_O_4‑δ_ spinel cathodes. Microwave treatment enabled nanostructuring and modified the ratio of Mn^3+^/Mn^4+^ and Nicontent, leading to changes in the lattice parameter and electrochemical behavior. The reduction in particle size and the presence of oxygen deficiency improved the lithium-ion diffusion kinetics and enhanced capacity and rate performance.^209^

Microwave plasma synthesis has been employed to produce silicon nanoparticles, which are used as high-capacity anode materials. Atmospheric-pressure microwave plasma enabled rapid vaporization and nucleation of silicon, leading to the formation of nanoparticles within milliseconds. The resultant Si nanoparticles have a small particle size and narrow size distribution, which helps mitigating volume expansion and can improve cycling stability in battery systems.^136^

Microwave processing has also been utilized to synthesize ceramic-based, all solid-state battery systems. In lithium lanthanum zirconium tantalum oxide (LLZTO), the carbothermal shock method enables the densification of the garnet electrolyte rapidly within seconds, while minimizing the loss of volatile elements, facilitating simultaneous cosintering of the electrode and electrolyte material, resulting in a fully integrated structure.^131^

Microwave-assisted synthesis has also been applied to sulfide solid electrolytes used in solid-state sodium batteries. Microwave irradiation provided a simple, accelerated, and energy-efficient route to obtain highly crystalline Na_3_PS_4_ with appreciable ionic conductivity and low activation energy for Na^+^ transport. The rapid heating and sudden cooling promoted phase formation within a short duration. A prototype cell using Na_3_V_2_(PO_4_)_3_/Na_3_PS_4_/Na configuration demonstrated promising electrochemical properties.^210^

Microwave-assisted methods are also highly effective for fabricating supercapacitor materials. For instance, nickel hydroxide, Ni­(OH)_2_, a key pseudocapacitive material, was synthesized as pure α-phase nanosheets directly on stainless-steel foil in just 10 min at 150 °C using a microwave-assisted hydrothermal method. This process provided excellent control over phase and morphology without any additives, achieving rapid, and improved capacitance compared to conventional methods that require several hours.^143^ MnCo_2_O_4.5_ and Ni_3_S_2_ are complementary redox-active materials which are used in hybrid battery-type supercapacitor electrodes. Microwave assisted dual step hydrothermal method was used to grow vertically aligned MnCo_2_O_4.5_ nanoarrays with interconnected Ni_3_S_2_ nanosheets on nickel foam. This strong bonding between active layers and current collectors minimized resistance and resulted in high areal capacities.^211^ Similarly, NiS_2_/Ni­(OH)_2_ grown on nickel foam, lead to an energy storage device with high energy density and excellent capacitance retention.^212^

Microwave processing has been used to demonstrate the synthesis of TiO_2_ or ZrO_2_ coatings on lithium cobalt oxide (LCO) cathode. The coatings improved the rate performance and cycling retention of these cathodes while limiting the dissolution of cobalt and the growth of charge-transfer resistance. As shown in Figure 10d–g, coated electrodes retained over 80% of their capacity after 40 cycles at 4.5 V, compared to only ∼53% for pristine LCO cathodes, while also maintaining a stable discharge profile and voltage plateau.^196^

In fuel cells, microwave-assisted hydrothermal techniques have enabled the one-step synthesis of Mn_3_O_4_ intercalated into reduced graphene oxide (rGO). The Mn_3_O_4_@rGO composite acts as a crucial catalyst for the oxygen reduction reaction. The rapid process resulted in nanocomposites with enhanced stability and improved properties for fuel cell applications.^144^

Fe_3_O_4_–GO nanocomposites, which are used as efficient bifunctional electrocatalysts for oxygen evolution and reduction reactions, were synthesized using a microwave-assisted hydrothermal method. Microwave-induced rapid and volumetric heating enabled the formation of ultrafine Fe_3_O_4_ nanoparticles that are densely and uniformly dispersed on GO sheets. This resulted in improved charge-transfer characteristics and enhanced electron transport within the composite. The enhanced performance can be attributed to the improved dispersion and the high-surface-area structure achieved through microwave processing, demonstrating its potential for electrocatalytic applications.^213^

Microwave-assisted synthesis was also used to prepare nitrogen-doped graphene (NG) by rapidly heating graphene sheets in an ammonia-rich environment. Both undoped graphene and NG were then incorporated into platinum catalysts using a microwave-assisted polyol process. The Pt/NG catalyst exhibited improved Pt dispersion, a higher surface area leading to improved electrochemical activity, and enhanced tolerance from CO poisoning. Compared to the Pt/G catalyst, which acted as the baseline for this study, the Pt/NG catalyst demonstrated superior catalytic performance in fuel cell applications with long-term stability.^214^ By enabling precise control over the crystal phase and creating unique nanostructured morphologies, microwave processing addresses some of the current challenges in the field of energy storage materials. Through defect engineering and control over the interfacial characteristics, it enhances ion transport, charge transfer kinetics, and improves cycling stability in energy storage systems. It therefore offers a powerful route to fabricating next-generation cathodes, high-performance supercapacitors, and fuel cell catalysts, thereby accelerating the development of more reliable and efficient energy storage and conversion technologies.

### 5.4 Microwave Processing of Thermoelectric Materials

Another important class of materials in energy conversion technology is thermoelectric materials. They play a vital role by directly converting heat into electricity and vice versa, which is useful for waste heat recovery and solid-state cooling. The efficiency of a thermoelectric material is governed by a dimensionless figure of merit, zT, defined by $zT=\frac{\sigma S^{2}}{\kappa }T$ . Here σ, S, κ, and T are electrical conductivity, Seebeck coefficient, thermal conductivity, and absolute temperature, respectively.^215^ A higher zT value indicates better thermoelectric material. A fundamental challenge to improve the zT lies in developing materials with reduced thermal conductivity while having improved electrical conductivity and Seebeck coefficient. Microwave processing emerges as a unique and powerful tool to address this challenge by enabling nanostructuring, phase purity, decrystallization, decomposition, defect engineering, and the formation of nonequilibrium phases, all of which optimize the electrical and thermal transport properties of materials.^40^

Microwave-induced field decrystallization can significantly decrease the thermal conductivity of materials. This phenomenon has been observed in several thermoelectric materials. Unlike conventional heating, using microwave can generate lattice disorder, phase transitions, and reduced thermal conductivity through electronic and field-drive mechanisms. This is not achievable simply by increasing temperature. For example, the thermal conductivity of single crystal silicon can drop by an order of magnitude under microwave irradiation which demonstrates a microwave-induced nonthermal effect.^109^

Nozariasbmarz^40^ extensively studied the effect of microwave processing on the in situ sintering decrystallization of various thermoelectric materials. Several studies have shown that the thermoelectric properties of Bi_2_Te_3_-based alloys can be improved via microwave processing. It was revealed that microwave-induced decrystallization helps forming amorphous–crystalline nanocomposites with high Seebeck coefficients, low thermal conductivity, and enhanced zT in both p-type Bi_0.34_Sb_1.66_Te_3_ ^145,146^ and n-type Bi_2_Te_3–x_Se_*x*_ ^147,148^ alloys. Figure 11a shows the XRD patterns and the scanning transmission electron microscopy (STEM) image of the Bi_0.5_Sb_1.5_Te_3_ alloy before and after microwave. The uniform alloy gets decomposed into Bi_2_Te_3_-rich and Sb_2_Te_3_-rich zones.

**Figure 11:**
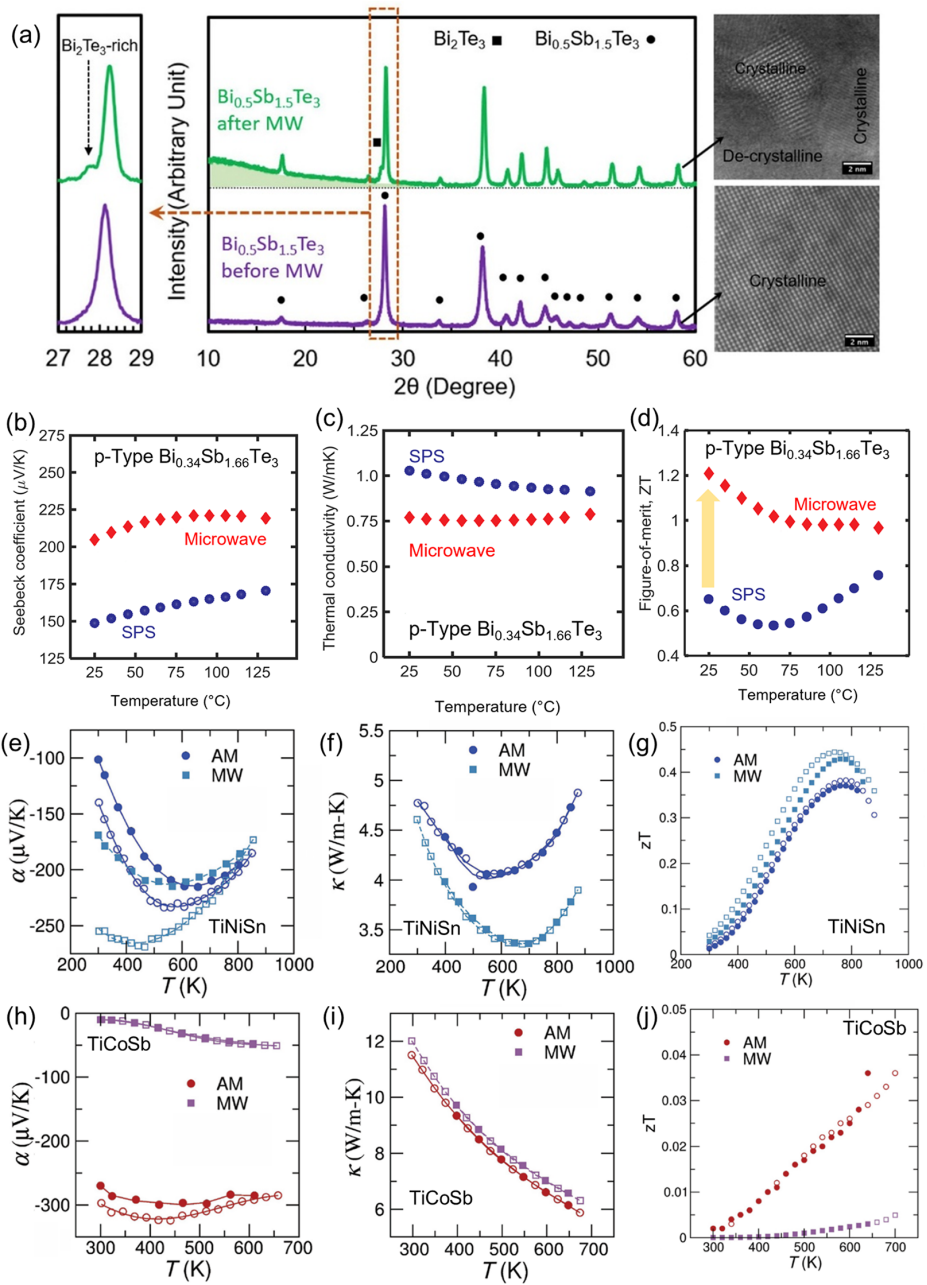
(a) XRD and STEM images of Bi_0.5_Sb_1.5_Te_3_ thermoelectric materials before and after microwave processing. Comparison of the thermoelectric properties of SPS and microwave processed Bi_0.34_Sb_1.66_Te_3_ alloy: (b) Seebeck coefficient, (c) thermal conductivity, and (d) zT. Reproduced with permission.^146^ Copyright 2020 Elsevier. Comparison of the thermoelectric properties of arc-melted (AM) and microwave (MW) processed half-Heusler compounds: (e-g) Seebeck coefficient, thermal conductivity and zT of TiNiSn alloy. (h–j) Seebeck coefficient, thermal conductivity and zT of TiCoSb alloy. Reprinted (adapted) with permission:^149^ Birkel, C. S.; Zeier, W. G.; Douglas, J. E.; Lettiere, B. R.; Mills, C. E.; Seward, G.; et al. Rapid Microwave Preparation of Thermoelectric TiNiSn and TiCoSb Half-Heusler Compounds. Chem. Mater. 2012, 24 (14), 2558–2565. Copyright 2012 American Chemical Society.

Figure 11b–d compared the Seebeck coefficient, thermal conductivity, and zT of p-type Bi_0.34_Sb_1.66_Te_3_ alloy prepared by spark plasma sintering (SPS) and microwave processing, respectively. Microwave processed sample clearly shows higher Seebeck coefficient, lower thermal conductivity, and higher zT, which are all required for a high-performance thermoelectric material.^148^ This microwave-processed sample is particularly useful for applications involving wearable thermoelectric generators, where low thermal conductivity is essential.^145^ Compared to conventional methods, microwave processing offers superior tuning of material microstructure, leading to up to 600% higher power output in self-powered wearable applications.^147^ When compared to SPS technique, the microwave processing of n-type Bi_2_Te_2.7_Se_0.3_ enabled a superior Seebeck coefficient, low thermal conductivity, and high overall performance. Microwave enhances TE properties by inducing nonequilibrium decrystallization and promoting disorder. Compared to conventional synthesis, microwave synthesis offers rapid and volumetric heating with better control over grain boundary structures, resulting in higher electrical performance and improved tunability.^148^

In another study, microwave processing enabled rapid synthesis of doped nanoplatelets of Bi_2_Te_3_ and Sb_2_Te_3_, followed by compaction and sintering into dense pellets with controlled structure. Microwave synthesis enabled faster reaction times, improved scalability, and enhanced control compared to conventional routes, which resulted in improved thermoelectric performance.^216^ Generated nanograin boundaries and nanopores dominate phonon scattering, accounting for nearly 65% reduction in lattice thermal conductivity. In (Bi_*x*_Sb_1–x_)_2_Te_3_ nanocomposites, high-efficiency performance was achieved through microwave processing by inducing nonequilibrium nanostructures and phase decomposition.^145^ Additionally, cold-pressed p-type and n-type Bi_2_Te_3_ pellets were sintered using microwave at 250–200 °C, which led to improved thermal stability below 350 °C with consistent mechanical strength.^217^

TiNiSn and TiCoSb half-Heusler compounds are promising materials for high-temperature thermoelectric applications due to their high power factors, structural stability, and high zT at elevated temperatures. Microwave processing has been successfully used to synthesize these compounds. Figure 11e–j compares the thermoelectric properties of these alloys made of microwave (MW) processing and arc-melting (AM). In TiNiSn alloys, compared to conventional arc-melting, microwave processing enhances the zT. Compared to traditional methods, microwave processing offers a faster and more energy efficient route with comparable or even better zT values.^149^ Additionally, half-Heusler compounds typically have very high thermal conductivity. Microwave processing is an ideal technique to control and reduce the thermal conductivity of these alloys while maintaining high Seebeck coefficient and electrical conductivity.

Silicon germanium (SiGe) alloy is a pioneering and highly important thermoelectric material used in radioisotope thermoelectric generators (RTGs) for space exploration. Microwave processing has been successfully applied on p-type SiGe alloys, resulting in enhanced thermoelectric properties. Microwave processing has replaced the long synthesis processes of SiGe, such as milling, annealing, or melting techniques. Microwave enables in situ synthesis and sintering of the alloys in a much faster and more energy-efficient way. In this alloy, microwave processing can also generate a high density of dislocations, which reduces the thermal conductivity of the alloy while maintaining or improving its other properties.^40^

Therefore, microwave processing can improve the thermoelectric properties of various materials through the formation of nonequilibrium defects and compounds, as well as by creating decrystallized structures, thereby increasing the density of phonon-scattering effects. This directly addresses one of the core challenges in developing thermoelectric materials with higher zT by selectively affecting both thermal and electrical conductivities. This ability to precisely manipulate material structure is critical for the development of advanced thermoelectric materials. However, a comprehensive understanding of the nonthermal effects of microwave irradiation on the microstructure and nonequilibrium phases still require extensive study.

### 5.5 Microwave Processing of Magnetic Materials

Magnetic and thermomagnetic materials are an important class of materials. Magnets enable the conversion between mechanical and electrical energy in applications such as motors and generators.^218^ Their performance depends on tuning the phase composition, grain size, and defect chemistry, which can alter properties such as magnetization, coercivity, and Curie temperature. Conventional synthesis routes face fundamental challenges which often require prolonged, energy-intensive processes that restrict the flexibility of composition. In contrast, microwaves offer rapid and volumetric heating with tunability of the phases and nanostructures, thereby enhancing the functional properties.

Microwave heating combined with spark plasma sintering was used to synthesize bulk V_2_AlC and Mn-doped (V,Mn)_2_AlC MAX phases. These materials demonstrate Pauli paramagnetism in the V_2_AlC sample and a mixed Pauli-Langevin paramagnetism in the Mn-doped samples. This demonstrates the ability of microwave processing to produce magnetic MAX phases through a faster, and energy-efficient route.^152^

Microwave-assisted synthesis was used to prepare Ni_1–*x*_Zn_*x*_Fe_2_O_4_ ferrite powders, which are important soft magnets used in high-frequency devices and transformers due to their high resistivity, chemical stability, and low dielectric loss. Compared to conventional routes, the microwave method drastically reduced the synthesis temperature and time, from 900 °C for 120 min in conventional methods to 680 °C for 30 min in the microwave method. This helped prevent Zn evaporation, leading to a finer and more uniform microstructure. Figure 12a and b demonstrates the increase in saturation magnetization with an increase in microwave synthesis temperature, reaching a saturation magnetization of 87.5 A·m^2^·kg^–1^ at 950 °C.^150^

**Figure 12:**
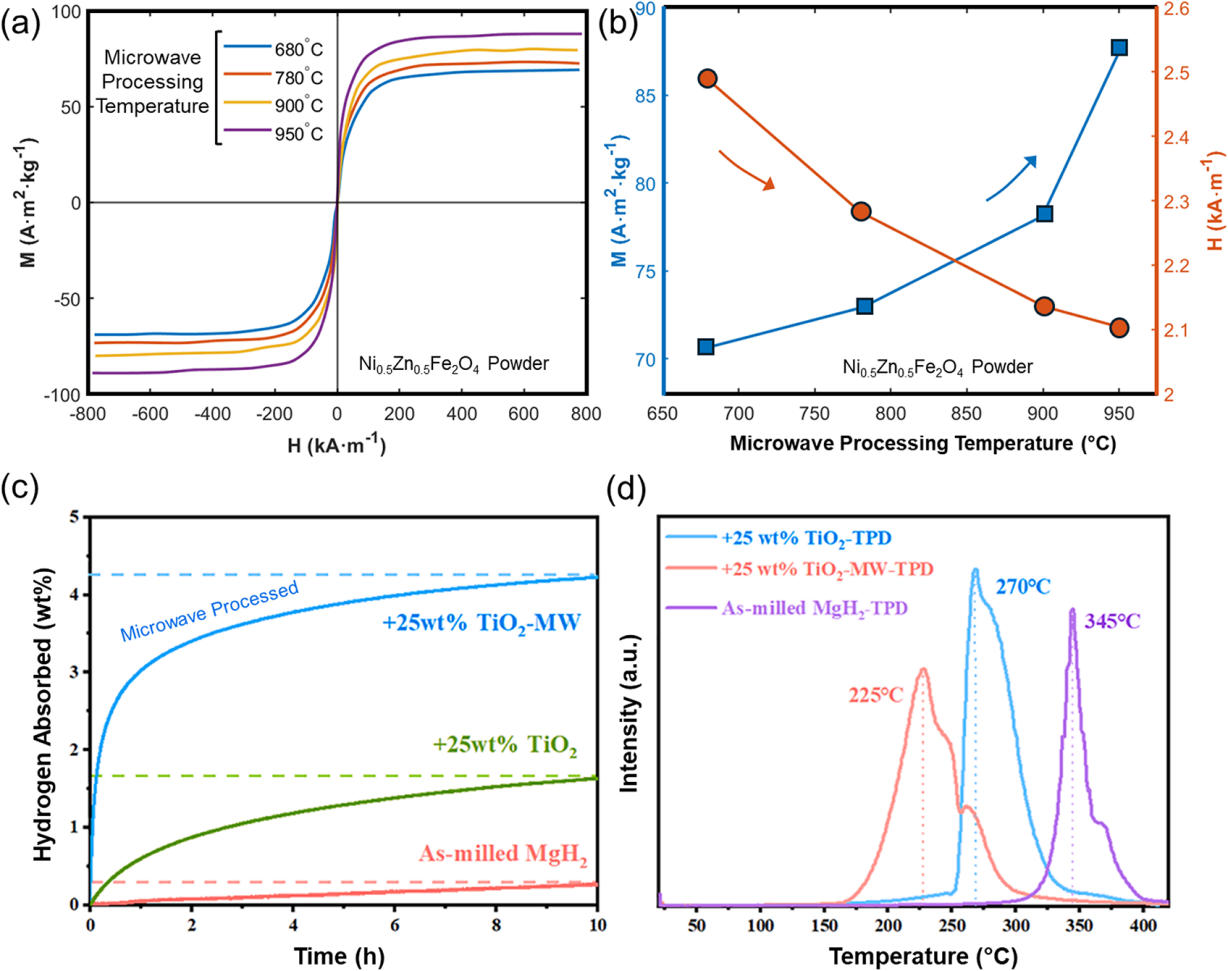
(a) and (b) Magnetic hysteresis loops for microwave-assisted synthesized Ni_0.5_Zn_0.5_Fe_2_O_4_ powder at different temperatures. Magnetic flux density (M) enhances by temperature. Reproduced with permission.^150^ Copyright 2014 Elsevier. (c) Room-temperature hydrogen absorption behavior of different MgH_2_ samples at 1 bar H_2_. Microwave-assisted processing of MgH_2_–TiO_2_ composites enhances hydrogen desorption at lower temperatures and promotes reabsorption at room temperature. (d) Temperature-programmed desorption system equipped with a mass spectrometer (TPD-MS) profiles of rehydrogenated MgH_2_, + 25 wt % TiO_2_, and +25 wt % TiO_2_-MW composites under Ar flow at a heating rate of 2 °C min^–1^. Reproduced with permission.^154^ Copyright 2022 Elsevier.

Microwave double-sintering was used to produce single-phase spinel Ni_0.2_Mg_0.8‑x_Zn_*x*_Fe_2_O_4_ (x = 0.2–0.8) NiMgZn ferrites. These materials exhibited an increase in lattice parameter, crystallite, and grain sizes with a corresponding increase in Zn content. However, the increase in Zn content also reduced both the activation energy and the Curie temperature. The microwave-sintered ferrites show low coercivity with a tunable saturation magnetization that changes composition, and they exhibit negligible remanent losses.^151^

Microwave processing of magnetic materials controls the elemental composition, phase purity, and microstructure, which, in turn, allows for the precise engineering of their magnetic properties. This technique directly addresses challenges in the field by preventing the loss of volatile elements and enabling the tuning of properties, such as saturation magnetization by changing process parameters. Ultimately, microwave processing accelerates the development of tunable magnetic materials for various applications.

### 5.6 Microwave Processing of Carbonaceous Materials

Carbon-based materials are widely used in high-performance applications, from energy storage to electromagnetic interference (EMI) shielding and advanced composites. Microwave processing addresses challenges such as preventing the degradation of metastable phases like diamond and tailoring the morphology of nanomaterials by enabling an energy-efficient, scalable, and sustainable synthesis of these materials by allowing for precise control over morphological development and enhanced phase transformation.

An important example lies in the synthesis of Diamond/Cobalt composites, which are primarily used as cutting and drilling tools for hard materials. These composites are difficult to synthesize using traditional methods because diamonds can disintegrate due to graphitization at temperatures above 900 °C. Microwave sintering offers a distinct advantage by providing rapid heating at much lower temperatures. This process helps maintain the integrity of the diamond particles by reducing the exposure to heat, thereby maintaining the metastable diamond phase.^153^

Microwave synthesis has also significantly advanced the production of nanomaterials. The thermal shock method has been used to synthesize Graphene Oxide (RGO) by rapidly heating RGO to over 1600 Kin just 100 ms, followed by an immediate quench. This results in nanoparticles with a small size and uniform distribution.^129^ Unlike conventional wet processes, microwave processing also minimizes particle agglomeration.

Microwave assisted plasma synthesis has been employed to produce few-layer graphene via the dissociation of methane in a high-temperature plasma environment, followed by recombination of reactive species to form graphitic structures. The rapid reaction kinetics enable the formation of highly pure graphene with controlled layer thickness and low defect density. Unlike conventional methods, this approach facilitates catalyst-free, scalable production of graphene.^134^

Therefore, microwave processing offers a powerful solution for addressing challenges in the synthesis of carbon-based materials. By utilizing rapid, low-temperature heating, this technique maintains the metastable phase of diamond and enables the high-yield production of nanomaterials like graphene. This makes microwave processing a superior technique for developing advanced carbonaceous materials for advanced applications.

### 5.7 Microwave Processing for Hydrogen Production and Storage

Hydrogen is a promising clean energy carrier due to its high energy density and minimal carbon emissions. However, conventional storage methods, such as compression and liquefaction, are energy-intensive and pose significant safety risks. Furthermore, materials developed using conventional methods often exhibit slow kinetics and require time-consuming production processes. Microwave processing directly addresses these issues by rapid and energy efficiently synthesizing storage materials with tunable chemistries, which can ultimately lead to improved energy capture and release efficiencies.

Liquid organic hydrogen carriers (LOHCs) offer a safe and efficient solution for hydrogen storage. These materials have a high storage capacity and are compatible with existing fuel infrastructure. They are also easier to handle and more scalable than conventional methods. Microwave-assisted dehydrogenation enhances the release of hydrogen from LOHCs through a volumetric inside-out heating, improved catalyst activity, and reduced energy consumption. Compared to conventional methods, microwave synthesis yields materials with higher surface area and pore volume, a more uniform distribution of particles, greater purity, and stronger metal interactions. It also boosts reaction kinetics and energy efficiency, making it ideal for sustainable hydrogen production.^155^

Liquid organic hydrides hydrogen carriers (LOH2Cs), a subclass of LOHCs, particularly benefit from microwave exposure due to selective heating. This enhances dehydrogenation efficiency while minimizing the overall energy input.^219^

Magnesium hydride (MgH_2_) is a promising solid-state hydrogen storage material due to its high hydrogen capacity, low cost, and reversibility. However, its practical use is limited by high operating temperatures and slow kinetics. Adding dopants like TiO_2_ can overcome these challenges. Microwave-assisted processing of MgH_2_–TiO_2_ composites leads to the in situ formation of reduced TiO_2–x_ species, which acts as microwave absorbers and create hot spots. This enhances hydrogen desorption at lower temperatures and promotes reabsorption at room temperature. Figure 12c and d demonstrate that microwave irradiation enables rapid room-temperature hydrogen uptake (4.25% in 10 h) and significantly lower the dehydrogenation peak temperature from 270 to 225 °C for TiO_2_-doped and 345 °C for pure MgH_2_, demonstrating superior kinetics and hydrogen storage performance. Compared to conventional methods, microwave irradiation improves particle dispersion, promotes titanium reduction, and refines Mg grain size, resulting in faster kinetics and hydrogen uptake, making it viable for efficient solid-state hydrogen storage.^154^

Plastics, which are hydrogen-rich waste materials biomass, which is a renewable feedstock, and low-carbon alcohols like methanol and ethanol can also be used for hydrogen generation. Microwave-assisted processing enables efficient hydrogen release from these materials by enhancing catalytical activation, rapid volumetric heating and reduced energy consumption, thereby improving hydrogen selectivity, yield and lowering the operating temperatures, making it ideal for sustainable and scalable hydrogen production.^156^

Microwave processing can be essential in furthering hydrogen economy. By accelerating dehydrogenation kinetics and by overcoming kinetic barriers, this technology addresses some of the most critical challenges in hydrogen storage. These abilities demonstrate that microwaves are essential for developing safe and efficient hydrogen storage and supply infrastructure.

### 5.8 Microwave Processing of Metals and Alloys

Due to their high electrical conductivity, the interaction of microwaves with metals, alloys, and metallic powders is unique. Bulk metals are poor absorbers of microwave because of the skin effect. Under the influence of an oscillating electric field, free electrons in the metal are confined near the surface of the material.^220^ This occurs because, according to Lenz’s law, the induced currents generate opposing magnetic fields at high frequencies. This opposing field cancels out the field inside the conductor with increasing depth.^221^ As a result, current density and energy absorption rapidly decrease with depth. Consequently, bulk metals only experience surface heating, making them inefficient for microwave heating.^27^

Interestingly, metallic powders exhibit different behaviors. Their reduced particle size and increased surface-to-volume ratio facilitates improved coupling with the microwave fields. A key factor is the presence of oxide layers and surface imperfections that act as dielectric shells between the metallic cores. This allows microwave energy to penetrate the surrounding regions, inducing volumetric heating.^222^

In metallic powders irradiated by microwave, ponderomotive forces are observed on charged particles in inhomogeneous electromagnetic fields. These strong forces enhance mass transport and can even separate the constituent phases, particularly near interfaces. This dissolution occurs in the bulk of the material and at temperatures below the melting point.^110^

Several research have shown the advantages of microwave processing for the sintering of metals and alloys.^190^ For example, compared to conventional sintering techniques, this technique can lead to improved strength and ductility in materials like copper-steel alloys.^157^ Microwave processing is particularly beneficial for refractory metals such as tungsten, molybdenum, and rhenium. Conventional techniques require extremely high temperatures and long soaking times to achieve sufficient density, which often leads to undesirable grain growth that degrades the mechanical properties of the materials. Microwave processing, however, enables sintering at lower temperatures and in less time. This rapid heating prevents excessive grain growth, resulting in nanostructured materials with over 95% density.^158,223^ Similar improvements have been reported in other metals. For example, microwave-sintered titanium, magnesium, aluminum, and bronze have shown higher hardness and increased tensile strength compared to materials processed with conventional methods.^159–162^ Figure 9d and e shows different microwave-sintered metallic samples.^190^ Recent research has shown that oxide films on copper powder particles can facilitate microwave absorption through magnetic-type losses in the initial stages of sintering. These mechanisms, which are not present in conventional sintering, are followed by percolation-assisted dielectric transitions. Dielectric response shows a sharp percolation behavior, where the imaginary component of permittivity rises sharply due to oxide decomposition, while real part peaks and then decreases as conductive pathways form. This accelerates localized heating and enhances current flow, promoting rapid densification. Similarly, the effective magnetic permeability remains below 0.1, consistent with apparent diamagnetism caused by eddy currents in response to the microwave field, contributing to uniform energy deposition. This selective, volumetric energy deposition drives the decomposition of oxides and promotes early stage neck formation, significantly altering sintering kinetics and reducing overall energy consumption.^224^

Microwave synthesis has also been used to produce copper nanostructures without the need for protecting or stabilizing agents. For example, researchers created copper nanowires with high aspect ratios of approximately 300 using CuCl_2_, ascorbic acid, and hexadecylamine in water. This method requires no surfactants or reducing atmosphere. The rapid, directional energy input from microwave heating promotes anisotropic growth and crystalline ordering, which is often difficult to achieve with conventional thermal routes.^225^

By leveraging the unique field-metal particle interaction and overcoming the challenges of the skin effect, microwave processing is capable of synthesizing refractory materials and advanced alloys with superior mechanical properties. These capabilities establish microwave processing as an essential tool for fabricating advanced alloys with superior mechanical properties for demanding structural applications.

### 5.9 Microwave Processing of Composites

Alumina-Zirconia (Al_2_O_3_–ZrO_2_) composites are important structural ceramics used in harsh environments because of their exceptional hardness and fracture toughness. Microwave sintering of these composites achieved up to 87.5% theoretical density without any isothermal holds, even at heating rates as high as 200 °C/min. Unlike conventional sintering, which requires extended holding times and higher temperatures, densification with microwave is rapid and occurs at lower temperatures. This process also helps with the formation of a homogeneous microstructure with minimal grain coarsening.^163^

WC-Co composites are important for wear-resistant applications in cutting, drilling, and tooling industries. Figure 13a–i shows the SEM micrographs of different WC-Co composites. Microwave sintering has been used to process WC-Co samples with varying particle sizes at 1000–1400 °C, which is significantly lower than the conventional temperatures. These micrographs show that there is poor bonding in samples processed at 1000 °C, but as temperatures increased to 1250 and 1400 °C, the bonding improved. Full densification is achieved in a much shorter time, with the resulting materials exhibiting superior hardness and fracture toughness of 1800 HV and $16\frac{\mathrm{MPa}}{\surd m}$ , respectively, compared to 1350 HV and $4.9\frac{\mathrm{MPa}}{\surd m}$ for samples processed with traditional methods.^164^

**Figure 13:**
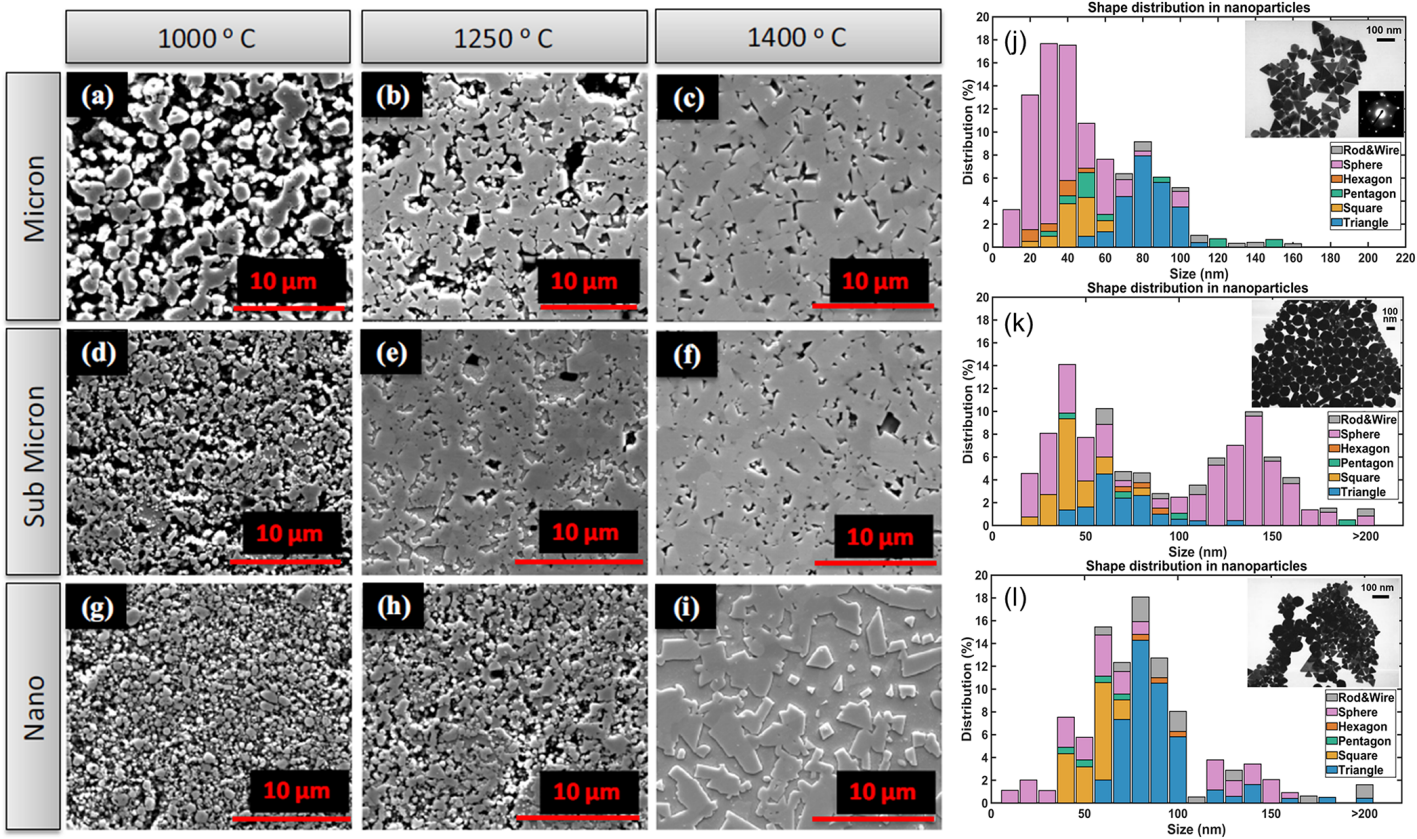
WC–Co composites at different particle size scales and sintering temperatures: micron (a–c), submicron (d–f), and nano (g–i) sintered at 1000 °C, 1250 °C, and 1400 °C, respectively. Reproduced with permission.^164^ Copyright 2019 Springer Nature. TEM images and particle distribution and shape analysis of Au nanostructures made by (j) continuous microwave heating for 2 min, (k) oil-bath heating for 19 min, and (l) pulse microwave heating for 19 min. Reproduced with permission.^226^ Copyright 2004 John Wiley and Sons.

Microwave processing has also been used to produce Ti and Ti alloys, which are used for structural, biomedical, and shape-memory application.^227^ Microwave heating of TiO_2_ and Fe_3_O_4_ rapidly absorbed energy and reached high temperatures quickly due to selective oxide coupling.^228^ Microwaves were also used to sinter Ti_6_Al_4_V with MWCNTs to produce Ti alloy/TiC porous composites.^229^ Additionally, porous NiTi shape memory alloys were synthesized by microwave processing at relatively low sintering temperatures.^230^

Microwave processing has been employed in the fabrication of W/Cu functionally graded materials (FGMs), where variations in composition and properties was achieved through selective heating. This approach generates thermal gradients and enables the formation of varying porosity and composition across the sample.^165^

Microwave-activated combustion synthesis was employed to fabricate Ti–B FGMs through controlled propagation of combustion reactions. The use of microwave enabled in situ activation of the combustion process, facilitating the formation of graded microstructures throughout the sample.^231^

Microwaves were also used to synthesize Mg-based nanocomposites with enhanced mechanical and thermal properties. Mg/Cu composites were synthesized by microwave-assisted rapid sintering with near-theoretical density.^232^ Mg/Y_2_O_3_ nanocomposites were also synthesized with microwave assistance. The resultant composites exhibited uniform nanoparticle dispersion with a reduced coefficient of thermal expansion and improved mechanical properties.^233^ Additionally, microwaves were used to synthesize Mg/BN nanocomposites using a two-directional sintering process. The nano BN particles improved the hardness and compressive yield strength and reduced tension-compression by weakening the basal structure of Mg.^234^

Due to their high-temperature strength, corrosion resistance, and lightweight nature, Ceramic Matrix Composites (CMCs) are valuable for use in extreme environments. The CEM-WAVE project, an EU-funded initiative, is developing an innovative microwave-based production process for CMCs. The use of microwave-assisted Chemical Vapor Infiltration (CVI) contains production costs, making them economically viable and sustainable alternatives. These CMCs also demonstrate improved thermo-mechanical properties, a longer service life in corrosive settings, and provide energy savings in steelmaking applications.^235^

There are, however, challenges associated with dynamic dielectric behavior during the microwave processing of composites. In multiphase systems, each constituent exhibits a different permittivity and loss factor, which evolves with changes in temperature during heating. This can lead to nonuniform absorption of energy and the formation of localized hot spots due to selective heating of individual phases. As the dielectric losses increase with temperature, a nonlinear feedback mechanism can develop, where enhanced microwave absorption accelerates heating, leading to thermal runaway and unstable processing conditions. This behavior has been shown to result in multiple steady states and abrupt transitions in temperature response. Together, these effects make process control and uniform microstructural evolution more challenging.^5,236^ These examples prove how microwaves can be used to achieve high densification at lower temperatures while simultaneously creating fine and homogeneous microstructures in composites. This capability enables the development of high-performance composites with superior mechanical properties for structural, biomedical and high-temperature applications.

### 5.10 Microwave Processing of Catalysts

Catalysts are crucial for accelerating chemical reactions by lowering the activation energy required for reactions to proceed. In recent years, nanostructured catalysts, particularly metallic ones, have gained significant attention due to their high surface area, tunable electronic properties, and enhanced catalytic activity. Microwave-assisted synthesis has emerged as a promising technique for synthesizing these catalysts.

A two-phase water–toluene reaction system has been used to synthesize gold nanoparticles. Microwave energy drives the reduction of metal ions, leading to controlled nucleation and tunable interparticle spacing.^237^ Furthermore, microwave dielectric heating enables one-pot synthesis of monometallic and bimetallic nanostructures of gold, platinum, and gold/palladium with diverse morphologies and high crystallinity.^226^

Figure 13j–l demonstrates the TEM images and histogram distribution of the various gold (Au) morphologies formed during continuous wave microwave heating (for 2 min), oil-bath heating (for 19 min), and pulsed microwave heating (for 19 min), respectively. Accordingly, continuous microwave processing produces smaller Au nanoparticles with dominant sphere shapes, while pulsed microwave heating produces predominantly triangular shapes. This technique can even produce anisotropic shapes, such as triangular and square nanoplatelets of gold,^166^ along with particles that have tight size distributions and enhanced crystallinity.^167^

The benefits of microwave synthesis extend to other materials as well. For instance, silver nanoparticles with notable properties have been synthesized in a matter of seconds. In one study, monodispersed polycrystalline silver nanoparticles, approximately 10–15 nm in size, were produced in ethanol in just five seconds, exhibiting strong plasmon resonance and fluorescence.^238^ Similarly, a green, aqueous-phase microwave route enabled the synthesis of fluorescent silver nanoclusters (∼2.0 nm) in just over a minute.^239^ These clusters demonstrated strong fluorescence and Cr^3+^ ion sensing capabilities. The rapid and uniform volumetric heating provided by microwave promotes controlled nucleation and high optical quality in both cases.

Beyond gold and silver, microwave-synthesis has also been successfully employed to synthesize nanoparticles of other materials including silver, platinum, palladium, copper, iron, cobalt, and nickel. Compared to traditional methods, microwave-assisted synthesis offers accelerated kinetics, lower processing temperatures, narrower particle size distributions and improved catalytic performance compared to traditional methods.^240–244^

Microwave can also be used to precisely control phase transformations in catalysts. Titanium dioxide (TiO_2_), a widely used photocatalytic material, exists in two key phases: anatase (more active) and rutile (more stable). Researchers have observed the anatase-to-rutile transformation of TiO_2_ under microwave heating, demonstrating a precise, real-time approach to change the phase composition for desired photocatalytic performance. By correlating Raman peak shifts, they showed that microwave heating accelerates this phase transition through higher localized temperatures, a process that is faster and has distinct kinetics compared to conventional furnace heating.^168^ Microwave-assisted synthesis has been applied to create periodic mesoporous organosilica (PMO) materials, which are used for solid acid catalysis and proton-conducting applications in fuel cells. Microwave processing of PMO materials reduced the synthesis time from over 80 h to just 12 h).^245^

Microwave synthesis has been applied to create composite catalysts, such as magnetite/reduced graphene oxide (rGO) heterostructures. This rapid and energy-efficient process produces composites with tunable shapes (such as spheres, cubes, triangle, and hexagons) and sizes along with enhanced crystallinity and morphology compared to traditional methods.^246^

A scalable microwave-assisted synthesis method was developed for highly crystalline Metal–Organic Chalcogenolate Assemblies (MOCHAs), which have emerged as promising electrocatalysts for syngas production via CO_2_ reduction. Conventionally, they were synthesized through biphasic or tarnishing methods which had low yields, long reaction time of 3 days, and use of toxic precursors, with limited scalability. Microwave-assisted processing reduced the synthesis to under 6 h, increased yield, and eliminated the need for hazardous reagents.^247^ Furthermore, microwave heating has been successfully applied to the synthesis of Metal–Organic Frameworks (MOFs), which have high surface areas and tunable properties, making them strong candidates for catalysis, gas storage, sensing and drug delivery. Microwave-assisted synthesis shortens reaction times, accelerates the kinetics of crystal nucleation and growth, and results in higher yields of desired products compared to conventional methods.^169^

Consequently, microwave processing is capable of controlling the shape, size, phase, and composition of the catalytic materials. This capability enables the precise engineering of active sites at the nanoscale level, leading to the creation of highly efficient catalysts for chemical production and energy conversion.

### 5.11 Microwave Processing of 2D Materials

Some two-dimensional (2D) metal oxides like Fe_2_O_3_, Cr_2_O_3_, CuO, ZnO and MgO are gaining attention in applications like catalysis, electronics, and energy storage due to their unique properties. A fundamental challenge lies in developing scalable synthesis method which can produce repeatable high-quality products with minimized defects, while using environmentally friendly routes that do not involve using harsh chemicals. Microwave processing has proved to demonstrate excellent solution for synthesizing and processing these materials, offering a rapid, scalable, greener and energy-efficient alternative to conventional techniques. Microwave have been used for single-step scalable synthesis of hematene, a 2D metal oxide (Figure 14a–d).^170^ Raman spectroscopy of microwave processed hematene showed high purity of phases, supporting the claim of high crystallinity and minimal defects. Microwave irradiation promotes oxide formation at localized hotspots, leading to the crystallization of high-purity, large area particles with minimal defects. The resulting particles show ferromagnetic behavior at room temperature and have a bandgap suitable for optoelectronic applications.

**Figure 14:**
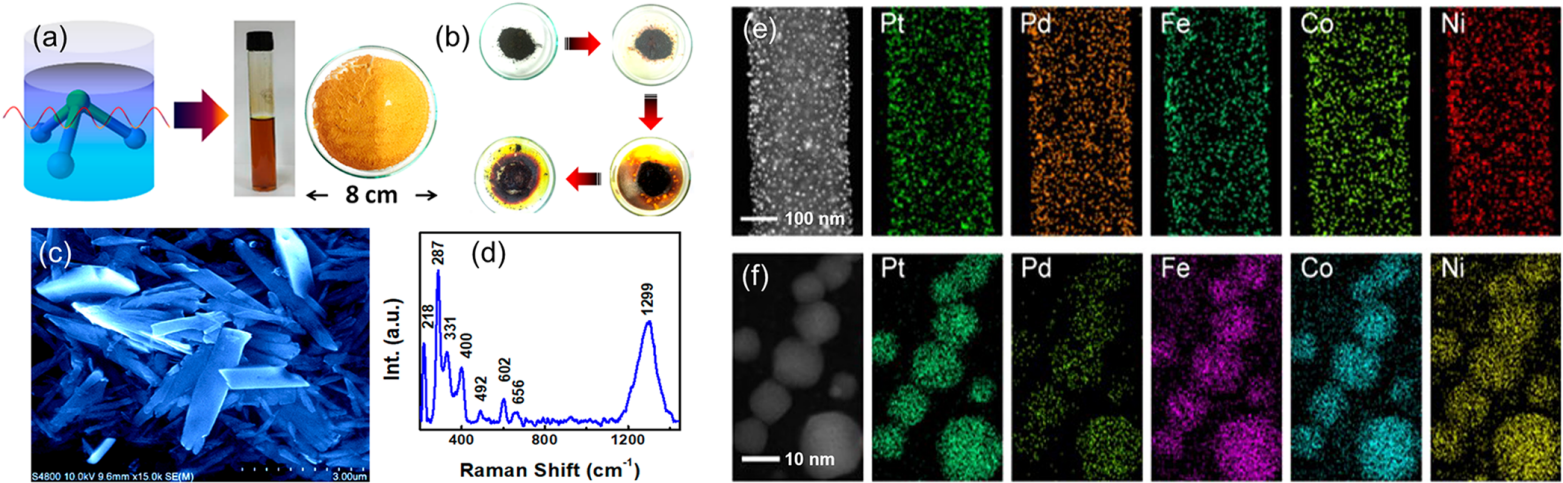
Synthesis and characterization of hematane. (a) Schematic diagram of microwave synthesis of hematane and powders after drying. (b) Actual images of the microwave synthesis process, each step after 40 s. (c) Field-emission scanning electron microscopy (FESEM) image, and (d) Raman spectrum of synthesized hematene. Reprinted (adapted) with permission:^170^ Chahal, S.; Kauzlarich, S. M.; Kumar, P. Microwave Synthesis of Hematene and Other Two-Dimensional Oxides. ACS Mater. Lett. 2021, 3 (5), 631–640. Copyright 2021 American Chemical Society. HAADF-STEM image and corresponding EDS elemental mapping of Pt, Pd, Fe, Co, and Ni nanoparticles synthesized by microwave irradiation on (e) carbon nanofibers (CNFs) and (f) carbonized wood (c-wood). Reprinted (adapted) with permission:^248^ Qiao, H.; Saray, M. T.; Wang, X.; Xu, S.; Chen, G.; Huang, Z.; et al. Scalable Synthesis of High Entropy Alloy Nanoparticles by Microwave Heating. ACS Nano 2021, 15 (9), 14928–14937. Copyright 2021 American Chemical Society.

Similarly, microwave-assisted synthesis has been applied to fabricate highly porous, single-layer graphene for use in high-performance supercapacitor electrodes. Microwave enables rapid and uniform heating during covalent functionalization of graphite with Polyethylene glycol (PEG), improving their exfoliation efficiency and dispersibility in solvents. The microwave-assisted process accelerates the electrophilic addition reaction, weakens interlayer van der Waals forces, and facilitates large-scale production of defect-minimized graphene sheets. Compared to conventional process, the microwave-assisted approach is faster, more energy-efficient, and yields over 80% single-layer graphene with a high surface area. The resulting porous morphology enhances the capacitance and rate capability for energy storage applications.^249^

Graphitic carbon nitride (g-C_3_N_4_) is a metal-free photocatalyst used for degrading organic pollutants and for solar energy conversion. Microwave synthesis of these particles from melamine and graphite leverages the graphite as a microwave absorber, which rapidly generates localized heating to enable ultrafast polycondensation. Compared to conventional methods, microwave synthesis produces g-C_3_N_4_ with higher crystallinity, larger surface areas, and unique nanoarchitectures, all of which enhance charge separation and photocatalytic activity. Microwave processing also significantly reduces reaction times from hours to minutes, improves thermal efficiency, and eliminates the need for inert gases or catalysts, making it a low-cost, and energy efficient alternative to conventional routes.^250^

LaMn_*x*_Ni_1–*x*_O_3_ perovskite solid solutions are electrocatalysts for the urea oxidation reaction (UOR). The UOR is a promising alternative to the oxygen evolution reaction (OER) in water splitting because it requires a lower voltage. Microwave synthesis provides rapid, high-energy, and transient heating, enabling precise control, and single-step creation of 2D porous nickel-rich structures. This method enhances conductivity, increased the exposure of active sites, and optimizes the electronic configuration, resulting in a superior UOR activity. Compared to conventional processing, microwave processing significantly reduces reaction time, prevents byproduct formation, and preserves the delicate 2D structures, leading to higher efficiency and stability in catalytic performance.^171^

Microwave processing has also been applied to MXenes, a class of 2D metal carbides and nitrides used in various applications including energy storage, sensing, and catalysis. By enabling uniform and volumetric energy transfer, microwave processing drastically reduces the etching time required for delamination from several hours to just 30 min. This method offers a greener, faster, and more scalable synthesis by efficiently removing aluminum without the need for hazardous hydrofluoric acid (HF) used in traditional processes.^172^

These examples demonstrate how microwave processing provides rapid, selective and volumetric heating without the need for harsh chemicals. The resulting production of high-quality and defect-free monolayers proves the unique potential of microwaves to form 2D materials with improved properties for applications in electronics, catalysis, and energy storage devices.

### 5.12 Microwave Processing of High-Entropy Alloys

High-Entropy alloys (HEAs) are a new class of materials that combine multiple principal elements, often more than 5, in near-equiatomic ratios, leading to an increase in the possible compositional space. They have gained a significant attention for having exceptional mechanical strength, high corrosion, and wear resistance, and remarkable thermal stability, making them a promising candidate for aerospace, energy, and defense applications.^251^ Through rapid, uniform, and energy-efficient heating, microwave processing enables the synthesis of HEAs. Unlike conventional furnaces, microwaves overcome enthalpic barriers in seconds, providing a faster, cleaner, and more scalable pathway to develop novel HEAs with improved properties.

The FeCoNiCrAl system is important due to its balance of high strength, oxidation resistance and thermal stability, making it an attractive material for mechanical parts and high-temperature environments. Traditional melting methods often lead to segregation, porosity, and long sintering cycles, while ball milling can take up to 60 h to form a solid solution. Microwave heating at 2.45 or 5.8 GHz was used to ignite exothermal reactions in pressed metal powder mixtures, rapidly stabilizing the BCC solid solution in about 38 s. Although some porosity was retained, microwaves required much lower energy input and preserved near-net-shape geometries compared to conventional furnace sintering or arc melting. This demonstrated microwaves to be a faster, cleaner, and a more energy-efficient route to synthesize HEA materials.^173^

HEAs supported by graphene oxide offer tunable electronic states and enhanced catalytic activity for electrochemical applications such as oxygen reduction and oxygen evolution reactions. However, conventional reduction routes face problems such as phase segregation, uneven nucleation, and long reaction times, which limits scalability. To address this, microwave-assisted synthesis techniques were used to coreduce multiple metal precursors onto graphene sheets, taking advantage of volumetric heating to achieve uniform distribution and prevent phase separation. The resulting HEA nanocatalysts exhibited higher stability and superior electrochemical activity compared to conventional methods, demonstrating ability of microwaves to create functional HEAs with controlled nanostructures and enhanced catalytic performance.^174^

Mesoporous HEAs combined with solid-solution stability of multicomponent alloys with a high surface area and tunable porosity, making them desirable for catalysis and energy conversion. Conventional template-assisted or thermal routes often fail when coreducing five or more metals due to differences in redox potentials and diffusion kinetics, leading to core–shell structures instead of homogeneous alloys. Microwave-assisted methods were used to synthesize mesoporous RhAgCuPdPt HEA nanoparticles and Au-core/HEA-shell structures in just minutes. Microwave heating minimized reduction mismatches by rapidly and uniformly heating the reaction mixtures, producing homogeneous alloy nanoparticles with uniform mesoporosity.^175^

Microwave and microwave-assisted synthesis has been extended to synthesize a broad range of more HEAs such as AlCrTiTaMo,^252^ FeCoNi_1·5_CuB_0·5_Y_0.2_,^253^ PtPdFeCoNi,^248^ (Hf, Zr, Ti, Ta, Mo)­B_2_ high entropy diborides,^254^ (MgCoNiCuZn)O HEA oxide ceramics,^255^ FeCoNiCuAl, FeCrNiTiAl, and FeCoCrNiAl_2.5_.^256^ Compared to conventional synthesis methods, microwaves enabled faster reaction times, uniform energy deposition, and refined microstructural control. Figure 14e and f demonstrate the high-angle annular dark field scanning transmission electron microscopy (HAADF-STEM) image and corresponding EDS elemental mapping for Pt, Pd, Co, Fe, and Ni grown on both CNF and c-wood supports.^248^ The uniform elemental distribution verifies that microwave irradiation drove the formation of true high-entropy alloys.

By providing rapid, nonequilibrium heating, microwave processing surpasses the kinetic and thermodynamic barriers that typically create phase instability in multicomponent systems. This forces the formation of homogeneous and single-phase solid solutions, ranging from bulk alloys to complex nanostructures. These capabilities unlock a pathway for developing new high-entropy alloys with exceptional mechanical and thermal properties.

### 5.13 Microwave Processing of Organic Compounds and Polymers

Interaction of organic materials with microwave primarily occurs due to the presence of polar molecules, mainly water. Water enables microwave energy absorption through dipolar rotation and ionic conduction, which facilitates rapid, uniform and volumetric heating. These mechanisms allow for the efficient processing of naturally derived substances, biopolymers, biomass, and pharmaceuticals through rapid internal heating.

At the microscopic scale, microwave interaction in biological and organic matter is governed by a combination of dipolar polarization, ionic conduction, and interfacial polarization mechanisms.^257,258^ The heterogeneous composition of these materials, which include water, salts, carbohydrates, lipids, and proteins, creates multiple dielectric pathways for microwave energy conversion. Dipolar rotation of water molecules dominates the energy absorption, while dissolved ions such as Na^+^ and K^+^ contribute via conduction losses.

Microwave irradiation also enhances the kinetics of several chemical and biochemical transformations commonly used in organic material processing. These advantages stem from the ability of microwave to deliver energy quickly to the molecules, resulting in localized superheating and efficient dielectric heating. This enables a controlled thermal environments even in solvent-minimized or solvent-free systems.^259^ Organic synthesis benefits from these nonconventional heating methods by improving yields with reduced side reactions.^260^

Microwave-assisted extraction (MAE) has proven highly effective for extracting bioactive compounds from plants. For example, MAE was used to extract antioxidants, antimicrobials, and therapeutic compounds from peppermint (*Mentha piperita* L.). The rapid, direct heating of plant tissues enhances cell membrane electroporation and improves solvent penetration, leading to higher yields of phenolics, pigments, and antioxidants compared to conventional methods. MAE also reduces processing time, solvent consumption, and energy use while preserving volatile oils and product quality, making it a greener and more scalable alternative to conventional extraction.^176^

In polymer synthesis, microwave-assisted methods offer significant advantages. For instance, poly (ε-caprolactone), a biodegradable polymer used in biomedical applications such as resorbable sutures, drug delivery, and scaffolds in tissue engineering can be synthesized using microwave-assisted ring-opening polymerization. This method leverages the monomer’s dipolar nature for rapid volumetric heating, enabling faster temperature ramp-up and real-time process control. This allows for fine-tuning of polymerization kinetics while minimizing thermal degradation.^177^

Polystyrene is widely used in packaging, insulation, consumer goods, and as a precursor for various advanced special functions. It is typically produced by Nitroxide-Mediated Radical Polymerization (NMRP) process. Compared to conventional methods, microwave-assisted NMRP process achieves higher conversion rates in shorter timeframes and precise temperature control. It also yields polymers with narrow molecular weight distribution, improving both efficiency and product quality.^178^

Polymers of methyl acrylate and methyl methacrylate are valuable in coatings, adhesives, biomedical devices, and advanced materials requiring precise molecular weight and narrow polydispersity. Microwave-assisted reversible addition–fragmentation chain transfer (RAFT) polymerization accelerates reactions by 2.5 times compared to conventional methods while maintaining excellent control and molecular weights closer to theoretical values. Microwave-assistance also improves control over polymer architecture.^261^

Microwave is also used to synthesize complex polymers like silk-like polymers for biomedical applications such as tissue engineering, sutures, and high-performance fibers. By enabling rapid solid-phase peptide synthesis and accelerating click chemistry, this method allows for faster achievement of high molecular weights.^262^

Block and cyclic copolymers are valuable in applications such as drug delivery, nanolithography, and advanced coatings, due to their tunable architecture and functionality. They can be produced rapidly and efficiently using copper nanoparticles (CuNP) coupled with microwave irradiation. Microwave heating accelerated the Cu-catalyzed cycloaddition, achieving full conversion in as little as 10 min, without the use of inert atmospheres. The CuNP catalyst is removable, hence reducing catalyst contamination which is observed in conventional methods. This leads to faster synthesis, cleaner products, and scalability, compared to traditional approaches.^263^

Poly (9,9-dihexylfluorene)­s (PDHFs) are conjugated polymers used in optoelectronic applications due to high photoluminescence efficiency, good solubility, and charge transport properties. Microwave-assisted Suzuki and Yamamoto polymerizations have enabled the rapid synthesis of high-molecular weight PHDFs in minutes, compared to the 72 h required by conventional method. This results in higher yields and improved process efficiency, making it ideal for commercial production.^264^

Reduced graphene oxide-polystyrene-polymethyl methacrylate/silver nanoparticle (R-(GO-(PS–PMMA))/AgNPs) nanocomposites are designed for antimicrobial applications. Microwave irradiation enables rapid in situ reduction of GO and the formation of well-dispersed, ultrasmall AgNPs within the polymer matrix, enhancing interfacial interactions. This uniform dispersion improves thermal stability, crystallinity and antibacterial activity, while also reducing synthesis time and energy consumption compared to conventional methods.^265^

These examples show the advantages of microwave processing for organic compounds and polymers compared to conventional methods. By accelerating reaction kinetics and preserving molecular integrity, microwaves provide a more efficient, scalable, and environmentally friendly pathway to develop and synthesize organic materials and advanced polymers.

### 5.14 Microwave Processing of Biomaterials and Biomedical Devices

Microwave processing has emerged as a promising approach for synthesizing biomaterials owing to its ability to provide rapid and volumetric heating that leads to enhanced microstructural control. These attributes are crucial for biomedical applications, where material performance is strongly influenced by density, porosity, and interfacial characteristics.^266^

Microwave processing has been explored for metallic biomaterials such as stainless steels, cobalt-based alloys, and titanium and its alloys, which are commonly used in orthopedic and dental applications including artificial joints, bone plates, and dental implants due to their high strength, corrosion resistance, and biocompatibility.^179^ Microwave processing of these materials has been shown to enhance densification, refine microstructures, and improve mechanical properties, thereby resulting in the superior performance of these components.

Similarly, zirconia, a critical material for dental fixtures and implants, can be densified in just 15 min using microwave processing, in distinct contrast to the 2 h required for traditional sintering. Beyond faster processing and lower temperatures, microwave processing also results in refined grain sizes of ∼0.5 μm, which is notably smaller than the ∼0.9 μm grain sizes typically obtained with conventional methods.^180^ In another study, a complex gear-shaped zirconia component achieved ∼96% of its theoretical density using microwave sintering at 1080 °C; the samples retained their intricate geometry with only minor distortion.^181^

Microwave-assisted hydrothermal synthesis has also been demonstrated for the fabrication of magnesium phosphate-based biomaterials which possess excellent biocompatibility and biodegradability. Rapid synthesis of amorphous magnesium phosphate hierarchical nanostructures can be achieved within minutes using microwave processing, enabling precise control over the morphology and surface characteristics.^267^

Microwave sintering has been successfully applied to hydroxyapatite (HAP)^182^ and to 70Sr–Zr–HAP.xZn­(30–x)­Si composites^183^ used for biomedical applications, especially for bone repair and regeneration. This is due to their enhanced biocompatibility, osteoconductive nature, and bioactivity. The rapid and volumetric heating in microwaves yielded a higher relative density, more uniform grains, lower porosity, and higher mechanical strength in HAP, and in the 70Sr–Zr–HAP.xZn­(30–x)­Si composites. Furthermore, these composites exhibited increased hardness and enhanced bioactivity, evidenced by the formation of a thicker hydroxyapatite layer during bioactivity tests compared to conventionally processed materials.

Functionally graded HAP–Ti materials have also been fabricated using microwave processing, where gradients in composition eliminate sharp interfaces between the ceramic and metallic phases. This enables a continuous transition in microstructure and mechanical properties, which improves interfacial bonding and reduces mechanical mismatch. Consequently, this leads to enhanced strength and toughness for hard tissue replacement applications.^268^

Microwave-assisted processing has also been applied to the development of antibacterial biomaterial coatings, such as silver-doped calcium-deficient hydroxyapatite (Ag-CDHA) on Ti6Al4V substrates that are used in orthopedic and dental implants. This combination of microwave irradiation with biomimetic coating enables the formation of a single-phase CDHA with homogeneous incorporation of Ag^+^ ions within the apatite lattice. This is difficult to achieve using conventional methods. Uniform distribution of Ag^+^ ions leads to a controlled and sustained release of Ag^+^ ions, providing effective antibacterial activity. Through excellent cytocompatibility, proliferation, and preosteoblast cell attachment, these materials demonstrate their potential to improve implant performance and reducing infection-related complications.^269^

These examples demonstrate how microwave processing can control the microstructure, phase composition, and functional properties of biomaterials. The rapid and volumetric heating enhances densification, refines microstructures, and improves biomedical performance. Therefore, microwave processing provides a promising and efficient pathway for the development of advanced biomaterials and biomedical devices with enhanced structural integrity, functionality, and performance.

## 6 Unique Capabilities of Microwaves

Microwave processing is not only useful for traditional methods such as in the synthesis and sintering of materials, but it can be used to demonstrate unique capabilities for advanced and unconventional techniques. Beyond the conventional applications, microwave processing has been demonstrated to be useful for joining and bonding of metallic and nonmetallic materials, in additive manufacturing or 3D printing, and also as a powerful tool for recycling materials from complex waste streams that are difficult, time-consuming, or expensive with traditional methods. The ability of microwave to provide volumetric, rapid, and selective heating opens new pathways for processing materials with high energy efficiency and reduced reaction times. This versatile nature positions microwave processing not just as an alternative, but often as a superior method that can tackle challenges which conventional approaches struggle to overcome.

### 6.1 Microwave Processing for Joining

Joining of dissimilar materials, such as metal–metal, metal-ceramic, ceramic-glass, and metal-glass, with different thermal expansion coefficients poses significant challenges in conventional manufacturing. Traditional welding and brazing techniques are often unable to join a broad range of materials together; furthermore, they create heat-affected zones with induced thermal stresses that can degrade the properties of the joint interface. To tackle these challenges, microwave processing emerges as an innovative technique for joining various ferrous and nonferrous metals, as well as nonmetals. Unlike conventional methods that rely on external heat sources, microwave processing uses rapid, volumetric, and selective heating to directly interact with materials. This allows for heating at the interface while minimizing thermal damage to the surrounding areas. The result is faster joining rates, lower residual stress, and the ability to join dissimilar materials with tailored microstructures. This approach offers energy savings and enhanced joint performance compared to traditional manufacturing.

Microwave joining has been successfully applied to mild steel plates, with the average hardness of the joints (420 ± 30 Hv) being significantly higher than that of the substrates (230 ± 10 Hv).^270^ Microwave has also been used to join bulk stainless steel 316 plates, fabricating a dense microstructure with strong bonding and epitaxial grain growth at the fusion interface. The formation of chromium carbide formation at these interfaces increased hardness values to 650 ± 40 Hv, compared to the base value of 225 ± 10 Hv.^271^ Microwave joining has even been used for thin steel sheets using a 2 kW multimode magnetron at 2.45 GHz.^272^

The technique has also been used for nonferrous metals. Bulk copper has been joined using a hybrid microwave method with copper slurry as an interlayer and charcoal as the susceptor, resulting in dense, crack-free joints with low porosity and high hardness.^273^ Inconel 718 plates have been joined with nickel powder interlayer, showing complete fusion and achieving an ultimate tensile strength of 400 MPa and elongation of 6%.^274^

Microwave joining can also bond dissimilar metals, such as SS-316 and mild steel plates, using a nickel-based interlayer and susceptor-assisted hybrid heating. This process resulted in a dense fusion with low porosity (0.58%).^275^ Microwave processing has also been shown to join nonferrous metals like copper, magnesium, and aluminum. In the microwave joining of copper, a phase transformation from Cu (311) to Cu (111) orientation and the formation of CuO were observed. This yielded a material with high ductility, though failure tests showed a strong yet partially brittle fused interface.^273^ Furthermore, microwave hybrid heating was used to join dissimilar Al6061-T6 and AZ31B Mg alloys using TiCuSi as active braze layer. This technique led to enhanced diffusion and solid solution formation at the interface, as confirmed by SEM and EDX analysis.^276^

Joining ceramics is challenging due to their inherent brittleness and difficulty in machining. Conventional joining techniques like diffusion bonding, glass-ceramic sealing, or brazing often require long exposure to high temperatures, complex tooling, and controlled atmosphere, which increase costs and can degrade material properties. Microwave joining has emerged as a promising solution for ceramics, successfully joining a variety of materials including Al_2_O_3_,^277^ Al_6_Si_2_O_13_, Si_3_N_4_,^278^ SiC,^279^ zirconia, MgAl_2_O_4_ spinel, MgF_2_,^280^ Zinc sulfide,^281^ Alumina-zirconia composites,^282^ Bi–Pb–Sr–Ca–Cu-O ceramic superconductors.^283^ By using microwave-absorbing interlayers like SiC, alumina composites with SiC is fabricated, with reduced processing time and minimized thermal damage, offering a superior alternative to traditional methods.^284,285^

With the discovery of advanced materials, the joining of dissimilar materials is crucial, and the techniques need to be updated since traditional methods are generally limited. The examples above demonstrate the superiority of microwave joining over conventional techniques. By providing precise and localized energy deposition, core challenges such as thermal damage and residual stress are effectively addressed. Microwave processing is able bond a variety of similar and dissimilar metals and ceramics, thereby unlocking complex multimaterial interfaces with tailored properties and superior performance.

### 6.2 Microwave Processing for 3D Printing

3D printing has been a transformative manufacturing technique for fabricating complex and customizable structures with reduced material waste. However, in traditional extrusion processes, where materials are forced through a nozzle and deposited layer by layer, challenges in shape retention and structural stability exist during deposition. Microwave processing offers a unique solution by enabling in situ curing and phase transformation during the extrusion process.

Microwave-assisted embedded 3D printing (EMB3D) has been demonstrated as a powerful route to process architected ceramics with programmable geometries and compositions. Conventional ceramic printing faces issues such as difficulty in particle loading, shrinkage defects, or constraints in geometries. EMB3D uses colloidal inks in a silicone support matrix that are then rapidly cured by microwave-activated polymerization as illustrated in Figure 15a. The use of microwaves enabled uniform and selective curing of the filaments, preventing cracking and minimizing thermal gradients. Microwave heating also enabled near-theoretical densities (∼97%) after sintering.^286^ Figure 15b demonstrate optical images of additively manufactured and microwave sintered components: a stochastic sphere, a multimaterial lattice composed of pure (horizontal), and cobalt-doped (vertical) yttria-stabilized zirconia struts, and interpenetrating ceramic links patterned as a close chain. Furthermore, this technique can also be used to create functionally graded components as shown in Figure 15c, where a gradual transition from Ni-doped Al_2_O_3_ (in blue) to pure YSZ (in red) is demonstrated. The ability to create multimaterial architectures with spatially controlled gradients highlights the potential of this technique to enable the development of graded ceramic systems.

**Figure 15:**
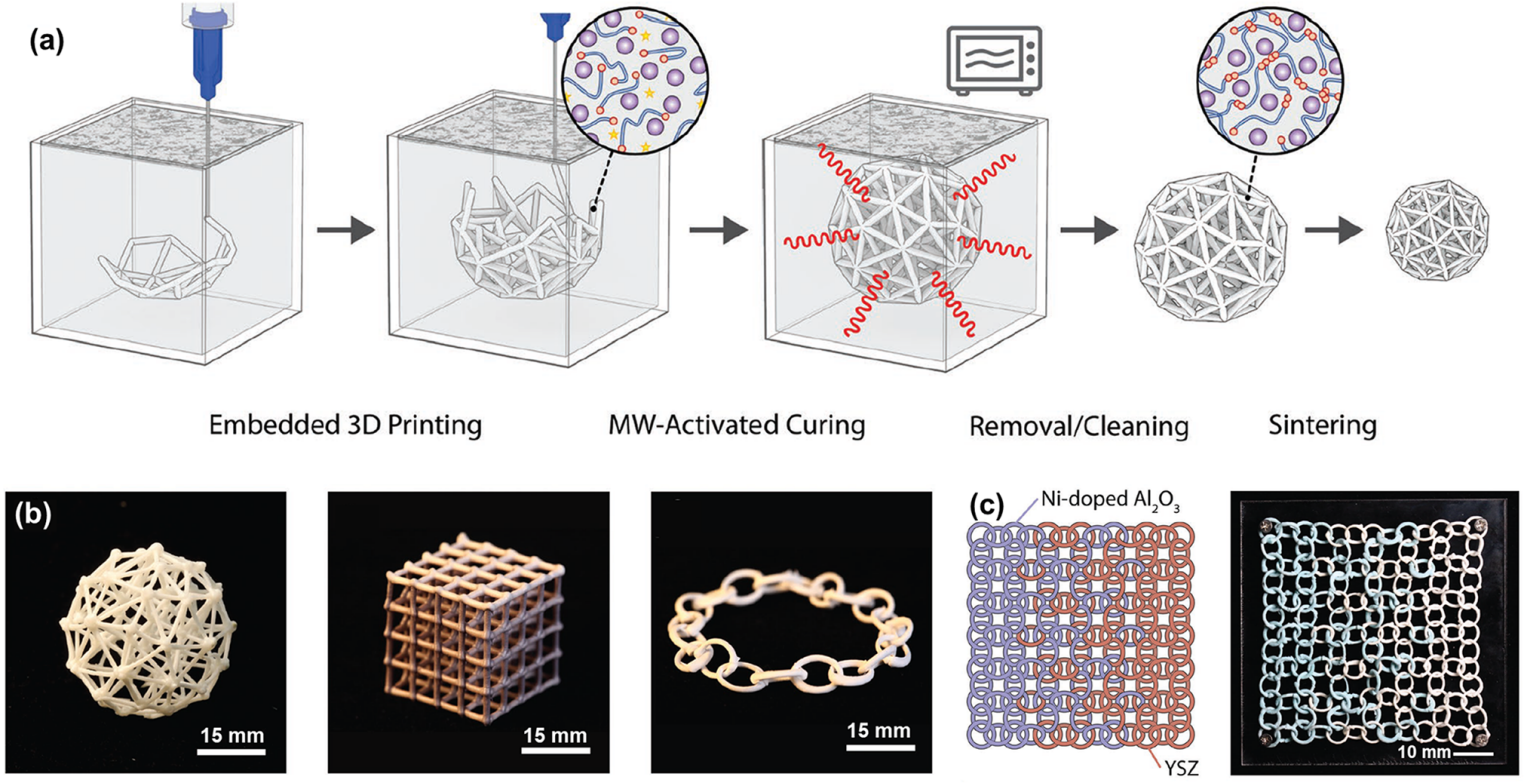
(a) Schematic of the microwave assisted embedded 3D printing (EMB3D) process. (b) Optical images of microwave sintered of additively manufactured ceramic components: (left) stochastic sphere, (center) multimaterial lattice composed of pure and cobalt-doped yttria-stabilized zirconia struts, and (right) interpenetrating ceramic links patterned as a close chain, (c) A compositionally graded chainmail weave composed of Ni-doped Al_2_O_3_ (blue) and YSZ (red) links. Reprinted with permission.^286^ Copyright 2023 John Wiley and Sons.

Due to its chemical similarity to bones, osteoconductivity, and the ability to support ingrowth of tissues, tricalcium phosphate (TCP) was used as a bioresorbable ceramic scaffold material. 3D printed TCP scaffolds with controlled interconnected porosity were fabricated and subsequently sintered using microwaves. MW sintering enabled rapid and volumetric heating which led to higher densification and a refined microstructure, with improved compressive strength compared to traditional sintering. Microwave sintering also minimized grain growth, reduced porosity, and achieved superior mechanical performance while maintain bioactivity.^287^

Microwaves have been demonstrated to 3D print epoxy resins, which are widely used as structural material and various industries. Conventional methods such as direct ink writing (DIW) face challenges like low viscosity and slow curing, which lead to poor shape retention and spreading of the inks. MW heating enabled synchronous extrusion and volumetric curing, thereby rapidly increasing the viscosity and storage modulus to stabilize the ink during deposition. Microwaves provide uniform, rapid, and energy-efficient curing, without the need for expensive photo initiators. This results in high-quality, multilayer epoxy prints with excellent mechanical properties and improved shape fidelity.^288^

Microwave-assisted 3D printing has also shown promise in fabricating food materials such as Surimi and globulin. In Surimi, microwaves enabled rapid in situ gelation after extrusion, improving structural fidelity, while in globulin, controlled predenaturation with l-cysteine allowed for rapid gelling into a stable material suitable for high-resolution 3D-printed structures.^289^

Therefore, microwave assisted curing shows indispensable capabilities for additive manufacturing. Through rapid, volumetric, and in situ curing, microwaves enable accelerated pathways for 3D printing and setting the components in real-time, facilitating the fabrication of complex and functionally graded components for various applications.

### 6.3 Microwave-Assisted Melting

Melting is a fundamental material manufacturing process that enables alloying and casting. Conventionally, melting is performed using resistance furnaces, induction furnaces, and other thermal methods. However, these approaches often require prolonged processing times and higher energy input compared to microwave-based systems.^290,291^ Microwave-assisted melting offers a promising alternative by enabling rapid and volumetric heating within materials. This results in significantly accelerated heating rates once sufficient microwave coupling is achieved,^290^ and in some cases reduced overall processing time and energy consumption under controlled conditions.^291^ This behavior arises from temperature-dependent increases in dielectric and conductive losses, along with nonlinear heating effects that enhance microwave absorption at elevated temperatures.^292,293^ In addition, selective coupling of microwaves with specific phases or constituents can initiate localized heating, enabling efficient melting and improved processing kinetics in heterogeneous systems.^294,295^ Microwaves have been used to melt metals such as tin, lead, aluminum and copper using hybrid heating approaches. SiC susceptors were used to provide initial heating and enable subsequent microwave coupling with the metals. As the temperature increases, microwave absorption improves, leading to rapid heating and melting, thereby significantly reducing processing times compared to conventional methods.^290^

Microwaves have also been used for the melting and casting of metal-ceramic composites, such as SiC reinforced nickel-based matrix powders. Microwave hybrid heating involving charcoal susceptors is used to initiate the heating and promote microwave-composite coupling. As the temperature increases, both the ceramic reinforcement and the metal powders begin to couple with the microwave field, resulting in bidirectional heating and rapid temperature increase, which enables melting and casting. This process facilitates a uniform distribution of the reinforcement within the matrix, leading to equiaxed grain structures with low porosity (∼1.7%) and enhanced microhardness due to the formation of carbides and silicides during processing.^296^

Another application is the heating of refractory oxide materials such as silica. Temperatures higher than 1700 °C have been achieved through efficient microwave absorption. Since lower temperatures involve low dielectric and conduction losses, the samples are typically preheated to near melting temperatures. After this, microwave absorption becomes sufficient to sustain melting. This approach enables continuous melting at significantly lower power (400–500 W) compared to conventional systems (∼10 kW).^292^

Through mechanisms such as hybrid heating, temperature-dependent coupling and selective energy absorption, microwave-assisted melting can be achieved across a wide range of material systems, including metals, composites, and refractory oxides. In contrast to conventional techniques, where heat is externally supplied and transferred through the material, microwave processing enables internal and material-dependent energy deposition, leading to unique heating behaviors and processing characteristics. Table 7 compares microwave-assisted melting with conventional techniques such as induction melting, arc melting, and muffle furnaces.

**Table 7:** Comparison of Microwave-Assisted^290–292^ with Induction,^297,298^ Arc,^299,300^ and Muffle furnace^301,302^ Melting Techniques^a^

Parameters	Microwave Melting	Induction Melting	Arc Melting	Muffle Furnace Melting
Heating Mechanism	EMF coupling with dielectric and conductive losses assisted by hybrid heating with susceptors	Joule heating induced by alternating EMF	Localized melting through electric arc between electrode and material, generating plasma	Resistive heating through electric heating elements
Thermal Characteristics	Volumetric heating with temperature-dependent absorption	Bulk heating with strong electromagnetic stirring	Localized highly thermal gradients and melt pool formation, followed by rapid solidification	Surface dominated heating with inward heat transfer and slow temperature equilibration due to thermal gradients
Processing Time	Significantly shorter due to rapid heating	Moderate, constrained by melting temperatures and material interactions	Rapid due to extremely high arc temperatures	Long due to gradual heating and extended soaking times
Energy Efficiency	High	Moderate, potentially lower than conventional methods	Moderate to high	Low to moderate
Material Compatibility	Metals and selected ceramics depending on material properties	Conductive metallic systems	Metallic systems and reactive alloys with high melting points	Broad range of materials including metallic systems and ceramics
Key Limitation	Limited absorption in metals at lower temperatures, requiring susceptors or preheating	Reactions between crucible and material, contamination, thermal shock, and scalability challenges	Element evaporation, phase instability, thermal stress due to thermal gradients	Longer processing times, low energy efficiency, thermal gradients within the material

a EMF: Electromagnetic Field.

A promising future direction for microwave-assisted melting involves the intentional utilization of hot spots, traditionally viewed as uncontrolled and undesirable phenomena, to facilitate extremely rapid ingot melting. Overall, microwave processing shows great promise for rapid, selective, and energy-efficient melting of materials. This ability to melt materials using microwaves is strongly dependent on the material properties and processing conditions. There are challenges associated with the use of microwaves such as nonuniform heating, thermal runaway, and scale-up limitations that must be addressed to enable reliable and reproducible melting processes.

### 6.4 Microwave-Assisted Reduction of Oxides

Reduction of oxides is crucial in extractive metallurgy, catalyst preparation, and in synthesis of functional materials. However, conventional reduction methods face critical challenges, including high temperatures and costs, longer dwell times, and unsustainability. To overcome these barriers, microwave-assisted reduction offers an efficient solution. By providing rapid, volumetric, and selective heating through direct coupling with the oxides, microwaves enable enhanced reaction kinetics and lower reduction temperatures. This is achieved through the creation of localized hot spots and plasma formation, resulting in faster conversion rates and improved energy efficiency compared to conventional approaches.

Microwave irradiation can reduce graphene oxide into high-quality graphene, which is suitable for printable electronics and electrocatalyst applications. Microwave-induced deoxygenation rapidly reorders the graphene plane by removing oxygen. Unlike conventional methods which often leave residual oxygen and form structural defects, microwave reduction yields pristine reduced graphene oxide (rGO) with high carrier mobility and superior electrochemical performance. This process is rapid, reagent-free, and scalable, offering an energy-efficient route for carbon reduction.^303^

Microwave assisted carbothermic reduction has been demonstrated for reducing copper oxides and malachite concentrates. High dielectric loss in CuO and small additions of graphite promotes the in situ calcination of malachite into CuO, followed by its reduction to copper. Compared to conventional methods, the microwave processing achieved high temperatures in minutes, enabling a near-complete CuO reduction, which resulted in faster kinetics and improved efficiency.^304^

Similarly, microwave processing enabled the reduction of Copper­(I) oxide (Cu_2_O) with graphite under controlled atmospheres. The reduction of these oxides has been investigated under E-field and H-field. The E-field generated microplasmas, which accelerated oxygen removal through plasma-assisted mechanisms, while the H-field induced localized hotspots through eddy currents. The resulting reduction temperatures were 200–300 °C lower than those achieved with conventional techniques. Overall, microwave processing facilitates faster kinetics, selective coupling, and more energy-efficient processes.^305^

Microwave-assisted carbothermic reduction has also been used to reduce metal oxide-carbon pellets, such as Fe_2_O_3_ with coke/graphite. The reaction is uniform due to volumetric heating, which drives the Boudouard reaction, lowers the activation energy, and ultimately achieves a more homogeneous reduction. Consequently, microwave reduction decreases the reaction time, reduces energy consumption, and enhances the uniformity of the reaction.^306^

Mn-doped calcium titanate perovskites are used as redox carriers for thermochemical looping. In the microwave cavity, dielectric breakdown occurs, initiating a low-temperature reduction via electron transfer that promotes the formation of oxygen lattice vacancies. Furthermore, volumetric heating significantly increases the oxygen exchange kinetics, thereby lowering the overall energy demand for redox reactions.^307^

These applications demonstrate that microwave-assisted processing offers a faster and more energy-efficient technique than conventional methods and can effectively manipulate the redox chemistry of metal oxides. By leveraging hot spots, volumetric heating, and plasma formation, the thermodynamic and kinetics challenges inherent in conventional methods are mitigated, driving reactions faster and at lower temperatures. This approach establishes a more energy-efficient and scalable pathway for synthesizing functional materials.

### 6.5 Microwave-Assisted Recycling

Recycling is crucial for sustainable resource management due to the rapid depletion of resources and the rising cost of raw materials. It is also essential for reducing environmental pollution caused by the massive accumulation of waste from products with short lifespans like lithium-ion batteries, plastics, and industrial residues.^308–312^ Conventional recycling methods, such as hydrometallurgical and pyrometallurgical techniques, have significant drawbacks, including high energy consumption, lengthy processing times, complex separation steps, need for controlled atmospheres, and the use of hazardous chemicals. These issues increase operational costs and the environmental impact.^309–312^ Microwave-assisted recycling offers a viable solution by providing rapid, volumetric, and selective heating. This process accelerates reaction kinetics, reduces energy use, and often eliminates the need for additional susceptors and reductants. Furthermore, its ability to directly target microwave-absorbing phases, like metal oxides, carbon, and polar polymers, enhances process efficiency and allows for the treatment of complex, heterogeneous wastes that are difficult to recycle using the conventional methods.^308–310,312^ Microwave processing has shown promising insights into the recycling of battery materials, metallurgical residues, plastic and polymers, composite materials, and magnetic materials.

Recycling battery materials like spent coin cells (composed of lithium, manganese, and carbon),^308^ and mixed lithium-ion batteries (containing lithium, cobalt, manganese, nickel, and graphite).^310^ faces several challenges. Conventional methods require multiple separation steps, long reaction times, and significant energy for reduction. They also rely on hydrometallurgical routes and chemical leaching with expensive reagents and often need inert atmospheres.^308,310^ Microwave processing offers a superior alternative. It enables rapid, selective volumetric heating and uses recovered graphite as a reductant. This approach lowers the activation energy for reduction, eliminates the need for inert atmospheres, and achieves higher metal recovery rates in shorter amount of times.^308,310,311^

Metallurgical residues, such as hot rolling sludge, contain high levels of iron oxide, along with oil and water. Traditional recycling is limited by high oil content, slow drying, high energy consumption, and hazardous volatile organic compound (VOC) emissions. Microwave processing exploits the sludge’s metal content for strong microwave absorption, generating localized hot spots to rapidly remove moisture and volatiles within minutes. This improves the flowability of the material and reduces oxidation, making it more efficient to reuse in steel production.^309^

Microwaves have also been used in the recycling of construction and demolition of debris. Due to its high compressive strength, recycling concrete is challenging conventionally. However, by leveraging the difference in dielectric properties of aggregate and the cement, microwave pretreatment weakens the mechanical properties. The compressive strength and elastic modulus is reduced, while increasing brittleness. Upon microwave pretreatment, visible cracking is observed, confirming rapid microcrack formation by differential heating, significantly reducing the energy consumption, thereby providing a green solution for cement recycling.^313^

Microwave processing has also been used to recycle various polymers and plastics. Conventional mechanical recycling of polymers degrades their quality and is slow, while chemical methods often require additional susceptors and cannot remelt thermosets. Microwave processing overcomes these issues by directly heating the polar segments of polymers, enabling efficient depolymerization without susceptors. This allows for selective, multistage recycling of mixed plastics.^312^

Electronic waste, such as printed circuit boards (PCBs), contains valuable metals like Cu, Fe, Sn, Pb, Zn, Ni, and Al, in addition to glass and resins. Traditional recycling methods face challenges like toxic gas emissions from pyrometallurgy, corrosive chemicals from hydrometallurgy, and incomplete metal recovery during mechanical separation. Microwave processing rapidly pyrolyzes the polymeric binders, loosening the adhesion between metal and glass fiber layers. This facilitates the efficient liberation and recovery of valuable metals with fewer environmental emissions.^314^

Recycling composite materials like carbon fiber-reinforced polymers (CFRP) and glass fiber-reinforced plastics (GFRP) is challenging. Conventional recycling, methods such as mechanical grinding damages the fibers; thermal pyrolysis consumes high energy and degrades fiber quality; and chemical methods are often slow and hazardous.^315,316^ Microwave processing offers a beneficial alternative. It selectively heats conductive fibers, accelerating the decomposition of the resin matrix and enabling the recovery of fibers with minimal degradation in less time.

Recycling industrial composites such as Tetra Pak packaging, which is composed of paper, polyethylene, and aluminum, presents challenges. Mechanically separating the multilayered structure is difficult, and thermal treatments risk charring the cellulose. Microwave processing proves beneficial by selectively heating the conductive aluminum and polymers layers, allowing for rapid separation.^317^

Recycling specialty magnetic materials like Nd–Fe–B rare earth magnets is challenging due to the need for high-temperature processes for demagnetization and oxidation, which are slow and energy intensive. Microwave processing enables fast, volumetric heating for oxidation demagnetization, improving efficiency and simplifying downstream processing.^318^

By leveraging rapid and selective heating, microwave processing addresses the challenges of high energy intensity and poor selectivity inherent in conventional methods. Its ability to break down complex chemistries into specific, usable compounds allows microwave processing to enable recycling as a green and economic pathway, thereby creating a circular economy by transforming waste into valuable resources.

## 7 Limitations, Failure Modes, and Mitigation Strategies for Microwave Processing

Microwave processing is associated with several limitations and failure modes that can impact process stability, reproducibility, and scalability. These challenges arise from the coupled interactions between electromagnetic fields, thermal effects, and material properties, which can lead to nonuniform heating, microstructural inhomogeneity, and processing instabilities. Therefore, to fully utilize the potential of microwave processing, it is essential to understand these limitations and implement appropriate mitigation strategies based on material design, hybrid heating approaches, and precise process control.

### 7.1 Materials Limitations and Compatibility

The effective use of electromagnetic fields (EMFs) in advanced microwave processing is often constrained by the inherent property limitations of the materials. These challenges include inadequate microwave absorption, thermal instability, microwave reflection, and poor compatibility with EMFs. Addressing these challenges requires innovative strategies to enhance material compatibility and optimize processing outcomes.

Materials with low dielectric loss factors, such as certain ceramics and polymers, absorb very little microwave energy, leading to inefficient heating at room temperature. This limitation is typically addressed through hybrid heating approaches or the incorporation of additives to enhance microwave absorption.^21,319^ Conversely, materials that are nearly transparent to microwaves are highly valuable for auxiliary applications, such as microwave windows, support components, crucibles, and reaction vessels.

Bulk materials with high ionic or metallic conductivity tend to reflect microwaves due to the formation of surface eddy currents. This results in a shallow penetration depth (skin effect) and ineffective volumetric heating. Consequently, processing bulk metallic materials with microwaves is restricted without specific modifications.^21,320^ In systems containing conductive particles, localized intensification of the electric field can lead to electrical discharge and plasma formation (arcing). This phenomenon is influenced by parameters such as particle size, morphology, applied power, and solvent properties, which can lead to process instability and sample degradation.^321^ To mitigate arcing during microwave irradiation, strategies such as modulating the applied power, selecting appropriate solvents, and ensuring uniform particle dispersion are commonly employed.

Some materials experience rapid increases in permittivity or loss factors as temperature rises. This can lead to challenges such as uneven heating or thermal runaway, resulting in uncontrolled localized heating. This phenomenon poses a significant challenge in materials such as magnetite (Fe_3_O_4_), where dielectric properties vary drastically during microwave processing. As the dielectric loss increases, more microwave energy is absorbed, which accelerates the temperature rise and leads to hotspot formation or cracking if the process is not carefully controlled.^21,319^

To overcome challenges regarding material compatibility, several methodologies have been implemented. Hybrid heating techniques, which combine microwave energy with conventional methods such as resistive or infrared heating, facilitate a more uniform temperature distribution. These hybrid systems can preheat low-loss materials to a critical temperature where their dielectric loss increases, enabling more efficient microwave absorption.^21,319^

Embedding susceptors and nanofillers, such as amorphous or graphitized carbon, carbon nanotubes, silicon carbide, metal oxides, or metal nanoparticles, into low-loss materials significantly enhances the dielectric loss factor and improves microwave energy absorption.^79^ Susceptors can be placed either in direct contact with the sample or separated from it by the reactor walls. Furthermore, the introduction of magnetic nanoparticles, such as Fe_2_O_3_, NiFe_2_O_4_, Ni, or Fe_3_O_4_, allows them to act as localized microwave absorbers.^322–324^ This approach enables superior microwave coupling in materials that otherwise exhibit poor absorption, thereby expanding the applicability of microwave processing to a broader range of systems.

Hence, the strategic engineering of material composition and the utilization of hybrid or composite systems significantly improve the compatibility between microwave fields and materials. This expands the applicability of microwave processing to a much broader class of functional and structural materials.

### 7.2 Challenges with Scaling-Up of Microwave Sintering

The scaling-up of microwave sintering faces several fundamental challenges that relate to material heterogeneity, sample geometry, microstructure evolution, and thermal instabilities. These issues result in nonuniform heating, cracking and uncontrolled thermal effects. Overcoming these challenges is essential for the adoption of microwave technologies in industries.

The differences in heterogeneous dielectric properties of materials pose a fundamental challenge. Polar and nonpolar molecules respond differently under the microwave fields, some heat rapidly due to dipolar rotation, while others rely on ohmic heating.^325^ Variations in particle size distribution, impurities, and residual stresses can also cause nonuniform dielectric permittivity and dielectric loss,^326–329^ which shift both temperature and frequency.^330^ These nonlinear behaviors complicate process control and lead to uneven heating across the sample.

Sample geometry can also lead to various challenges. For large parts that approach the penetration depth of the material, heating becomes nonuniform, often producing temperature gradients and cracks.^21,194,331^ Exacerbated heat loss imbalance and amplified localized heating can also occur. Additionally, simultaneous sintering of multiple parts requires synchronization of power input and sample movement, which remains a major challenge for continuous large-scale processing.^332^

At the microstructural level, rapid microwave heating promotes densification while suppressing grain growth, which leads to fine-grain structures.^333,334^ However, this type of growth has associated thermal stresses, which can lead to cracks, specifically in porous green bodies, where solvents and binders create stress concentration sites.^335^ Localized overheating also triggers undesired phase formation, thereby weakening mechanical performance.

Thermal effects also present as significant barriers to scaling up. The fast volumetric heating leads to thermal gradients, hotspots, and thermal runaway.^332,336,337^ This occurs due to a decrease in thermal conductivity with increasing temperatures, which creates a positive feedback loop. This loop leads to uncontrolled heating which can melt the sample and cause catastrophic failure. This makes controlling precise temperatures difficult, especially in larger or heterogeneous samples.

Table 8 demonstrates the factors which decide the current potential for scaling up conventional, spark plasma sintering, and microwave sintering techniques. Conventional sintering is well-established and is scalable but suffers from long processing times and high energy costs. Spark plasma sintering offers ultrafast densification but is constrained by die-based geometries and limited throughput. In contrast, microwave sintering provides a middle ground with rapid densification at a significantly lower energy consumption, but scaling up still faces challenges compared to conventional routes.

**Table 8:** Factors Deciding the Current Potential for Scaling up Conventional, Spark Plasma, and Microwave Sintering Techniques, Including Commercialization, Geometry, Time, Cost, and productivity^a^ ^24,338–341^

Technique	Commercialization	Geometry	Time	Cost	Productivity
Conventional Sintering	Most widely used scaled-up process	Able to produce complex shapes	Slow (in the order of several hours)	High energy consumption, higher cost (e.g., to sinter BaTiO_3_, required energy is ≈2800 kJ g^–1^)	Multiple batches simultaneously (≈40 k kg h^–1^ average ceramic output in industrial ovens)
Spark Plasma Sintering	Rotary-based SPS have been proposed for high-throughput	Complex shapes limited by die design	Fast sintering (in the order of seconds)	Medium cost consideration (e.g., to sinter BaTiO_3_, required energy is ≈1050 kJ g^–1^)	Bulk production challenging due to smaller die size (≈120 SPS pellets per day)
Microwave Sintering	Demonstrated with 28–30 GHz gyrotron integrated with self-contained system	Better flexibility through selective and superheating	Moderately fast time (in the order of minutes)	Less expensive, less energy usage (e.g., to sinter BaTiO_3_, required energy is ≈540 kJ g^–1^)	Reduced processing time via volumetric heating (e.g., up to 27–30 pieces/day per worker)

a Adapted from ref 102 Licensed Under CC BY.

Another issue that needs to be addressed is the challenge associated with evaluating energy efficiency in microwave processing, particularly during scale-up. Meaningful assessment requires metrics such as energy per unit mass processed, degree of densification or conversion, as well as throughput-normalized comparisons; however, these are not consistently reported across studies. Previous work by Bermúdez et al. showed that the energy consumption in microwave systems is strongly scale-dependent, with laboratory-scale experiments often overestimating energy requirements.^342^ In addition, system-level analyses conducted by Gallego-Schmid et al. indicate that overall energy consumption and environmental impact depend on factors beyond the heating step alone.^343^

In addition to materials-related constraints, significant challenges arise from the microwave hardware itself. Limitations in cavity design and operating modes often lead to nonuniform electromagnetic field distributions,^344,345^ resulting in localized hotspots and uneven thermal profiles.^81^ Furthermore, conventional magnetron-based systems suffer from inherent frequency instability and fluctuations in power efficiency, which compromise reproducibility and process control. These issues are further compounded by the difficulty of accurate temperature monitoring, where the penetration depth and reactor configuration play critical roles in determining heating uniformity and process reliability.^344^

## 8 Conclusions and Future Perspectives

Future advancements in materials and devices require innovative processing and manufacturing techniques. Microwave-matter interactions help develop new classes of materials. This review introduces microwave processing as a powerful tool for advanced materials processing, including synthesis, sintering, joining, 3D printing, and recycling. It offers advantages in synthesis speed and energy efficiency, allowing for rapid material synthesis through selective and volumetric heating. By leveraging thermal and nonthermal effects, microwaves have demonstrated significant potential in various applications, from advanced ceramics and biomaterials to renewable energy and recycling. These applications benefit from novel materials phases, which are generally inaccessible using conventional methods, as well as enabling greener synthesis, more efficient manufacturing, and supporting a circular economy through material recycling.

Microwave processing has been extensively studied in the food and organic chemistry industries, but its solid-state processing and fundamental effects on the structures and properties of advanced engineering materials are less understood. Compared to conventional heating, microwave processing substantially reduces reaction time and temperature because microwave irradiation can penetrate and uniformly heat the entire sample. This makes the process much faster and more commercially viable. Additionally, microwave processing significantly improves materials properties.

Despite these advantages, an important issue that needs to be addressed across the literature is the inconsistency in reporting of certain terms during microwave processing. A significant issue that must be addressed in the literature is the lack of reporting of a minimum checklist of terms during microwave processing. Several parameters such as frequency, applied power, sample geometry, and temperature measurements are often reported; however, other critical factors, including absorbed power, reflected power, cavity characteristics such as quality factor, and experimental configurations are not clearly and consistently documented. This makes it difficult to effectively compare results across different studies. To ensure compatibility and reproducibility, it is recommended that a more structured approach be adopted, incorporating a set of minimum reporting considerations to address this gap.

Beyond these challenges, there are numerous research opportunities for significant breakthroughs in the synthesis, processing, and manufacturing of materials using microwave radiation. Figure 16 summarizes the key challenges in microwave processing and the corresponding future research directions to address these limitations.

**Figure 16:**
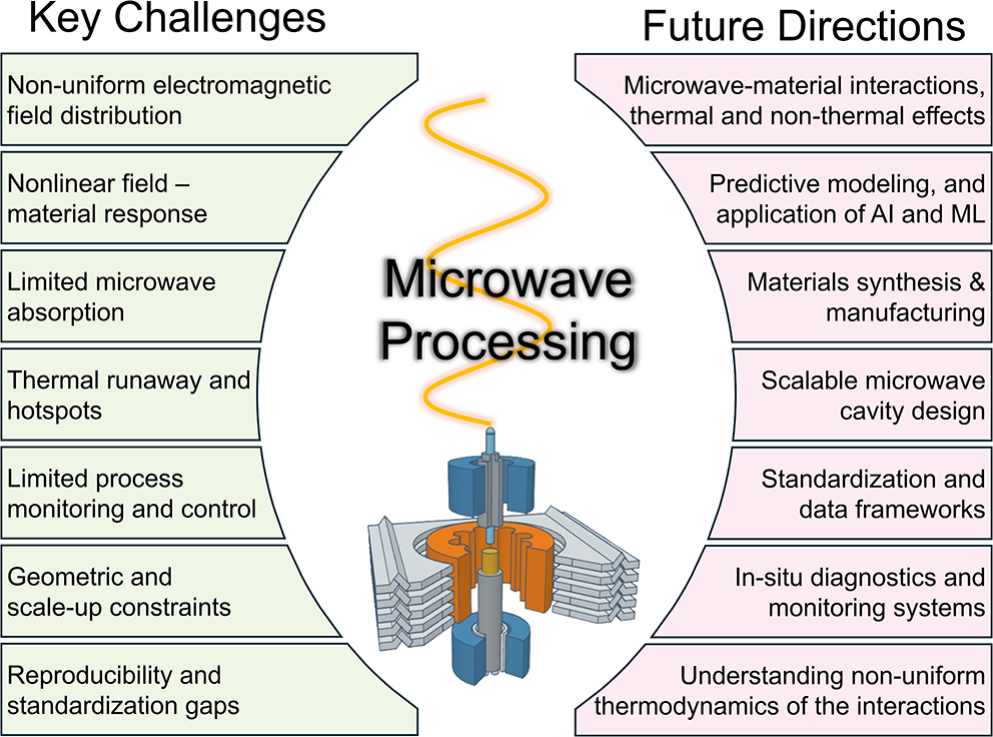
Key challenges in microwave processing and research directions to address these limitations.

These directions are discussed further in detail below:

1 Microwave processing is a green and sustainable fabrication method that can be applied to the synthesis, processing, and manufacturing of various material systems. Microwave processing can revolutionize the future of many technologies, such as renewable energy (e.g., thermoelectrics, piezoelectrics, thermomagnetics, and photovoltaics), advanced and high-temperature materials (e.g., MXenes, advanced ceramics, and materials for hypersonic and extreme environments), soft and hard magnets, biomaterials and biomedical research, catalysts, nanoelectronics, and microwave absorption materials. Beyond just energy savings, microwave processing has enabled the synthesis of far-from-equilibrium material systems, offering pathways to explore novel microstructures and phases, inaccessible through conventional methods. It can be used for any current or future applications that require nonequilibrium properties, amorphous structures, high-entropy alloys, or entropy-engineered materials.
2 A critical need in microwave processing is the development of standardized frameworks for evaluating the energy efficiency of microwave systems. To enable meaningful comparisons with other processing methods, consistent metrics such as energy per unit mass processed, degree of densification or conversion, and throughput-normalized data are required. Furthermore, incorporating system-level considerations such as power delivery, coupling efficiency, and scale-dependent effects will be essential for enabling meaningful comparison and facilitating future industrial adoption.
3 (A major scientific challenge in microwave synthesis remains the elusive understanding of its nonthermal effects. While numerous studies have confirmed their existence, a fundamental and deterministic distinction between thermal and nonthermal effects has not yet been established; this remains a necessity that extends beyond purely rigorous theoretical modeling. Developing a standardized validation approach is an essential future direction in the field of microwave processing. Further studies require reliable in situ temperature mapping, comparisons with other techniques using matched thermal histories and identical heating rates, and a clear differentiation of the effects of localized hot spots.
4 In order to deepen the understanding of material properties and identify nonthermal effects while improving the process, the incorporation of Artificial Intelligence (AI) and Machine Learning (ML) tools is crucial. Recently, AI has been used to predict the temperature profile of microwave processing,^346^ and ML tools have been used to improve microwave heating performance.^347^ Future research directions in this field should focus on understanding the physics of nonthermal effects, clarifying their existence, and reliably distinguishing between thermal and nonthermal effects. Additionally, emphasis should be placed on the in situ prediction of dielectric properties under microwaves, as well as process monitoring and the prediction of defects and temperature profiles.
5 A fundamental challenge in microwave processing lies in effectively heating materials with poor microwave absorption. This problem stems from the nonlinear, exponential nature of microwave heating, where low temperatures result in poor heating, but higher temperatures lead to faster heating as the material’s dielectric properties change. This nonlinearity can potentially lead to thermal runaway beyond critical temperatures. Materials highly susceptible to microwave coupling include ionic compounds with polarizable structures, those doped with conductive phases, or materials in a molten or ionically conductive state. Therefore, engineering both materials and techniques for selective microwave absorption is highly demanded, specifically focusing on materials that do not conventionally absorb microwaves. This strategy involves intentionally introducing specific dopants or engineering defects to act as susceptors, allowing for the predictable tuning of the material’s properties.
6 Microwave sintering is incredibly fast and requires minimal energy consumption. For materials with high melting points, microwave thermal and nonthermal effects can be intentionally used to control sintering conditions, final density, and grain size.
7 Microwave processing has emerged as a powerful tool that enables the formation of unique phases and structures often inaccessible through conventional routes. The localized and rapid heating in microwave processing promotes high supersaturation, rapid nucleation, and phase-selective crystallization, giving researchers the ability to obtain precise structural control. Additionally, microwave processing enables the generation of various defects such as vacancies, solid solutions, and dislocations within the materials. Using in situ characterization tools such as Raman thermometry for a noncontact temperature profile mapping and Pair Distribution Function (PDF) analysis enables tracking of local disorders in real time.
8 Future research should also investigate the effect of microwaves on thermodynamically nonequilibrium microstructural changes and the resulting properties. Specifically, understanding how these changes affect the electronic and thermal transport, as well as the magnetic and optical properties of both thin-film and bulk materials, ultimately leading to the development of robust process-structure–property relationships among advanced functional materials.
9 Microwave processing can be used as a powerful manufacturing technique, such as microwave-assisted extrusion, microwave joining (brazing and welding) of both similar and dissimilar materials, 3D printing, as well as recycling and waste management.
10 Future advancements in microwave processing also require rigorous modeling and finite element simulations of the electromagnetic field inside the waveguide and appropriate microwave system design. While preliminary studies have explored the influence of the cavity geometry, configuration of the waveguide, and the effect of material properties on the field distribution, most research remains in the modeling phase. The uniformity of the electromagnetic field on the sample, the effect of the synthesis environment, penetration depth, and the influence of separate electric and magnetic fields need to be further analyzed experimentally. In particular, a significant gap exists in systematic experimental studies that directly correlate spatial variations in electric and magnetic field distributions with processing outcomes such as reaction kinetics, defect formation, and phase evolution.
11 The implementation of digital twins, which are high-fidelity virtual models, can help improve the understanding of microwave systems and their interaction with materials. Although the application of digital twins in microwave materials processing remains relatively unexplored, it represents a promising direction for future research. The incorporation of these digital replicas could enable the simulation of outcomes and the visualization of complex phenomena that are generally difficult to observe, such as active temperature monitoring, analysis of electromagnetic field absorption, and deeper insight into microwave–materials interactions. This approach may also enable the integration of in situ monitoring and real-time feedback loops for autonomous operation and data-driven process optimization. Nevertheless, computational modeling of complex systems such as microwave processing is challenging. These challenges mainly originate from the three-dimensional multiphysics coupling of electromagnetic waves with thermal and electrical properties, as well as chemical bonding. In addition, the nonlinearity of behavior and properties, the multiscale nature of the process (spanning from atoms and chemical bonds to microstructures), data uncertainty, and high computational costs limit extensive computational modeling of microwave processing.
12 Another challenge in the processing of materials by microwaves is the lack of standardized reporting of experimental parameters. Future studies should aim to establish consistent reporting practices, including parameters such as frequency, power, sample geometry, atmosphere, and temperature calibration, while developing methods to quantify parameters such as absorbed power, reflected power, cavity quality factor, and the role of susceptors. Such standardization would improve the comparability and reproducibility of studies across the field.
13 Developing predictive frameworks that link frequency, dielectric loss, penetration depth, sample geometry, and thermal properties is an important future research direction. These frameworks are essential for predicting heating uniformity, hot-spot susceptibility, and thermal runaway. Such coupled approaches could guide the optimization of microwave processing conditions and significantly improve process reliability.
14 Finally, all innovations in the field of microwave processing need to be translated from the lab to an industrial scale. Achieving this challenging goal will require rigorous research and development efforts from both academia and industry.
